# Systematic Profiling of Poly(A)+ Transcripts Modulated by Core 3’ End Processing and Splicing Factors Reveals Regulatory Rules of Alternative Cleavage and Polyadenylation

**DOI:** 10.1371/journal.pgen.1005166

**Published:** 2015-04-23

**Authors:** Wencheng Li, Bei You, Mainul Hoque, Dinghai Zheng, Wenting Luo, Zhe Ji, Ji Yeon Park, Samuel I. Gunderson, Auinash Kalsotra, James L. Manley, Bin Tian

**Affiliations:** 1 Department of Microbiology, Biochemistry and Molecular Genetics, Rutgers New Jersey Medical School, Newark, New Jersey, United States of America; 2 Rutgers Cancer Institute of New Jersey, Newark, New Jersey, United States of America; 3 Rutgers Graduate School of Biomedical Sciences, Newark, New Jersey, United States of America; 4 Department of Molecular Biology and Biochemistry, Rutgers University, Piscataway, New Jersey, United States of America; 5 Departments of Biochemistry and Medical Biochemistry, University of Illinois, Urbana, Illinois, United States of America; 6 Department of Biological Sciences, Columbia University, New York, New York, United Staes of America; University of Texas Medical School, UNITED STATES

## Abstract

Alternative cleavage and polyadenylation (APA) results in mRNA isoforms containing different 3’ untranslated regions (3’UTRs) and/or coding sequences. How core cleavage/polyadenylation (C/P) factors regulate APA is not well understood. Using siRNA knockdown coupled with deep sequencing, we found that several C/P factors can play significant roles in 3’UTR-APA. Whereas Pcf11 and Fip1 enhance usage of proximal poly(A) sites (pAs), CFI-25/68, PABPN1 and PABPC1 promote usage of distal pAs. Strong cis element biases were found for pAs regulated by CFI-25/68 or Fip1, and the distance between pAs plays an important role in APA regulation. In addition, intronic pAs are substantially regulated by splicing factors, with U1 mostly inhibiting C/P events in introns near the 5’ end of gene and U2 suppressing those in introns with features for efficient splicing. Furthermore, PABPN1 inhibits expression of transcripts with pAs near the transcription start site (TSS), a property possibly related to its role in RNA degradation. Finally, we found that groups of APA events regulated by C/P factors are also modulated in cell differentiation and development with distinct trends. Together, our results support an APA code where an APA event in a given cellular context is regulated by a number of parameters, including relative location to the TSS, splicing context, distance between competing pAs, surrounding cis elements and concentrations of core C/P factors.

## Author Summary

A gene can express multiple isoforms varying in the 3’ end, a phenomenon called alternative cleavage and polyadenylation, or APA. Previous studies have indicated that most eukaryotic genes display APA and the APA profile changes under different physiological and pathological conditions. However, how APA is regulated in the cell is unclear. Here using gene knockdown and high throughput sequencing we examine how APA is regulated by factors in the machinery responsible for cleavage and polyadenylation as well as factors that play essential roles in splicing. We identify several factors that play significant roles in APA in the last exon, including CFI-25/68, PABPN1, PABPC1, Fip1 and Pcf11. We also elucidate how cleavage and polyadenylation events are regulated in introns and near the transcription start site. We uncover a group of APA events that are highly regulated by core factors as well as in cell differentiation and development. We present an APA code where an APA event in a given cellular context is regulated by a number of parameters, including relative location to the transcription start site, splicing context, distance between competing pAs, surrounding cis elements and concentrations of core cleavage and polyadenylation factors.

## Introduction

Pre-mRNA cleavage and polyadenylation (C/P) is a 3’ end processing mechanism employed in eukaryotes for expression of almost all protein-coding transcripts and long non-coding RNAs by RNA polymerase II (RNAPII)[1, 2]. The site for C/P, commonly known as the polyA site or pA, is defined by both upstream and downstream cis elements [3, 4]. As with core RNAPII promoters [5], core C/P signals are proving to be complex. In mammals, upstream elements include the polyadenylation signal (PAS), such as AAUAAA, AUUAAA, or close variants, located within ~40 nucleotides (nt) from the pA; UGUA elements, typically located upstream of the PAS; and U-rich elements located around the PAS. Downstream elements include U- and GU-rich elements, located within ~100 nt downstream of the pA.

Most mammalian genes express alternative cleavage and polyadenylation (APA) isoforms [6–9]. APA in the 3’ untranslated region (3’UTR), called 3’UTR-APA, leads to isoforms with different 3’UTR lengths. Because the 3’UTR plays an important role in aspects of mRNA metabolism, such as subcellular localization, stability, and translation, 3’UTR-APA can impact the post-transcriptional control of gene expression. In addition, about 40% of mammalian genes display APA in upstream introns and internal exons [10], leading to changes of both coding sequences (CDSs) and 3’UTRs. This type of APA is called CDS-APA herein. Studies have shown that the APA pattern of genes is tissue-specific [7, 9, 11, 12], and is regulated under various conditions, such as cell proliferation, differentiation and development [8, 13–16] and response to extracellular signals [17]. However, despite recent advances, the molecular mechanisms that regulate APA are still poorly understood (see [4, 18–20] for reviews).

The C/P machinery in mammalian cells is composed of over 20 core factors [21]. Some form subcomplexes, including the Cleavage and Polyadenylation Specificity Factor (CPSF), containing CPSF-160, CPSF-100, CPSF-73, CPSF-30, Fip1, and WDR33; the Cleavage stimulation Factor (CstF), containing CstF-77, CstF-64, and CstF-50; the Cleavage Factor I (CFI), containing CFI-68 or CFI-59 and CFI-25; and the Cleavage Factor II (CFII), containing Pcf11 and Clp1. Single proteins involved in C/P include Symplekin, poly(A) polymerase (PAP), nuclear poly(A) binding protein (PABPN), RBBP6, and RNAPII (specifically the C-terminal domain of its largest subunit). In addition, protein phosphatase 1α (PP1α) and protein phosphatase 1β (PP1β) are present in the C/P complex and homologous to yeast C/P factors [22], but their functions in 3’ end processing are yet to be established. Since the initial study indicating that CstF-64 can regulate APA of the IgM heavy chain gene pre-mRNA during B cell maturation [23], a growing number of core C/P factors have been shown to impact pA choice, such as CFI factors [24–26], PABPN1 [27, 28], CstF-64τ [29, 30], Fip1 [31] and RBBP6 [32]. Whether other C/P factors are involved in APA is not known, and to what extent different C/P factors differentially modulate APA has not been systematically examined.

C/P can also be regulated by splicing (reviewed in [33]), which has long been thought to help define the 3’ terminal exon [34]. U1 snRNP (or U1) has been shown to suppress cryptic pA usage near the transcription start site (TSS)[35], which may be attributable to its inhibitory activity on poly(A) polymerase α (PAPα)[36]. The interplay between U1 and C/P has recently been implicated in controlling expression of sense vs. antisense transcripts from bidirectional promoters [37, 38]. Interestingly, mild attenuation of U1 that does not inhibit splicing was found to regulate 3’UTR length via APA [39]. This mechanism, dubbed telescripting, has been implicated in pre-mRNA shortening during rapid and transient transcriptional upregulation in neuronal activation when there is shortage of U1. By contrast, no global regulation of APA has been associated with U2 snRNP (or U2), despite various interactions between U2 factors and the C/P machinery [40, 41].

Here using C2C12 cells and siRNA-based knockdown (KD) of individual core C/P factors, we examine the role of C/P factors in global APA regulation. We also reduce the expression and/or activity of several core splicing factors in order to understand the role of splicing in APA. In addition, we investigate expression of sense and antisense transcripts using pAs near the TSS when C/P or splicing factors are inhibited. Lastly, we examine how APA events regulated by C/P factor KDs are related to those taking place in cell differentiation. Altogether, our results reveal multiple regulatory rules of APA and show that modulation of core RNA processing factor levels provides a powerful mechanism to control global APA and that different factors have distinct impacts on pA usage.

## Results

### Global analysis of APA upon inhibition of core C/P and splicing factors

**Fig 1:**
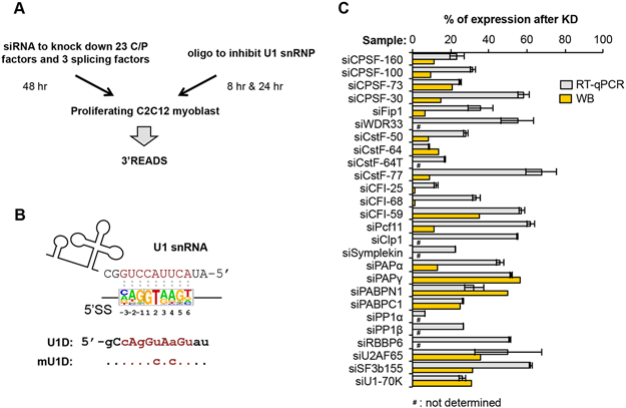
Systematic analysis of APA events modulated by C/P and splicing factors. **(A)** Experimental design. Proliferating C2C12 myoblast cells were transfected with siRNAs against 23 C/P factors and 3 splicing factors for 48 hr, followed by APA analysis using 3’ region extraction and deep sequencing (3’READS). Sequences of the siRNAs used for knockdown or control are listed in S1 Table. For direct inhibition of U1 snRNP (U1) activity, C2C12 cells were transfected with U1D or mutant U1D (mU1D) oligonucleotides (oligos, shown in (B)) for 8 hr or 24 hr, followed by 3’READS analysis. **(B)** Oligos used to study U1 functions. Top, the 5’ end region of U1 snRNA and a consensus sequence surrounding 5’ splice site (5’SS, -3 nt to +6 nt, based on all annotated 5’SS in the mouse genome), are shown to illustrate the base-pairing between U1 snRNA and 5’SS sequence; Bottom, U1D or mutant U1D (mU1D) sequences. Locked nucleic acids are in upper case and 2'-O-methyl nucleotide in lower case. Nucleotides shown in red correspond to the -3 to +6 region surrounding the 5’SS. mU1D differs from U1D in two nucleotides as indicated. **(C)** Gene expression of different factors after 48 hr of siRNA knockdown (KD), as measured by reverse transcription-quantitative PCR (RT-qPCR) or Western Blot (WB). Error bars for RT-qPCR are standard deviation based on two technical replicates. Some KD samples were not analyzed by WB, as indicated. See S1 Fig for WB images.

To examine the role of core C/P factors in APA regulation, we set out to knock down by siRNA the expression of each of the known factors (Fig 1 and S1 Table, see Introduction), including CPSF-160, CPSF-100, CPSF-73, CPSF-30, Fip1, WDR33, RBBP6, CstF-77, CstF-64, CstF-64τ, CstF-50, CFI-25, CFI-59, CFI-68, Pcf11, Clp1, PAPα, PAPγ, PABPN1, and Symplekin. In addition, we designed siRNAs for several C/P complex-associated factors [22], including PP1α, PP1β, and poly(A) binding protein C1 (PABPC1). Since a large fraction of pAs are located in introns and are regulated in development and differentiation [10], we also included siRNAs for several core splicing factors that have been previously implicated in regulation of C/P through various mechanisms [33], including U1 factor U1-70K, U2 factor SF3b155 and U2-associated factor U2AF65, Moreover, because functional inhibition of U1 was previously shown to cause global regulation of APA in both introns [35] and 3’UTRs [39], we included in our experiments an oligonucleotide (oligo) that can base pair with the 5’ end region of U1 snRNA involved in 5’ splice site (5’SS) recognition (Fig 1B). This oligo, named U1 domain oligo (U1D), is composed of locked nucleic acids and 2’-O-methylated nucleotides, and has been shown to base pair efficiently with U1 snRNA, thereby functionally knocking down U1 [42]. An oligo that had the same chemical composition but contained two mutations at critical positions for base pairing with U1 snRNA, named mutant U1D oligo (mU1D, Fig 1B), was used as a control.

We chose C2C12 myoblast cells for this study because we previously found that during differentiation of C2C12 cells into myotubes, APA isoforms using proximal pAs (relative to the 5’ end of gene), including those in 3’UTRs and introns, are relatively downregulated, while distal pAs are relatively upregulated [10, 14]. Therefore, by using C2C12 cells, we could compare APA events regulated by C/P or splicing factors with those taking place naturally during cell differentiation. For knockdown (KD) experiments, we selected siRNAs that could reduce their target mRNA expression by >30% in C2C12 cells, as determined by reverse transcription-quantitative PCR (RT-qPCR)(Fig 1C; primers listed in S3 Table). For the factors for which we had antibodies, Western Blot analysis was also carried out to confirm success of KD (Fig 1C and S1 Fig; antibody information listed in S2 Table). We extracted total RNA 48 hr after siRNA transfection, or 8 or 24 hr after transfection with U1D or mU1D. To obtain a global view of APA, we applied 3’ Region Extraction And Deep Sequencing (3’READS), a method recently developed by our lab to sequence the 3’ end region of poly(A)+ RNAs genome-wide [10]. The statistics of sequencing reads aligned to pAs are shown in S4 Table.

### Regulation of 3’UTR-APA

**Fig 2:**
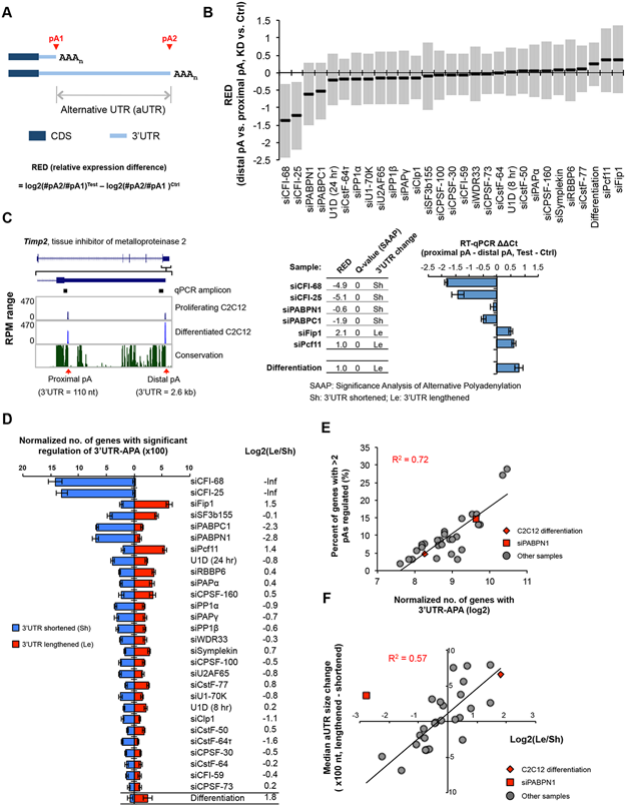
3’UTR-APA. **(A)** Schematic of 3’UTR-APA that results in alternative 3’UTR isoforms. The region between two pAs, pA1 and pA2, is called alternative UTR (aUTR). AAA_n_, poly(A) tail. CDS and 3’UTR are indicated. Regulation of APA is represented by RED (relative expression difference), whose formula is shown in the figure. #pA1 and #pA2 are numbers of poly(A) site-supporting (PASS) reads for pA1 and pA2, respectively. Test and Ctrl are test and control samples, respectively. **(B)** RED values for different samples. The top two most abundant APA isoforms based on the number of PASS reads of each gene were analyzed. Only genes with > = 20 PASS reads for proximal and distal pAs combined were used. The median RED value of each sample is shown as a thick black line and interquartile range (between the 25^th^ and 75^th^ percentiles) is indicated by a gray box. **(C)** An example of 3’UTR-APA regulation. Left, APA isoforms of *Timp2*. The gene structure and the zoomed-in 3’-most exon are shown on the top. Two pAs are indicated, and their 3’UTR lengths and RPM values in proliferating and differentiated C2C12 cells are shown. Sequence conservation of the shown region (based on mammals) is indicated, with the height of line reflecting the degree of conservation. RT-qPCR amplicons to study APA regulation are indicated. Middle, regulation of the two 3’UTR-APA isoforms of *Timp2* in several samples as indicated. RED values (distal pA vs. proximal pA, knockdown (KD) vs. siCtrl or differentiated cells vs. proliferating cells), q-values (Significance Analysis of Alternative Polyadenylation, SAAP, see Materials and Methods for detail), and direction of 3’UTR change (shortened, sh; or lengthened, Le) are indicated. Right, RT-qPCR analysis of 3’UTR-APA isoforms of *Timp2* in several samples. RT-qPCR ΔΔCycle threshold (Ct) values were calculated by comparing Ct difference between proximal and distal amplicons in test vs. ctrl samples. Test is a sample from knockdown or differentiated cells, and ctrl is a sample from siCtrl or proliferating cells. Note the samples used for RT-qPCR analysis were not the same as those used for 3’READS. Error bars are standard deviation based on two replicates. **(D)** Normalized number of genes with regulated 3’UTR-APA in each sample as examined by GAAP. Normalized number is based on (observed value—expected value), and zero is used if a value is negative. Red and blue bars represent genes with lengthened 3’UTRs (Le, distal pA isoform upregulated) and shortened 3’UTRs (Sh, proximal pA isoform upregulated), respectively. Q-value < 0.05 (SAAP) was used to select genes with significant 3’UTR-APA regulation. Only the top two most abundant APA isoforms (based on the number of PASS reads) of each gene were used for this analysis. Error bars are standard deviation based on 20 times of bootstrapping. Samples are sorted by the total number of genes with 3’UTR-APA changes. Data for C2C12 differentiation are shown at the bottom for comparison. Log2(Le/Sh) is log2(ratio) of the number of Le genes to the number of Sh genes. **(E)** Correlation between normalized no. of genes with 3’UTR-APA (Le+Sh in (D)) and percent of genes with >2 pAs regulated (see S3 Fig). C2C12 differentiation data and siPABPN1 data are shown in red and others in gray. R^2^ is based on linear regression of all dots. **(F)** Correlation between log2(Le/Sh) and median aUTR size change. Data from all samples shown in (D) except the siCFI-68 and siCF-25 samples are plotted, with C2C12 differentiation data and siPABPN1 data shown in red diamond and square, respectively, and others in gray dots. R^2^ is based on linear regression of all gray dots. Median aUTR size change was calculated by the median aUTR size of lengthened 3’UTRs minus that of shortened 3’UTRs. Each gene was calculated once.

We first examined APA changes in 3’UTRs, which results in differential expression of isoforms with different 3’UTR lengths (illustrated in Fig 2A). To simplify analysis, we focused on the relative expression changes of the two most abundant 3’UTR isoforms of each gene. Using relative expression difference (RED) between proximal and distal pA isoforms in KD vs. control samples (RED = difference in log2(ratio) of read numbers of two pA isoforms between two samples; illustrated in Fig 2A), we found that several KD samples showed significant upregulation of proximal pAs (negative RED median of all genes), including those of siCFI-68, siCFI-25, siPABPN1, and siPABPC1, and several others had the opposite trend (positive RED median), including the siFip1 and siPcf11 samples (Fig 2B). Differentiation of C2C12 also had a positive RED median, consistent with our previous finding of 3’UTR lengthening in this process [10, 14]. To statistically examine APA regulation, we developed a method named Significance Analysis of Alternative Polyadenylation (SAAP), which evaluated the significance of a RED score using the read distribution of APA isoforms in two comparing samples. A q-value was calculated based on data randomization (illustrated in S2 Fig and see Materials and Methods for detail), which indicated the false discovery rate (FDR). An example gene *Timp2* (tissue inhibitor of metalloproteinase 2) is shown in Fig 2C, whose APA was strongly affected by KDs of several factors: siCFI-25, siCFI-68, siPABPN1, and siPABPC1 resulted in upregulation of the proximal pA isoform (3’UTR size = 110 nt), whereas siPcf11 and siFip1 led to upregulation of the distal pA isoform (3’UTR size = 2.6 kb). SAAP analysis of the two isoforms showed that APA regulations in these samples, as well as in C2C12 differentiation, were all significant (q-value = 0, Fig 2C, middle). This result was confirmed by reverse transcription-quantitative PCR (RT-qPCR) using amplicons before and after the proximal pA (Fig 2C, right). Notably, APA of human *Timp2* has also been reported in several studies [25, 26], suggesting the importance of its regulation across species.

We next wanted to compare the extent of APA regulation across samples. We carried out KD experiments in several batches, each with a negative control. However, because samples from different batches were processed and sequenced at different times, systematic biases (“batch effect”) could be introduced into the data. In addition, samples with different levels of sequencing depth could lead to variable sensitivity for APA detection, making them not directly comparable. To address these issues, we developed a computational pipeline, named Global Analysis of Alternative Polyadenylation (GAAP), in which we randomly sampled reads to control for sequencing depth and permutated treatment and control data to obtain expected data (S2C Fig). Both expected and observed data were subject to SAAP for identification of significantly regulated APA events. The number of regulated APA events from the observed data was subtracted by that from the expected data to obtain normalized number of APA events. As such, each sample was internally controlled and cross-batch comparison was more feasible.

Using GAAP, we found that almost all KD samples, including inhibition of U1, resulted in considerable changes of 3’UTR-APA (Fig 2D). The top ten treatments with respect to normalized number of genes with altered 3’UTR-APA were, in the order of number, siCFI-68, siCFI-25, siCPSF-160, siFip1, siSF3b155, siPABPC1, siPABPN1, siPcf11, U1D (24 hr) and siRBBP6. To gauge the overall trend of APA direction, i.e., 3’UTR lengthening or shortening, we calculated the ratio of the number of genes with lengthened 3’UTRs (Le) to the number of genes with shortened 3’UTRs (Sh), or log2(Le/Sh)(Fig 2D). We found that siCFI-25 and siCFI-68 led to the most substantial 3’UTR shortening, with log2(Le/Sh) values being negative infinite after normalization to expected values (Fig 2D). This result is consistent with previous reports showing significant 3’UTR shortening after CFI-25 or CFI-68 KD [24, 43]. KDs of the two PABPs also led to considerable 3’UTR shortening, with log2(Le/Sh) = -2.8 and -2.3, respectively, for siPABPN1 and siPABPC1. While the siPABPN1 result is in line with previous reports [27, 28], this is the first time PABPC1 is found to play a global role in APA and the extent of its modulation of 3’UTR-APA appeared to be similar to that of PABPN1. We found that siFip1 and siPcf11 led to the most substantial 3’UTR lengthening, with log2(Le/Sh) = 1.5 and 1.4, respectively. In comparison, the log2(Le/Sh) value was 1.8 for C2C12 differentiation (Fig 2D). Consistent with previous reports [39], we also found that reduction of U1 activity by U1D (24 hr) or siU1-70K led to 3’UTR shortening. However, the 3’UTR-APA changes in these samples appeared modest compared to the more significant C/P factor KD samples described above. It is worth noting that a similar analysis using the two most significantly regulated pA isoforms of each gene gave essentially identical results (S3A Fig), confirming the robustness of our findings.

As expected, the GAAP result (Fig 2D), which indicates the number of genes with significant APA regulation, correlated with the RED result (Fig 2B), which indicates the extent of APA regulation. We further found that the GAAP result also correlated with the number of significantly regulated pA isoforms per gene (Fig 2E and S3 Fig), i.e., samples with more genes having 3’UTR-APA regulation tended to have a greater number of regulated isoforms per gene. For example, in siCFI-25 and siCFI-68 samples, ~30% of genes had more than two pA isoforms significantly regulated (S3 Fig). This result indicates that multiple pAs are interrelated in regulation of their usage.

The region between two regulated pAs is called alternative UTR (aUTR; illustrated in Fig 2A). We found that when there was global 3’UTR lengthening, the lengthened aUTRs were generally longer than the shortened ones, and vice versa (S3 Fig). Consistently, there was a good correlation between log2(Le/Sh) and median aUTR size difference between lengthened and shortened 3’UTRs (R^2^ = 0.57, excluding siPABPN1, Fig 2F; note that siCFI-68 and siCFI-25 were not used for this analysis because of their negative infinite log2(Le/Sh) values). The 3’UTR regulation in C2C12 differentiation also followed this trend (Fig 2F). However, the siPABPN1 sample was a notable exception, in which shortened aUTRs were generally shorter than lengthened ones despite a global trend of 3’UTR shortening (Fig 2F and S3 Fig). This result indicates that change of 3’UTR size is generally coupled with the extent of 3’UTR-APA regulation, and the APA regulatory mechanism by PABPN1 is distinct from that of other factors.

### Regulation of CDS-APA

**Fig 3:**
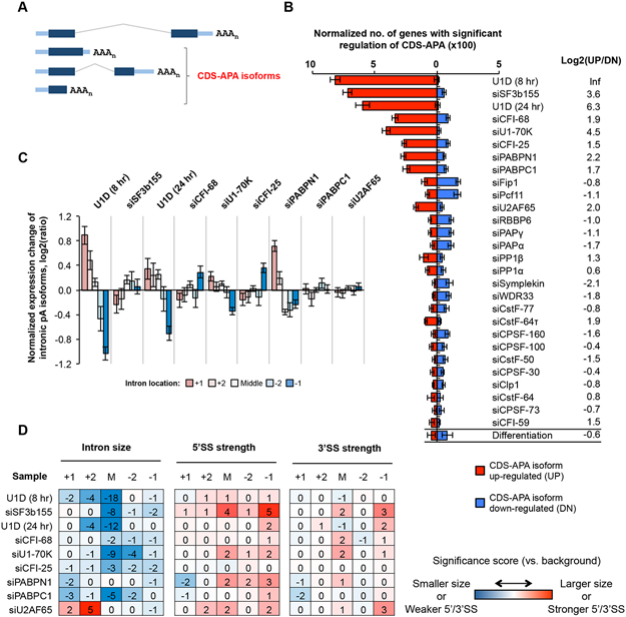
CDS-APA. **(A)** Schematic of CDS-APA. **(B)** Normalized number of genes with regulated CDS-APA as examined by GAAP. Red and blue bars represent genes with upregulated CDS-APA isoforms (UP) and downregulated isoforms (DN), respectively. All CDS-APA isoforms were combined and compared to all 3’-most exon isoforms combined by SAAP. Q-value < 0.05 (SAAP) was used to select genes with significant CDS-APA regulation. Error bars are standard deviation based on 20 times of bootstrapping. Samples are sorted by the total number of genes with CDS-APA changes. Data for C2C12 differentiation are shown at the bottom for comparison. Log2(UP/DN) is log2(ratio) of the number of UP genes to the number of DN genes. **(C)** Normalized expression changes of intronic pA isoforms in several samples. Introns were divided into first (+1), second (+2), last (-1), and second to last (-2), and middle (between +2 and -2 introns) groups. Only genes with ≥4 introns and only pA isoforms with ≥10 PASS reads in two comparing samples combined were analyzed. Expression changes are log2(ratio) of PASS reads in test sample vs. control sample. Values for five intron groups were normalized by mean-centering to reveal bias of intron location. Error bars are standard error of mean. **(D)** Features of introns containing pAs of upregulated isoforms, including intron size, and 5’ and 3’ splice site (SS) strengths. Numbers are significance score (SS), which was calculated by –log_10_(*P*)*S, where *P* was based on the Wilcoxon rank sum test comparing an intron set of interest with a background set, and S = 1 when the intron set of interest had a larger median value (intron size, 5’SS strength or 3’SS strength) than the background set or -1 otherwise. The background set was derived from introns that contained detectable pA isoform expression in control samples. Introns were divided into five groups based on location, as in (C). The SS data are colored according to the color scheme shown in the graph.

We next examined CDS-APA events, as defined by their pA location in introns or exons upstream of the 3’-most exon (illustrated in Fig 3A). Using GAAP, we found that KDs of several splicing factors, such as by siU1-70K and siSF3b155, as well as inhibition of U1 by U1D (8hr and 24 hr), led to substantial changes of the CDS-APA pattern (Fig 3B). By contrast, siU2AF65 resulted in much fewer regulated events. All splicing factor KD samples showed overall upregulation of CDS-APA isoforms, as indicated by their log2(UP/DN) values (UP, number of genes with upregulated CDS-APA isoforms; DN, number of genes with downregulated CDS-APA isoforms)(Fig 3B). Notably, the upregulated CDS-APA isoforms were generally expressed at low levels in control cells (relative abundance <10%, S4 Fig). Taken together, these results indicate that C/P in regions upstream of the 3’-most exon is generally inhibited by splicing and this mechanism is in effect under normal conditions.

KDs of several C/P factors also led to regulation of many CDS-APA events, including siCFI-25, siCFI-68, siPABPN1, siPABPC1, siFip1 and siPcf11 (Fig 3B). While siCFI-25, siCFI-68, siPABPN1, and siPABPC1 resulted in overall upregulation of CDS-APA isoforms, siFip1 and siPcf11 samples showed the opposite trend. This difference between these two sets of KD samples mirrors that for 3’UTR-APA regulation (Fig 2), implying that regulations of CDS-APA and 3’UTR-APA events by these factors are mechanistically related.

Most pAs of CDS-APA isoforms were located in introns (>90%, S4 Fig). We next asked whether introns containing regulated APA events had special features. Focusing on KD samples with substantial regulations, we analyzed expression changes of the isoforms using pAs in the first (+1), second (+2), second to last (-2), last (-1), or middle (M, not the first two or last two) introns. We found that, in U1D (8 hr), U1D (24 hr), siU1-70K and siPABPN1 samples, APA isoforms using pAs in 5’ end introns tended to be relatively upregulated compared to those in 3’ end introns (Fig 3C). By contrast, isoforms using pAs in the last intron tended to be upregulated in siCFI-68 and siCFI-25 samples (Fig 3C). On the other hand, no obvious location bias could be discerned for upregulated intronic APA isoforms in the siSF3b155, siPABPC1, or siU2AF65 samples (Fig 3C), nor for downregulated intronic APA isoforms in siFip1 or siPcf11 samples (S5 Fig).

We found that the middle introns (not the first two or last two introns) that contained pAs of upregulated isoforms by U1 inhibition or siSF3b155 tended to be much smaller than other middle introns (*P* = 1x10^-8^-10^-18^, Wilcoxon test, Fig 3D). In addition, the middle and last introns containing pAs upregulated by siSF3b155 tended to have stronger 5’SS (*P* = 1x10^-4^ and 1x10^-5^, respectively) than their respective control introns (Fig 3D). Because intron size and 5’SS strength are relevant to splicing kinetics, these results indicate that splicing activity has a substantial influence on C/P in introns. By contrast, 3’SS in general did not appear to play a major role in intronic C/P in the samples analyzed in this study, except for some modest significance for the last introns containing upregulated pAs by siSF3b155 and siU2AF65 (Fig 3D). No significant intron features could be identified for intronic APA isoforms downregulated by siFip1 or siPcf11 (S5 Fig), suggesting that the primary mechanism for their regulation is through modulation of C/P activity.

### Regulation of C/P events near the TSS

**Fig 4:**
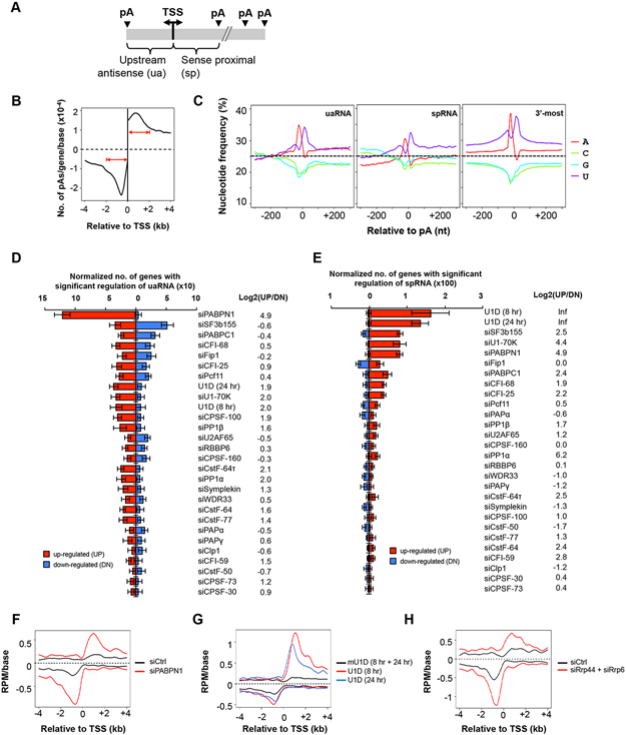
C/P events around the transcriptional start site (TSS). **(A)** Schematic of C/P events around the TSS. uaRNA and spRNA are upstream antisense and sense proximal RNAs, respectively. **(B)** Distribution of uaRNA and spRNA pAs utilized in control C2C12 cells. In this study, we required that the pA of a uaRNA/spRNA was within 2 kb from the TSS, a uaRNA did not overlap with any known protein-coding genes, and the pA of an spRNA was not in the 3’-most exon or in a single-exon gene. **(C)** Nucleotide frequency profiles around pAs of uaRNAs (left) and spRNAs (middle) and around 3’-most pAs (right). Dotted lines indicate the 25% value. **(D)** Normalized number of genes with regulated uaRNA expression. Red and blue bars represent genes with upregulated (UP) and downregulated (DN) uaRNA expression, respectively. Log2(UP/DN) is log2(ratio) of the number of UP genes to the number of DN genes. All uaRNAs were combined and compared by SAAP with all sense strand transcripts whose pAs were beyond 2 kb from the TSS. Q-value < 0.05 (SAAP) was used to select genes with a significant uaRNA expression difference. Error bars are standard deviation based on 20 times of bootstrapping. Samples are sorted by the total number of genes with uaRNA expression change. **(E)** Normalized number of genes with regulated spRNA expression. Red and blue bars represent genes with upregulated (UP) and downregulated (DN) spRNA expression, respectively. All spRNAs were combined and compared to all sense strand transcripts whose pAs were beyond 2 kb from the TSS by SAAP. Q-value < 0.05 (SAAP) was used to select genes with significant spRNA expression difference. Error bars are standard error of mean based on 20 times of bootstrap sampling. Samples are sorted by the total number of genes with spRNA expression changes. Log2(UP/DN) is log2(ratio) of the number of UP genes to the number of DN genes. **(F-H)** Metagene plots of uaRNA and spRNA expression in siPABPN1 (F), U1D (8 hr and 24 hr, G), and siRrp44 + siRrp6 (H) samples. Expression is represented by reads per million (RPM, poly(A) site-supporting reads only) at pA positions.

The enrichment of pAs in the 5’ end introns for upregulated isoforms in siPABPN1 and U1D samples prompted us to examine C/P events near the promoter. Since a large fraction of RNAPII promoters in mammalian cells are bidirectional, leading to both sense and antisense transcripts (reviewed in [44, 45] and illustrated in Fig 4A), we examined C/P events in both sense and antisense orientations around the TSS. Consistent with previous reports [37, 38], we found that in our control samples, the pAs of upstream antisense transcripts, termed uaRNAs or PROMPTs, were distributed within 2 kb from the TSS, peaking around -700 nt (Fig 4B). A similar peak was found in the sense direction with a similar mode of distribution (Fig 4B). For simplicity, we called transcripts using pAs within 2 kb from the TSS in the sense direction (excluding those in 3’-most exons or in single exon genes) sense proximal RNAs (spRNAs).

Nucleotide frequency analysis of the regions around pAs of uaRNAs and spRNAs indicated that they each had distinct nucleotide profiles compared to those of 3’-most pAs (Fig 4C). While similar U-rich and A-rich upstream peaks and a U-rich downstream peak were found for all types of pAs, the upstream regions of both uaRNA and spRNA pAs had a higher GC content, in line with their location close to promoters with CpG islands [46]. In addition, compared to uaRNA and 3’-most exon pAs, spRNA pAs had a lower A-content in surrounding regions (Fig 4C).

We next examined our KD samples with the goal of finding factors involved in expression of uaRNAs or spRNAs. Using isoforms whose pAs were beyond 2 kb from the TSS as a reference, we examined uaRNA and spRNA regulations in the KD samples by GAAP (Fig 4D and 4E). Significant upregulations of both uaRNA and spRNA were observed with the siPABPN1 sample, with upregulation of uaRNAs being greater than that of spRNAs (Fig 4D and 4E). By contrast, consistent with the findings of Almada et al. [37], regulation by U1 inhibition, such as U1D (8 hr), U1D (24 hr) or siU1-70K, showed the opposite trend, with spRNAs being more significantly upregulated than uaRNAs (Fig 4D and 4E). The siSF3b155 sample also showed significant regulation of uaRNAs, but the number of upregulated events was similar to that of downregulated ones (Fig 4D). In contrast, spRNAs were generally upregulated by siSF3b155 (Fig 4E). The difference between siPABPN1 and U1 inhibition in regulating spRNAs and uaRNAs can also be seen with the isoform expression profiles around the TSS (Fig 4F and 4G).

While upregulation of spRNAs by U1 inhibition may be related to the role of U1 in suppressing C/P near the TSS [37], the regulation of spRNAs and uaRNAs by PABPN1 is completely not clear. Studies have shown that the nuclear exosome is involved in degrading uaRNAs [38] and PABPN1 is involved in stability of nuclear RNAs [47, 48]. We thus asked whether the regulation of uaRNA and spRNA expression by PABPN1 was related to exosome-mediated RNA decay. To this end, we knocked down Rrp44 and Rrp6 together by siRNAs, two nucleases associated with the exosome, followed by 3’READS. As expected, their KD led to higher abundance of uaRNAs and spRNAs, presumably through stabilization of these transcripts (Fig 4H). Importantly, the uaRNA and spRNA profiles in the siRrp44 + siRrp6 sample resembled those of siPABPN1, suggesting a functional connection between PABPN1 and the exosome in regulating uaRNAs and spRNAs.

### Detailed study of five C/P factors

**Fig 5:**
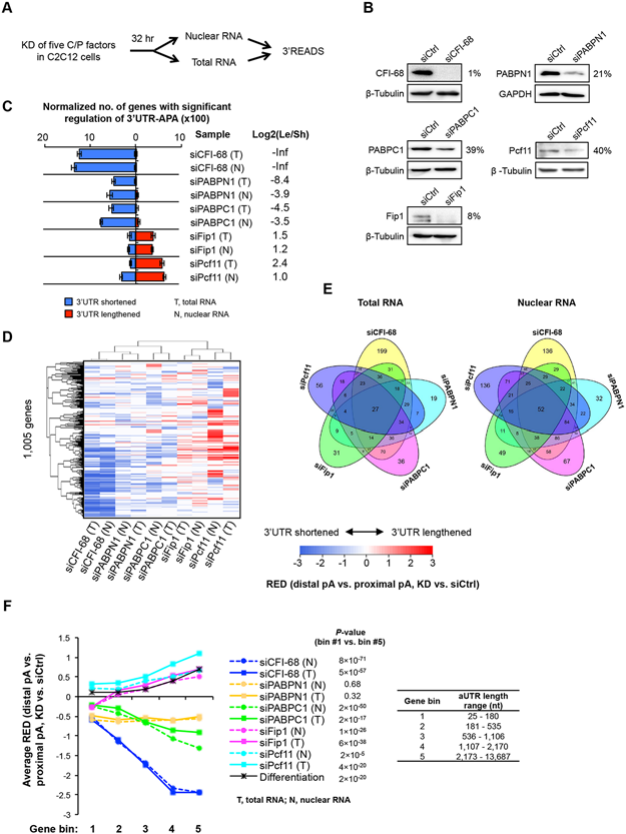
Detailed analysis of five C/P factors. **(A)** Schematic of the experimental design. Proliferating C2C12 cells were harvested 32 hr after knockdown (KD), and both total and nuclear RNAs were extracted for 3’READS analysis. **(B)** Western Blot analysis of protein expression after 32 hr of KD. The percent of expression in KD cells compared to siCtrl cells for each KD is indicated. **(C)** Normalized number of genes with regulated 3’UTR-APA in each sample as examined by GAAP. See Fig 2D for details of the plot. Both total and nuclear RNA data are shown. **(D)** Cluster analysis of 3’UTR-APA regulation by the five factors. RED scores using the top two most abundant APA isoforms (based on the number of poly(A) site-supporting reads in all samples) of each gene were used for this analysis. Only pA isoforms with read number ≥5 in all samples were used. A RED score is difference in relative expression of distal pA isoform vs. proximal pA isoform between KD and siCtrl cells, as illustrated in Fig 2A. RED scores are represented in a heatmap using the color scheme shown in the graph. Positive and negative RED scores indicate lengthened and shortened 3’UTRs, respectively. RED scores for APA events were set to 0 when q-value > 0.05 (SAAP). Pearson correlation was used as metric for hierarchical clustering. **(E)** Venn diagrams comparing genes with significant 3’UTR-APA regulations by the five factors using total RNA (left) or nuclear RNA (right) data. **(F)** Relationship between extent of 3’UTR-APA regulation and aUTR size. Genes were divided into five bins based on the aUTR size (distance between the pAs of top two most abundant APA isoforms). The aUTR size range for each bin is shown in the graph. The extent of 3’UTR-APA regulation is represented by average RED scores, based on the data shown in (D). Only genes with ≥20 PASS reads (proximal and distal pAs combined) in both KD and siCtrl samples were used for RED calculation. RED scores of genes in bin #1 were compared with those in bin #5 by the Wilcoxon rank sum test for each sample, and p-values are shown.

Since 3’UTR-APA had much greater regulations than CDS-APA or expression of uaRNAs or spRNAs, as indicated by the number of genes with significant APA changes in KD samples (GAAP analysis results in Figs 2, 3 and 4), we wanted to further examine how different factors regulate 3’UTR-APA. While our GAAP analysis based on random sampling of genes enabled comparison of global APA patterns across samples, this approach is not suitable for comparing individual pA usage across samples due to the batch effect. We therefore repeated KDs of several key C/P factors in one batch, which showed substantial APA regulations in our initial study, including CFI-68, PABPN1, PABPC1, Fip1 and Pcf11. We did not include CFI-25 because the APA profile of its KD was highly similar to that of siCFI-68 (S6 Fig). To mitigate indirect effects and the influence of mRNA decay in cytoplasm, we harvested cells 32 hr after siRNA transfection and extracted both total and nuclear RNAs for 3’READS analysis (Fig 5A). Western Blot analysis indicated that the KD efficiencies in these samples were >60% at the time of cell harvest (Fig 5B).

We found that using total or nuclear RNAs from 32 hr KD samples gave rise to similar results to using total RNAs from 48 hr KD samples (Fig 5C), with siCFI-68, siPABPN1, and siPABPC1 leading to 3’UTR shortening and siPcf11 and siFip1 causing 3’UTR lengthening. This result indicates that our initial observations were not obstructed, at least not substantially, by indirect effects (which could be introduced over time), or by mRNA decay in cytoplasm (which could lead to different APA profiles between nuclear and total RNAs). This notion was further confirmed by detailed comparisons of total RNAs from 32 hr vs. 48 hr KD samples (S7 Fig) and total vs. nuclear RNAs from 32 hr KD samples (S8 Fig), which showed significant correlations of APA changes of genes. Moreover, gene expression changes were largely uncoupled from 3’UTR changes using both nuclear or total RNAs (S9 Fig), indicating that APA isoforms are not likely to differ in mRNA stability in general. However, a mild difference in gene expression could be discerned between genes with shortened and lengthened 3’UTRs in the siPcf11 sample (S9 Fig), suggesting a potential role of Pcf11 in post-transcriptional regulation of gene expression. This will be explored in the future.

Because the five factor KD samples were processed in the same batch, we directly compared their APA profiles. Using RED scores based on the top two most abundant 3’UTR isoforms of each gene, we performed cluster analysis (Fig 5D). We found that total and nuclear RNA data were clustered together for all KD samples, consistent with the notion that cytoplasmic mRNA decay did not substantially alter APA profiles in our samples. In addition, samples involving global 3’UTR shortening, i.e., siCFI-68, siPABPN1 and siPABPC1 samples, were separated from those involving global lengthening, i.e., siPcf11 and siFip1 samples, suggesting that some common sets of pAs were regulated by different factors. On the other hand, Venn diagram analysis of APA events regulated by the five factors also indicated that a fraction of pAs were distinctly regulated by these five factors (Fig 5E): Each factor regulated a set of unique APA events and the number of APA events regulated by all five factors was quite small (27 using total RNA data, and 52 using nuclear RNA data, Fig 5E), indicating distinct regulatory mechanisms among the factors.

Because of the relevance of aUTR length to APA regulation (Fig 2F), we next examined the extent of APA regulation for genes with different aUTR lengths (Fig 5F). Genes were first divided into five equally sized bins based on their aUTR length (Fig 5F). The extent of APA regulation was represented by the RED score. We found that genes that had longer aUTRs tended to have greater APA regulation than genes with shorter ones in all samples except siPABPN1. This is supported by 1) the trend of RED score across gene bins and 2) the p-values indicating the difference in RED scores between the first and fifth gene bins (with the shortest and longest aUTRs, respectively)(Fig 5F). Intriguingly, this analysis also revealed that in the siFip1 sample, genes with the shortest aUTRs (bin 1) showed 3’UTR shortening in general whereas genes in other bins showed the opposite trend (Fig 5F). This dichotomous APA pattern with respect to aUTR size was not seen with other samples, indicating a unique aUTR size-dependent mechanism of Fip1 in 3’UTR-APA regulation (see below for more analyses).

### Cis elements in APA regulation by the five C/P factors

**Fig 6:**
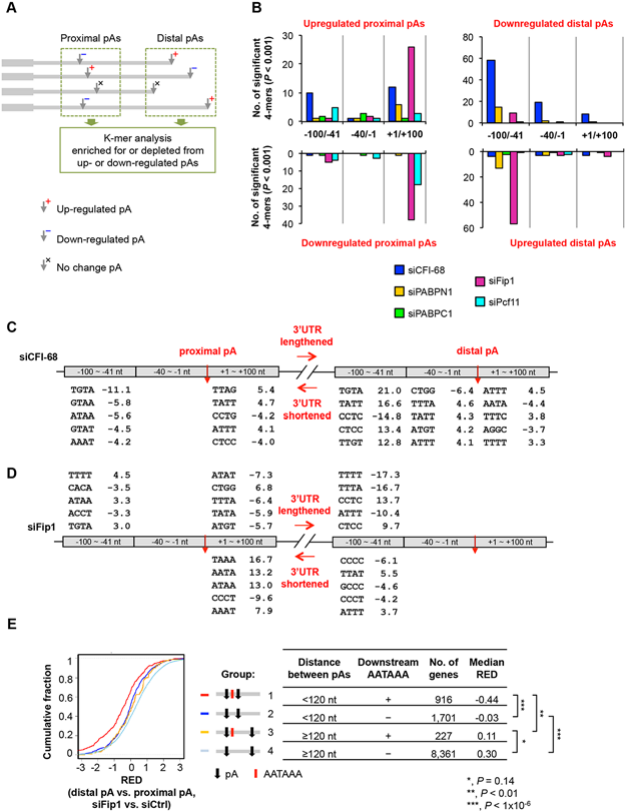
Cis elements associated with regulated pAs. **(A)** Schematic showing the analysis method. As indicated, pAs were divided into proximal and distal pA groups, and pAs of regulated isoforms were compared only with other pAs in the same group. As such, proximal pAs were only compared with proximal pAs and so were distal pAs. pAs of upregulated and downregulated isoforms were analyzed separately. Only data of nuclear RNA samples were used for analysis, because they were expected to have less post-transcriptional effects than total RNA samples. **(B)** Number of 4-mers with significantly biased frequency of occurrence (*P* < 0.001, Fisher’s exact test) near regulated pAs. Regulated pAs were those with q-value < 0.05 (SAAP). Three regions around the pA were analyzed, including -100 to -41 nt, -40 to -1 nt and +1 to +100 nt. Data for all 4-mers and top 6-mers are shown in S5 Table and S6 Table, respectively. **(C)** Significant 4-mers enriched for or depleted from pAs regulated by siCFI-68. Only top five 4-mers for the regions with ≥5 significant 4-mers are shown. Numbers are significance score (SS), which was calculated by –log_10_(*P*)*S, where *P* was based on the Fisher’s exact test and S = 1 for enrichment and -1 for depletion. **(D)** As in (C), significant 4-mers enriched for or depleted from pAs regulated by siFip1. **(E)** Regulation of different types of pAs by siFip1, as shown by Cumulative Distribution Function (CDF) curves of RED scores. Genes were divided into four groups based on i) distance between proximal and distal pAs (<120 nt or ≥120 nt), and 2) whether or not there was AAUAAA within 100 nt downstream of the proximal pA. These groups are also illustrated in the graph. The number of genes and the median RED score of each group are shown in a table. The differences between groups are indicated by p-values (Kolmogorov–Smirnov test).

We next asked whether cis elements around the pA contributed to differential APA regulation in different KD samples. Since proximal and distal pAs tend to be surrounded by different cis elements [49], we analyzed these two pA groups separately (illustrated in Fig 6A). For each group, we compared pAs that were regulated in one of the five KD samples with pAs regulated in other samples. As such, the identified cis elements should be specific for the factor under investigation, and should not be caused by pA relative locations. We examined three sub-regions around the pA, namely, -100 to -41 nt, -40 to -1 nt and +1 to +100 nt (the pA was set to position 0), for significantly enriched and depleted K-mers (4-mer or 6-mer, *P* < 0.001, Fisher’s exact test). Fig 6B shows the number of significant 4-mers for each region, reflecting the extent to which cis elements in the region were involved in pA regulation.

We found much greater 4-mer biases around pAs regulated by siCFI-68 or siFip1 than those by siPcf11, siPABPC1 or siPABPN1 (Fig 6B and S5 Table). In the siCFI-68 sample, major biases were found in regions surrounding both proximal and distal pAs of genes with shortened 3’UTRs (Fig 6B and 6C), including depletion of TGTA in the -100 to -41 nt region of proximal pAs (significance score (SS) = -11.1; SS = -log10(p-value)*S, where p-value was based on the Fisher’s exact test and S was 1 for enrichment or -1 for depletion) and enrichment of this motif in the same region of distal pAs (SS = 21.0), suggesting that the presence or absence of TGTA in the -100 to -41 nt region is a major reason for APA regulation by CFI-68. This result is consistent with the binding site for CFI complex [24, 43, 50]. Other motifs were also found to be significantly biased in these regions, as well as in the +1 to +100 nt region of proximal pAs, and the -40 to -1 nt and +1 to +100 nt regions of distal pAs, albeit with less significance (Fig 6C).

In the siFip1 sample, both pAs of shortened 3’UTRs and of lengthened 3’UTRs displayed motif biases (Fig 6D). For lengthened 3’UTRs, there was an enrichment of TTTT in the -100 to -41 nt region of proximal pAs, and an depletion of the motif in the same region of distal pAs, suggesting that U-rich elements play a role in APA regulation mediated by Fip1. This result is consistent with the reported U-rich sequence binding for Fip1 [51] and is in good agreement with the binding locations reported by Lackford et al. [31]. Several 4-mers displayed a strong bias in the +1 to +100 nt region of proximal pAs of shortened 3’UTRs, including TAAA, AATA and ATAA. Hexamer analysis indicated that these motifs were derived from AATAAA (S6 Table). This result suggests that Fip1 inhibits usage of pAs with downstream AATAAA. To test this hypothesis further, we specifically examined pAs with or without downstream AATAAA and analyzed the influence of distance between pAs (Fig 6E). We found that, in the siFip1 sample, when the distance between proximal and distal pAs was < 120 nt, proximal pAs with downstream AATAAA within 100 nt (group 1, Fig 6E) tended to be much more used than those without the motif (group 2), as indicated by their RED scores (-0.44 vs. -0.03, *P* < 1x10^-6^, Kolmogorov–Smirnov, or K-S, test,). This trend could also been seen when the distance was > = 120 nt (0.11 vs. 0.30, group 3 vs. group 4, *P* = 0.14). Thus, Fip1 plays a role in selection of adjacent pAs, favoring AATAAA-associated downstream pAs. This result offers an explanation as to why genes with short aUTRs tended to have upregulated proximal pAs in the siFip1 sample (Fig 5F).

Some cis element biases were also found in siPcf11 and siPABPN1 samples, with lower statistical significance (Fig 6B, S5 Table, and S6 Table). For example, TATT and TTAT were enriched for the +1 to +100 nt region of proximal pAs downregulated by siPcf11, and several TA-rich motifs were depleted from the -100 to -41 nt region of downregulated distal pAs but enriched for the same region of upregulated distal pAs in the siPABPN1 sample. Of all samples, siPABPC1 displayed the least cis element bias around regulated pAs, suggesting that its regulation of C/P does not involve specific cis elements.

### Relevance of the APA regulation by the five C/P factors to cell differentiation and development

**Fig 7:**
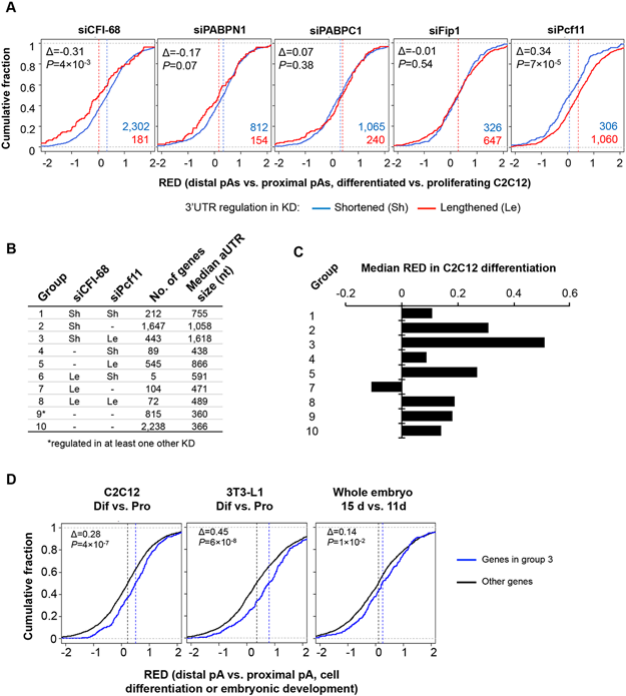
Comparison of APA events regulated by the five C/P factors with those regulated in C2C12 differentiation. **(A)** APA regulation in C2C12 differentiation for genes that showed shortened 3’UTRs (Sh, blue line) or lengthened 3’UTRs (Le, red line) in different knockdown (KD) samples (total RNA only). APA regulation in C2C12 differentiation is represented by Cumulative Distribution Function (CDF) curves of RED scores. Only genes with q-value < 0.1 (SAAP) were used. The numbers of Sh genes and Le genes are shown in blue or red, respectively. The Δ value is difference between the median RED scores of Le and Sh genes. Median RED scores are also indicated by vertical dotted lines. P-values (Kolmogorov–Smirnov test) are shown to indicate RED score difference between Le and Sh genes in C2C12 differentiation. **(B)** Ten groups of genes based on 3’UTR-APA regulation by siCFI-68 and siPcf11 (q-value < 0.1). Group 9 contained genes whose 3’UTRs were regulated by siPABPN1, siPABPC1, or siFip1. Group 10 contains other genes whose 3’UTR-APA isoforms were detectable in C2C12 cells. Le, 3’UTR lengthened; Sh, 3’UTR shortened. Number of genes and median aUTR size are also shown. **(C)** Median RED scores for gene groups shown in (B) in C2C12 differentiation. Group 6 is not included because of a very small number of genes (5) in the group. **(D)** RED scores of group 3 genes were compared with those of genes in other groups in C2C12 differentiation (left), 3T3-L1 differentiation (middle), and embryonic development (15 day vs. 11 day, right). As in (A), difference between the median RED scores of two sets (Δ) and p-value (Kolmogorov–Smirnov test) comparing the two sets are also shown.

We next asked whether the regulated APA events by five C/P factors were related to those taking place during C2C12 differentiation [14]. We examined 3’UTR-APA in C2C12 differentiation for genes that showed shortened or lengthened 3’UTRs in different KD samples (32 hr KD, total RNA). Using RED scores for differentiated vs. proliferating C2C12 samples, we found that genes regulated by siCFI-68 and siPcf11 showed significant RED differences in C2C12 differentiation (Fig 7A): genes with shortened 3’UTRs in the siCFI-68 sample and those with lengthened 3’UTRs in the siPcf11 sample were more likely to have lengthened 3’UTRs in C2C12 differentiation than genes with the opposite 3’UTR regulation in their respective samples (*P* = 4x10^-3^ and 7x10^-5^, respectively, K-S test).

Based on APA regulations by siCFI-68 and siPcf11, we next divided genes into 10 groups (Fig 7B and S7 Table) and asked how different sets of genes were regulated in C2C12 differentiation. Genes in group 3, whose 3’UTRs were shortened by siCFI-68 and lengthened by siPcf11 showed greatest 3’UTR lengthening in C2C12 differentiation as indicated by their RED median (Fig 7C). They were followed by group 2 genes, whose 3’UTRs were shortened by siCFI-68 but not regulated by siPcf11, and group 5 genes, whose 3’UTRs were lengthened by siPcf11 but not by regulated by siCFI-68 (Fig 7C). Interestingly, genes in groups 3, 2 and 5 had longer aUTRs than genes in other groups, with median size of 1,618, 1,058 and 866 nt, respectively (Fig 7B), highlighting the importance of distance between pAs for APA regulation in C2C12 differentiation. Further analysis using our previous 3’READS data from 3T3-L1 pre-adipocyte differentiation and embryonic development (15 day vs. 11 day)[10] indicated that group 3 genes also tended to have significantly greater 3’UTR lengthening than other genes in these processes (*P* = 6x10^-8^ and 1x10^-2^, K-S test comparing group 3 genes with all other genes, Fig 7D), similar to their regulation in C2C12 differentiation (*P* = 4x10^-7^, Fig 7D). Interestingly, group 3 genes were significantly associated with several Gene Ontology (GO) terms (S10 Fig), such as “single-organism membrane organization”, “spermatogenesis”, “actomyosin structure organization”, “intracellular protein transport”, “cell body” and “cytoplasmic vesicle.” Notably, several different GOs were also found to be associated with group 2 genes, such as “endosome to lysosome transport”, “transforming growth factor beta receptor signaling pathway”, “cellular response to endogenous stimulus”, “cell leading edge”, “coated vesicle membrane”, “cell junction”, and “endomembrane system.” Intriguingly, group 1 genes, whose 3’UTRs were shortened by both siCFI-68 and siPcf11, also showed strong association with several GO terms, such as “regulation of phosphorylation”, “cell death”, “intracellular signal transduction”, etc. Taken together, these data indicate that genes in different functional groups may be differentially regulated by APA in cell differentiation due to distinct pA and aUTR features.

## Discussion

Here we present a systematic study examining modulation of several types of APA by all known core C/P factors and several key splicing factors. Using proliferating C2C12 cells, we were able to compare the impacts of different factors on APA in a single system, and correlate the regulations with those taking place during C2C12 differentiation. We also developed a statistical method SAAP to assess the significance of APA regulation and employed a computational approach GAAP to address batch effects and normalize sequencing depth. Comparisons between KD samples at different time points and between total and nuclear RNA samples indicated that our results were not substantially affected by indirect effects. However, off-target effects by siRNAs are possible, despite the use of multiple siRNAs in most samples. In addition, different KD efficiencies by different siRNAs can lead to variable effects on APA, making it difficult to compare KD samples directly. However, we did not find a correlation between the level of KD and the extent of 3’UTR-APA regulation (S11 Fig), suggesting our observations were not severely affected by variable KD efficiencies. Nevertheless, future work using more specific KD or knockout methods may be desirable for more precise comparisons.

**Fig 8:**
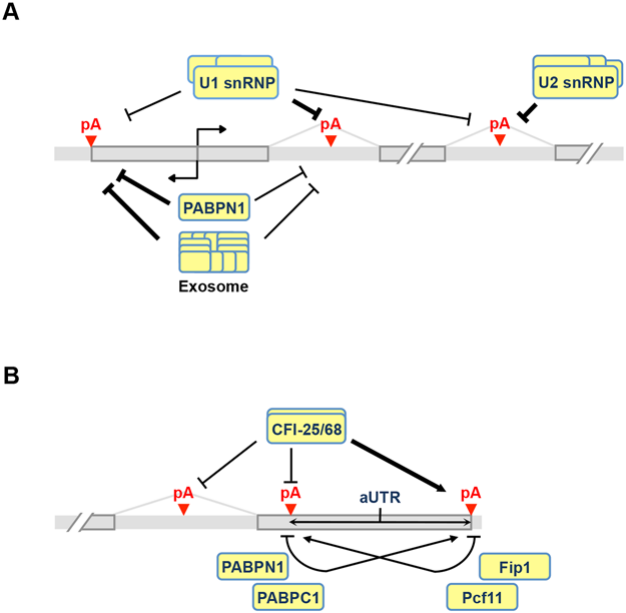
APA models. **(A)** Regulation of C/P events near the transcription start site (TSS) and introns. Both U1 and PABPN1 inhibit polyA(+) transcript expression near the TSS. For U1, inhibition of sense strand RNA is more prominent than that of upstream antisense RNA (uaRNA) due to higher frequency of its binding sites, i.e., 5’ splice site or related sequences, in the sense strand. The mechanism is likely to be inhibition of polyadenylation. PABPN1 has the opposite trend, with suppression of uaRNA expression being more significant. This function of PABPN1 is likely to involve exosome-mediated RNA decay. U1 and U2 inhibit intronic C/P because C/P is in a kinetic competition with splicing. **(B)** Regulation of pA usage in the last intron and the 3’-most exon. CFI-25/68 promotes usage of distal pAs through binding to the UGUA element, leading to longer 3’UTRs and selection of downstream terminal exons. PABPN1 and PABPC1 also promote distal pA usage, whereas Pcf11 and Fip1 promote proximal pA usage. Note that if two pAs are close to each other and there is an AAUAAA motif downstream of the proximal pA, Fip1 helps select the distal pA (not indicated in the graph). aUTR size is indicated to highlight its importance in APA regulation.

Notwithstanding the potential technical limitations, we were able to make a number of key findings concerning the contributions of specific C/P and splicing factors to APA, and to address how they pertain to APA regulation in cell proliferation/differentiation. Below we discuss these findings; some of the conclusions are illustrated in Fig 8.

### CPSF

Among the CPSF subunits, Fip1 levels were found to have the greatest impact on APA, suggesting that Fip1 recruitment is a key step for CPSF’s function in C/P, at least in the context of C2C12 cells. Given the essential role of CPSF in the C/P reaction, it is not surprising that inhibition of Fip1 leads to 3’UTR lengthening: downregulated C/P activity would favor distal pAs, which in general have stronger C/P cis elements than proximal ones [6]. Interestingly, a group of genes had 3’UTR shortening after Fip1 KD; their proximal pAs tend to have the AAUAAA motif in the downstream region and the distance between proximal and distal pAs is short. This result is largely in line with what was observed by the Shi group with Fip1 KD in embryonic stem cells [31]. As proposed by Lackford et al., there exist two modes of APA regulation by Fip1 depending upon pA strength and distance between pAs [31]. We additionally found that while selection of distal pAs in the siFip1 sample involves U-rich elements, activation of proximal pAs does not, suggesting that U-rich element binding by Fip1 plays a role in pA selection only when competing pAs are far apart. Recent studies have revealed that CPSF30 and WDR300 directly bind PAS, [52, 53]. Intriguingly, KD of any one of these factors had a modest effect on APA. Whether these factors can compensate each other in PAS interaction is to be examined in the future.

### CFI

Consistent with the findings by the Zavolan and Keller groups [24, 43], KD of CFI-25 or CFI-68, but not CFI-59, led to a substantial shift to proximal pA usage in 3’UTRs, the extent of which is more significant than other samples examined in this study. The CFI complex, composed of either CFI-25/CFI-68 or CFI-25/CFI-59, has been shown to interact with UGUA elements [50], which are typically located upstream of the PAS [49]. Consistently, distal pAs downregulated by siCFI-25/siCFI-68 were enriched with UGUA element(s) in the -100 to -41 nt region whereas upregulated proximal pAs were depleted of them in the same region. Some other cis elements were also identified around regulated pAs (Fig 6C), such as UAUU, CCUC. Whether they simply piggyback with UGUA or are actively engaged in binding with some proteins that interact with CFI need to be further studied in the future.

We also found that CFI-25 or CFI-68 KD led to general upregulation of isoforms using pAs in the last intron, a feature that has not been reported in previous studies [24, 43], suggesting that CFI-25/68 also play a role in 3’ terminal exon selection. One possibility is that removal of the last intron is slow, creating a time window in which pAs in the last intron compete with 3’-most exon pAs for C/P. It is also possible that CFI-25/CFI-68 may facilitate the removal of the last intron, e.g., through interaction with splicing factors [33], thereby inhibiting pA usage in the last intron.

### CFII

Our data indicate that Pcf11 has a substantial impact on APA, which has not been detected previously in metazoan cells. In *S*. *cerevisiae*, deletion of the mRNA export adaptor Yra1, which inhibits C/P through competition with Clp1 for Pcf11 binding, leads to widespread APA events [54]. The same study also suggested that Clp1 and Pcf11 are not necessarily recruited as a complete unit in yeast. Our data supports this view, because siPcf11 and siClp1 had different effects on APA, with respect to extent and direction of regulation. This notion is also in line with the report by Shi et al. showing that Clp1 was not present in the C/P complex purified from mammalian cells [22]. Given the significant role of Pcf11 in APA, it is possible that its recruitment to the C/P complex is a rate-limiting step for the C/P reaction.

### PABPs

Inhibition of PABPN1 has been shown to elicit global shortening of 3’UTRs via APA [27, 28]. It was suggested that this regulation may be relevant to the etiology of human disease oculopharyngeal muscular dystrophy (OPMD), which is caused by an expansion mutation in the polyalanine repeat in the N-terminus of PABPN1. Our results are largely in line with these findings but also revealed that modulation of 3’UTR-APA by PABPN1 is quite different than other C/P factors. For example, there is no relationship between the extent of 3’UTR-APA regulation (based on number of genes with APA changes) and 3’UTR size changes. We also found that PABPN1 plays a very significant role in inhibition of uaRNA and spRNA expression (with regulation of uaRNAs being more significant). This property may be related to PABPN1’s role in RNA stability [47, 48]. Consistently, the uaRNA and spRNA profiles in the siPABPN1 sample are similar to those in the siRrp44 + siRrp6 sample. A key question thus is whether the 3’UTR-APA regulation by PABPN1 is related to its functions around the TSS. We did not find significant 3’UTR-APA in the siRrp44/siRrp6 sample (S12 Fig) and genes with 3’UTR-APA regulation by siPABPN1 did not appear to have significant expression changes overall (S9 Fig), suggesting that 3’UTR-APA regulation by PABPN1 is not related to its function in uaRNA and spRNA regulation. How PABPN1 exerts distinct functions at the 5’ and 3’ ends of genes respectively awaits further experimentation.

Interestingly, PABPC1, which binds the poly(A) tail in the nucleus [55] and shuttles between nucleus and cytoplasm [56], appears to be as potent as PABPN1 in regulation of 3’UTR-APA, raising the possibility that PABPs in general can regulate the C/P reaction. However, it is also notable that the APA events regulated by PABPC1 are quite different than those by PABPN1, with respect to uaRNA and spRNA expression changes and the role of aUTR size in regulation. There are no obvious cis elements enriched for pAs regulated by PABPC1 but aUTR size appears to be related to its APA regulation, suggesting that PABPC1 may have a general impact on APA without sequence specificity. Future work will be needed to delineate the respective roles of these PABPs as well as other poly(A)-binding proteins [57] in APA.

### CstF and other C/P factors

CstF subunits appear to have modest impacts on APA in this study. While siCstF-50 and siCstF-77 led to global 3’UTR lengthening, consistent with their roles in C/P, siCstF-64 did not result in a directional change of APA and siCstF-64τ in fact led to mild general 3’UTR shortening. These puzzling results may be due to the fact that CstF-64 and CstF-64τ have overlapping functions [29] and can regulate each other’s expression [58]. As such, KD of one factor to different levels may give rise to different overall activities, leading to complex results. Thus, the single factor KD-based approach used in this study would be unwieldy in elucidating the exact roles of CstF-64 and CstF-64τ in APA. This issue may also apply to other factors which showed modest APA regulation in this study. For example, there are several PAPs in the cell and KD of one PAP, such as PAPα or PAPγ, may be compensated functionally by other PAPs.

### Splicing in APA

Splicing is believed to be intimately involved in C/P (reviewed in [33]). Our data define two general modes of APA regulation involving splicing. First, U1 plays a significant role in suppressing C/P in 5’ end introns, which is consistent with the findings made by the Dreyfuss group [35] and is in line with the inhibitory activity of U1 on C/P [36]. In agreement with the telescripting model proposed by the Dreyfuss group [39], inhibition of intronic pA usage by U1 displays 5’ to 3’ polarity (Fig 3C). But we only observed mild 3’UTR lengthening in U1 inhibition samples, suggesting a modest role of telescripting in 3’UTR-APA. However, we cannot rule out the possibility that difference in cell type and experimental conditions may lead to this discrepancy. Second, as supported by the siSF3b155 data, splicing activity in general plays a role in suppressing intronic C/P (Fig 3B). It remains to be seen how other splicing factors, particularly those involved in alternative splicing, globally regulate intronic APA and impact selection of 3’ terminal exons.

### APA code in proliferation/differentiation

Our data suggest that an APA event in a given cellular condition is regulated by a number of parameters, including relative location to the TSS, splicing context, distance between competing pAs, surrounding cis elements, and concentrations of C/P factors. In the context of C2C12 differentiation, which involves global 3’UTR lengthening, almost all C/P factors showed downregulation, at least at the mRNA level (S13 Fig). However, given the diverse consequences of different C/P factor KDs, it would be too simplistic to attribute the 3’UTR lengthening to downregulation of C/P factors as a whole. On the other hand, several lines of evidence support similarities in 3’UTR-APA regulation between C/P factor KD and C2C12 differentiation. First, aUTR size appears to be an important factor in APA regulation in both KD cells (with the exception of siPABPN1) and cell differentiation. In general, a longer aUTR confers more regulability. Since longer aUTRs could contain more cis regulatory elements for mRNA metabolism, this result suggests that genes with highly regulatable APA tend to be more controlled post-transcriptionally as well. Second, groups of genes with significant regulation by siCFI-68 and siPcf11 are also substantially regulated in differentiation, implying similar mechanisms in these KD conditions and cell differentiation. Importantly, these genes also displayed significant 3’UTR-APA in differentiation of 3T3-L1 cells and embryonic development, suggesting a general APA code in cell proliferation/differentiation. Future studies need to test the APA code with more perturbations, such as overexpression of different factors, and to explore the input of different RNA-binding proteins in condition- and tissue-specific APA regulations [59].

## Materials and Methods

### Cell culture, transfection, RT-qPCR and western blot

C2C12 cells were maintained in Dulbecco's Modified Eagles Medium (DMEM) supplemented with 10% fetal bovine serum (FBS). Differentiation of C2C12 cells was induced by switching cell media to DMEM+ 2% horse serum (Sigma) when cells were ~90–100% confluent. All media were also supplemented with 100 units/ml penicillin and 100 μg/ml streptomycin. Differentiated C2C12 cells in this study were harvested four days after differentiation. Transfection with siRNAs was carried out by Lipofectamine 2000 (Invitrogen) according to manufacturer’s recommendations. Transfection was carried out for 48 hr or 32 hr. siRNA sequences are shown in S1 Table. The U1D oligo (5'-gCcAgGuAaGuau) and mutant U1D (mU1D) oligo (5'-gCcAgGcAcGuau), where locked nucleic acid (LNA) residues are in uppercase and 2’-OMe RNA bases in lowercase, were previously described in [42]. These oligos were transfected into C2C12 cells at 35 nM using Lipofectamine 2000 when the confluency of cells was about 50%. Cells were harvested 8 hr or 24 hr after transfection. For nuclear RNA extraction, cells collected by a scrapper were suspended in cell lysis buffer (10 mM Tris pH 7.4, 10 mM NaCl, 0.5% NP40, 1 mM DTT), followed by vortexing for 10 sec and incubation on ice for 10 min. After centrifugation of the lysate at 500 x g for 5 min at 4°C, the pellet was re-suspended in the cell lysis buffer for nuclear RNA extraction. Both total and nuclear RNAs were extracted using Trizol (Invitrogen) according to manufacturer's protocol. RNA quality was examined in an Agilent Bioanalyzer using the RNA pico600 kit before processing for deep sequencing. For RT-qPCR, mRNA was reverse-transcribed using an oligo(dT) primer, and qPCR was carried out with Syber-Green I as dye. Primer sequences are listed in S3 Table. Antibodies used for Western Blot analysis are listed in S2 Table.

### 3’READS

The 3’READS method used in this study was previously described [60]. Briefly, 25 μg of input RNA was used for each sample, and poly(A)+ RNA was selected using oligo d(T)25 magnetic beads (NEB), followed by on-bead fragmentation using RNase III (NEB). Poly(A)+ RNA fragments were then selected using the chimeric U_5_ and T_45_ (CU_5_T_45_) oligo conjugated on streptavidin beads, followed by RNase H (NEB) digestion. Eluted RNA fragments were ligated with 5’ and 3’ adapters, followed by RT and PCR (15x) to obtain cDNA libraries for sequencing on the Illumina platform. All data can be obtained from the NCBI GEO database (GSE62001). Processing of 3’READS data was carried out as previously described (Hoque et al. 2013). Briefly, reads were mapped to the mouse genome using bowtie 2 (Langmead and Salzberg 2012). Reads with ≥2 unaligned Ts at the 5’ end are called poly(A) site-supporting (PASS) reads, which were used to identify pAs. pAs located within 24 nt from each other were clustered together. The number of PASS reads generated in each sample is listed in S4 Table.

### Significance analysis of alternative polyadenylation

We developed a randomization-based method to statistically assess the significance of difference between two samples for each APA event, called Significance Analysis of Alternative Polyadenylation, or SAAP. The method is illustrated in S2 Fig. Briefly, for two pAs (or two pA sets) from two comparing samples, a Relative Expression Difference (RED) score is first calculated (S2 Fig). The PASS reads are then sampled based on the assumption that the relative abundance of each pA isoform is the same in two samples. Sampling is preformed *m* times (20 in this study) to obtain mean and standard deviation of RED. The observed and expected RED values are standardized to Z scores (minus mean and divided by standard deviation). False Discovery Rate (FDR) and q-value are calculated by comparing observed Z (Zo) and expected Z (Ze) for a given Z cutoff value (Zc).

For 3’UTR-APA, we either selected the two most abundant pA isoforms for analysis, or used all pA isoforms for analysis (one vs. others) and then selected the top two most regulated isoforms. For CDS-APA, we combined all isoforms using pAs in upstream regions of the 3’-most exon and compared their expression change with that of isoforms using pAs in the 3’-most exon. Individual intronic pAs were also analyzed by comparing to all other pA isoforms of the gene. For uaRNA analysis, we combined all antisense transcripts using pAs within 2 kb upstream of the transcription start site (TSS), excluding those mapped to other mRNA genes, and compared them to all sense strand transcripts, excluding pAs located within 2 kb downstream of the TSS. For spRNA analysis, we grouped pAs located within 2 kb downstream of the TSS, excluding those located in 3’-most exons or in single exon genes, and compared them with other sense strand isoforms. We used q-value < 0.05 (SAAP) to select significantly regulated APA events.

### Global analysis of alternative polyadenylation

We developed an approach named Global Analysis of Alternative Polyadenylation (GAAP) to normalize sequencing depth and to obtain expected values for a given data set. The method is illustrated in S2 Fig. Let A and B be two 3’READS data sets for two samples that are processed at the same time. For example, A is a KD sample and B is a control sample. Let a and b be two data sets sampled by bootstrapping from A and B, respectively. Bootstrapping is carried out *n* times with *p* number of PASS reads sampled each time. In this study, *n* = 20, and *p* = 1.5 M. Let A’ and B’ be randomly permutated data sets based on A and B. In permutation, PASS reads from A and B are first combined and then sampled without replacement to obtain A’ and B’, with the total number of PASS reads of the permutated sets A’ and B’ being the same as those of the original sets A and B, respectively. Permutation is carried out *m* times. Two sets after each permutation (a’ and b’) are sampled with *q* number of reads from each set by bootstrapping. In this study, *m* = *n*, and *q* = *p*. The permutated sets provide expected values (Exp), whereas the original set provides observed values (Obs). APA analyses of a vs. b and a’ vs. b’ were carried out by SAAP.

### K-mer analysis

To identify cis elements biased to or against pAs regulated in a KD sample, we compared k-mer frequencies around the pAs regulated in the sample with other pAs regulated in other samples. To mitigate the possibility that identified cis elements are related to location, we first grouped all proximal and distal pAs together into two separate sets (illustrated in Fig 6A). For each KD sample, regulated proximal pAs were compared to other proximal pAs to identify overrepresented and underrepresented k-mers. The same approach was used for distal pAs. We examined three regions around the pA, i.e., -100 to -41 nt, -40 to -1 nt and +1 to +100nt. For each region, the Fisher’s exact test was used to examine whether a k-mer (4-mer or 6-mer) was enriched for or depleted from a set of pAs vs. other pAs.

### Analysis of introns

The intron location was based on the RefSeq database, with all RefSeq splicing isoforms combined. Distribution of regulated intronic pA isoforms was compared to that of background set, which was derived from all detected intronic pAs in all control samples in this study (isoform relative abundance ≥5% in at least two samples and read count ≥2 in at least two samples). To calculate 5’SS or 3’SS strength, we used all 5’SS or 3’SS supported by mouse RefSeq sequences. The maximum entropy scores were calculated by MaxEntScan [61]. The 5’SS or 3’SS strength of introns containing regulated pAs was compared to that of background introns with the same relative location in the gene by the Wilcoxon rank sum test.

### Other analyses

Gene expression changes were based on PASS reads mapped to the 3’-most exon of a gene, represented by the reads per million total PASS reads (RPM) value. Venn diagrams were generated by VennDIS [62]. Gene Ontology (GO) analysis was carried out using the Fisher’s exact test. GO annotation of genes was obtained from the NCBI Gene database.

## Supporting Information

S1 Fig Western blot analysis of protein expression after knockdown (KD) for 48 hr. Percent of protein expression in KD cells relative to siCtrl cells is indicated.
(PDF)

S2 Fig Significance analysis of alternative polyadenylation (SAAP) and global analysis of alternative polyadenylation (GAAP). (A) Data sampling in SAAP. As described in Materials and Methods, pA1 and pA2 are two pAs or two pA sets, and Sa and Sb are two samples, a and b. The observed Relative Expression Difference (RED) is calculated by log2((a1/a2)/(b1/b2)). Data are sampled by bootstrapping based on the assumption that the relative abundance (RA) of each pA isoform is the same in two samples. There are *n* number of genes. Bootstrapping is carried out *m* times. Sa’ and Sb’ are expected data after sampling, which are used to calculate expected RED scores. Both observed and expected RED scores were standardized to Z values using mean and standard deviation (SD) obtained from expected RED scores. Zo is observed Z and Ze is expected Z. (B) Calculation of FDR and q-value in SAAP. Zc is Z cutoff value. FDR = (#{|Ze| > Zc} / m)/(#{|Zo| > Zc}), where #{|Ze| > Zc} is number of APA events with |Ze| > |Zc| and #{|Zo| > Zc}) is number of events with |Zo| > Zc. Q-value for an APA event x is the FDR using the absolute value of its Zo (Zo_x_) as Zc. (C) Schematic of GAAP. As described in Materials and Methods, A and B are two 3’READS data sets for two samples which are processed at the same time. For example, A is a KD sample and B is a control sample. A’ and B’ are randomly permutated data based on A and B with sample assignment shuffled. In permutation, poly(A) site-supporting (PASS) reads are randomly assigned to a set, with the total numbers of reads of the permutated sets A’ and B’ being the same as those of the original sets A and B, respectively. a and b are sampled by bootstrapping from A and B, respectively; a’ and b’ are data sets sampled by bootstrapping from A’ and B’, respectively. In this study, the total number of PASS reads in a, b, a’ and b’ are all 1.5M. Comparisons of a vs. b and a’ vs. b’ are carried out by SAAP. The result from a’ vs. b’ comparison provides expected values (exp), which are used to normalize observed values (obs) from the a vs. b comparison.
(PDF)

S3 Fig 3’UTR-APA. (A) Analysis of 3’UTR-APA using the top two most significantly regulated APA isoforms of a gene. Data are presented as in Fig 2D. The top two most significantly regulated APA isoforms were based on SAAP, comparing each pA with all other pAs in the same 3’UTR. (B) Percent of genes with different numbers of 3’UTR pAs (1, 2, 3 or > = 4) significantly regulated in different samples. For genes with at least two 3’UTR pAs, each pA was compared to all other pAs in the same 3’UTR using SAAP. Significant pAs are those with q-value < 0.05 (SAAP). (C) Regulation of 3’UTR length in different samples. For each sample, genes with significant 3’UTR shortening or lengthening (q-value < 0.05, SAAP) based on the top two most abundant isoforms were first selected and the aUTR sizes between the two pAs are plotted. The median value is indicated by a thick line and the interquartile range (between the 25^th^ and 75^th^ percentiles) is shown as a box.
(PDF)

S4 Fig CDS-APA. (A) Relative abundance of regulated CDS-APA isoforms in control C2C12 cells. The relative abundance of a pA isoform is the fraction of PASS reads for the pA of all PASS reads for the gene. The median value is indicated by a thick line and the interquartile range (between the 25^th^ and 75^th^ percentiles) is shown as a box. (B) Percent of regulated CDS-APA isoforms having pAs in introns or exons. Regulated CDS-APA isoforms are those with q-value < 0.05 (SAAP), based on comparison of one CDS-pA isoform with all other pA isoforms of the same gene.
(PDF)

S5 Fig Intronic APA regulated by siFip1 and siPcf11. (A) Regulation of intronic pA isoforms by siFip1 and siPcf11. Data are presented as in Fig 3C. (B) Features of introns containing pAs of isoforms downregulated by siFip1 or siPcf11. Data are presented as in Fig 3D.
(PDF)

S6 Fig Correlation of APA regulation between siCFI-25 and siCFI-68 samples. Only RED scores for 3’UTR-APA events are presented. Genes with or without significant APA changes are shown as red or gray dots, respectively. Q < 0.05 (SAAP) was used to select significantly regulated events.
(PDF)

S7 Fig Correlation of APA regulation between 48 hr and 32 hr KD samples. **(A)** Scatter plot of RED scores for 3’UTR-APA events in 48 hr KD samples (x-axis) vs. 32 hr KD (y-axis) samples. Genes with significant 3’UTR-APA (q-value < 0.05, SAAP) in both KD conditions are shown in red. Pearson correlation coefficient *r* based on all red dots is indicated in each graph. **(B)** Percent of genes with significant 3’UTR-APA regulation in both 48 hr and 32 hr KD samples are divided into 4 groups representing different consequences on 3’UTR size, as indicated next to the graph. Sh, 3’UTR shortened; Le, 3’UTR lengthened.
(PDF)

S8 Fig Correlation of APA regulation between total and nuclear RNAs of KD samples. Data are presented as in S7 Fig except that APA events identified with total RNA are compared with events identified with nuclear RNA.
(PDF)

S9 Fig Gene expression change vs. 3’UTR-APA regulation. Top, plots using data from nuclear RNA; bottom, plots using data from total RNA. For each plot, genes are divided into three groups based on 3’UTR-APA regulation, including 3’UTR shortened, lengthened, and no change, using SAAP (q-value < 0.05), and are shown in the graph with different colors. The dotted vertical lines indicate median values for different groups. Gene expression was calculated using all poly(A) site-supporting reads in the 3’-most exon. P-values (Kolmogorov–Smirnov test) indicated in each graph are based on comparison of gene expression between genes with 3’UTR shortened (blue) or lengthened (red) and genes with no 3’UTR changes.
(PDF)

S10 Fig Gene Ontology terms associated with gene groups shown in Fig 7B. Biological Process (top) and Cellular Component (bottom) terms were analyzed. Genes in each group were compared with genes in other groups by the Fisher’s exact test. Numbers are –log_10_(*P*), where *P* is based on the Fisher’s exact test.
(PDF)

S11 Fig KD level vs. extent of APA regulation. X-axis is log2(ratio) of protein expression as measured by Western Blot (S1 Fig); y-axis is the extent of 3’UTR-APA regulation derived from Fig 2D. Spearman and Pearson correlation coefficients are shown on the top with p-values indicating significance of correlation. siCFI-25 and siCFI-68 were not included in this plot because their APA values are substantially different than others.
(PDF)

S12 Fig Comparison of APA regulation between siPABPN1 and siRrp44 + siRrp6. (A) 3’UTR-APA. (B) CDS-APA. (C) Regulation of uaRNAs. (D) Regulation of spRNAs. Data are presented as in Figs 2–4.
(PDF)

S13 Fig Gene expression changes of the C/P and splicing factors examined in this study in C2C12 differentiation. Gene expression analysis was based on two 3’READS data sets. Error bars are standard error of mean. A total PASS read number >20 per gene was required to calculate the log2 ratio between proliferating and differentiated cells. Regulation of CFI-25 was not determined due to a small number of reads.
(PDF)

S1 Table siRNAs used in this study. (PDF)

S2 Table Antibodies used in this study. (PDF)

S3 Table RT-qPCR primers used in this study. (PDF)

S4 Table Number of reads mapped to pAs in each sample. (PDF)

S5 Table Bias of 4-mers to pAs regulated by five C/P factors. The data is also summarized in Fig 6B. Numbers are significance score (SS), which was calculated by –log_10_(*P*)*S, where *P* was based on the Fisher’s exact test and S = 1 for enrichment and -1 for depletion.
(PDF)

S6 Table Significant 6-mers biased to pAs regulated by five C/P factors. Only those with *P*≤0.001 (Fisher’s exact test) in any one of the comparisons are shown. Numbers are significance score (SS), which was calculated by –log_10_(*P*)*S, where *P* was based on the Fisher’s exact test and S = 1 for enrichment and -1 for depletion.
(PDF)

S7 Table Genes in different groups shown in Fig 7B. (PDF)
