# Supplementary figures and images for: Systematic Profiling of Poly(A)+ Transcripts Modulated by Core 3’ End Processing and Splicing Factors Reveals Regulatory Rules of Alternative Cleavage and Polyadenylation
Source: PLoS Genet. 2015 Apr 23;11(4):e1005166. doi: 10.1371/journal.pgen.1005166 (PMC4407891; doi:10.1371/journal.pgen.1005166)

Figure S1

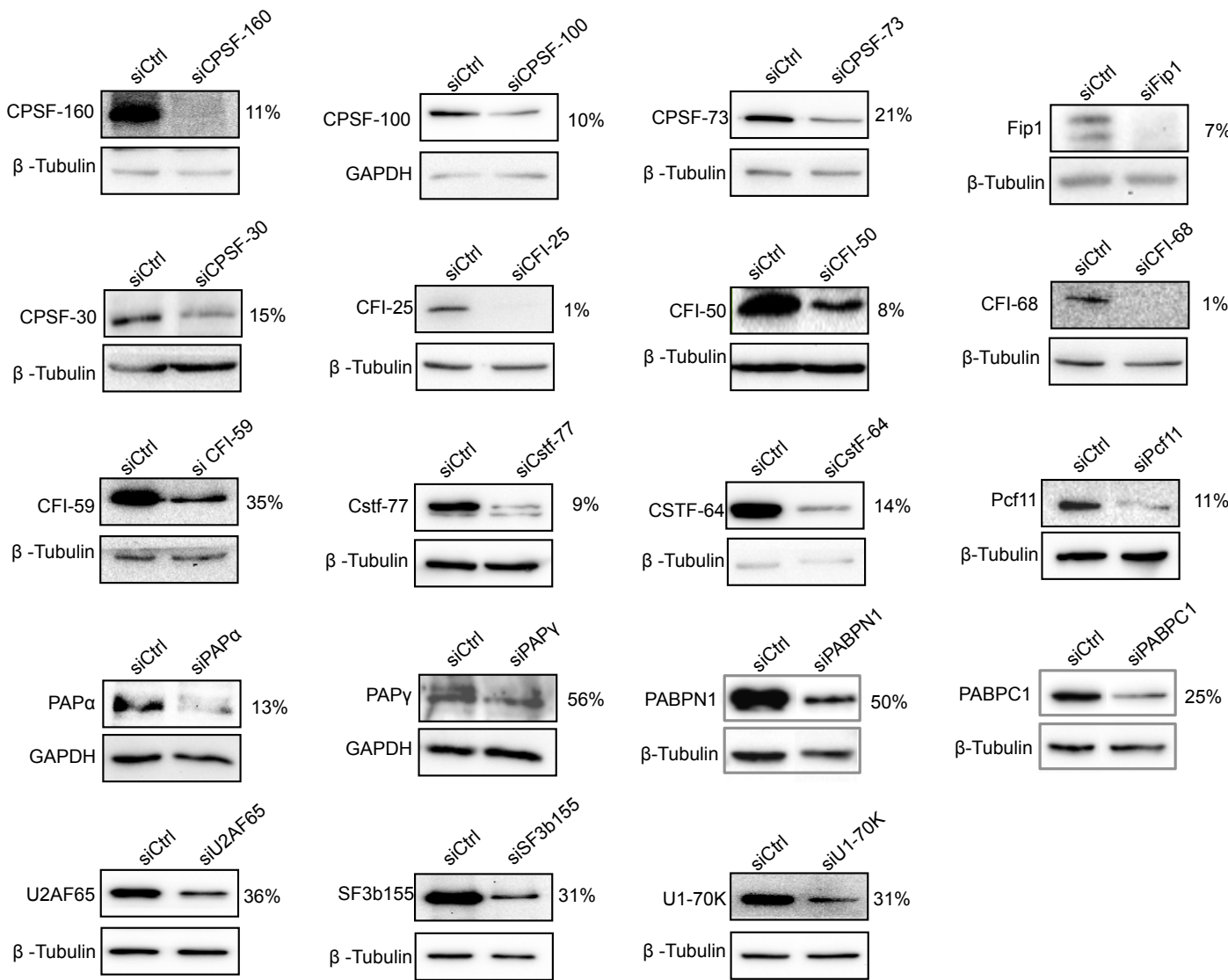

Supplement: S1 Fig — Percent of protein expression in KD cells relative to siCtrl cells is indicated. (PDF) [file pgen.1005166.s001.pdf]

Figure S3

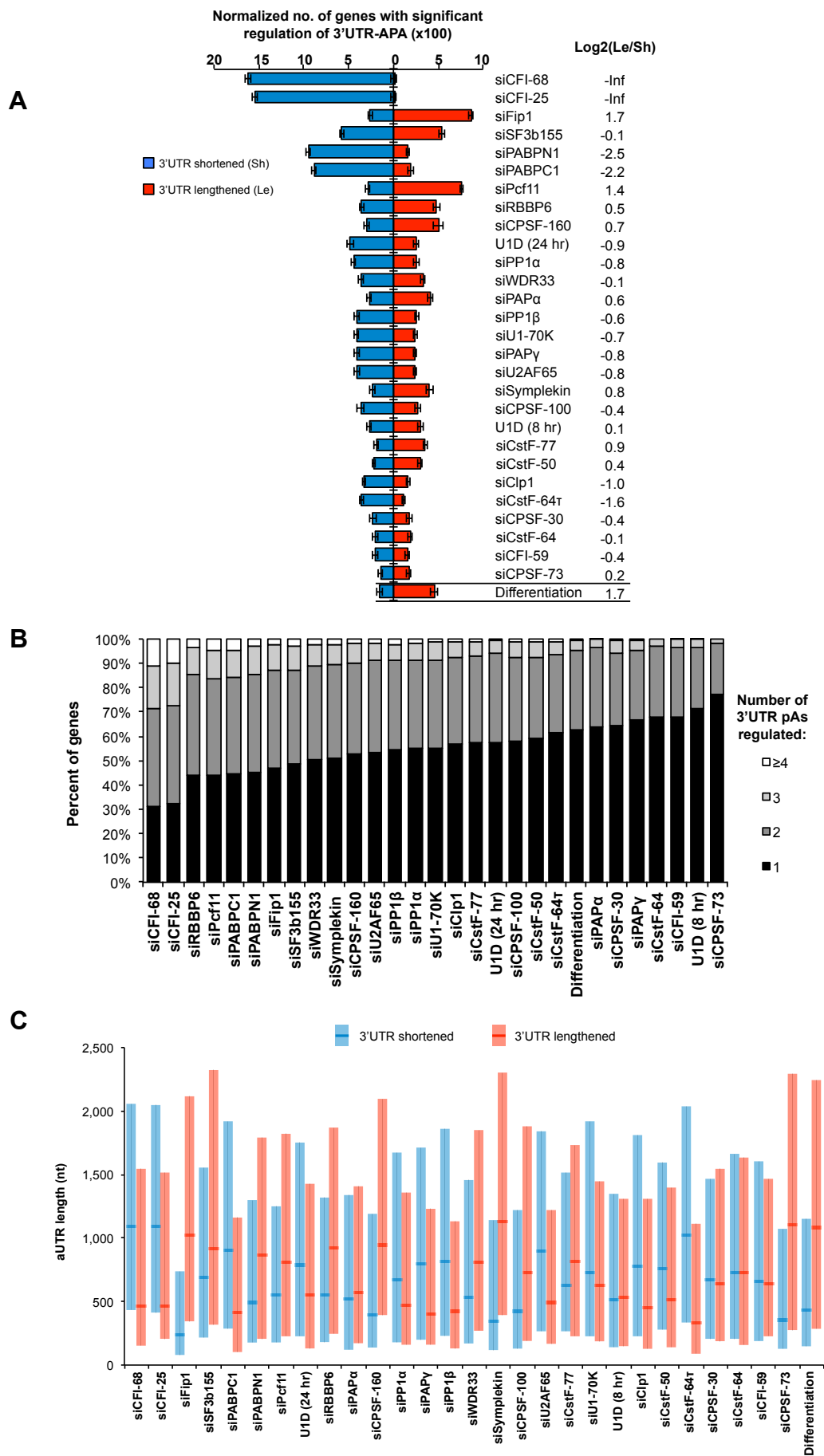

Supplement: S3 Fig — (A) Analysis of 3’UTR-APA using the top two most significantly regulated APA isoforms of a gene. Data are presented as in Fig 2D. The top two most significantly regulated APA isoforms were based on SAAP, comparing each pA with all other pAs in the same 3’UTR. (B) Percent of genes with different numbers of 3’UTR pAs (1, 2, 3 or > = 4) significantly regulated in different samples. For genes with at least two 3’UTR pAs, each pA was compared to all other pAs in the same 3’UTR using SAAP. Significant pAs are those with q-value < 0.05 (SAAP). (C) Regulation of 3’UTR length in different samples. For each sample, genes with significant 3’UTR shortening or lengthening (q-value < 0.05, SAAP) based on the top two most abundant isoforms were first selected and the aUTR sizes between the two pAs are plotted. The median value is indicated by a thick line and the interquartile range (between the 25th and 75th percentiles) is shown as a box. (PDF) [file pgen.1005166.s003.pdf]

Figure S4

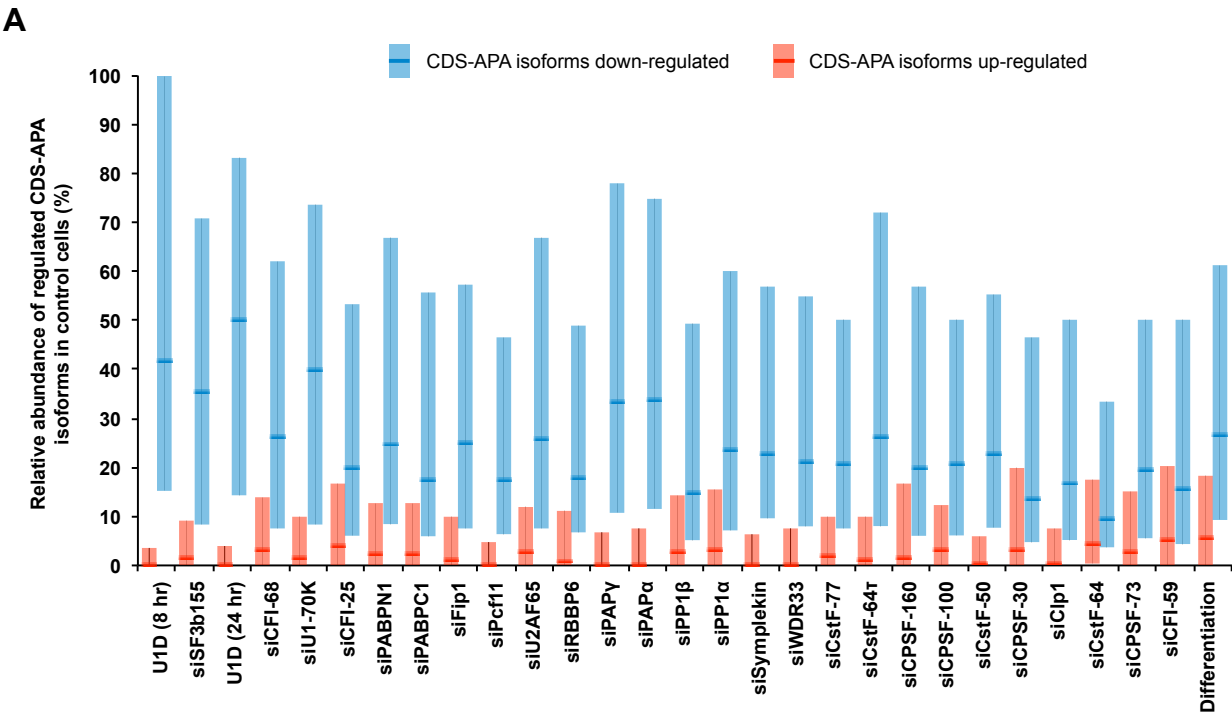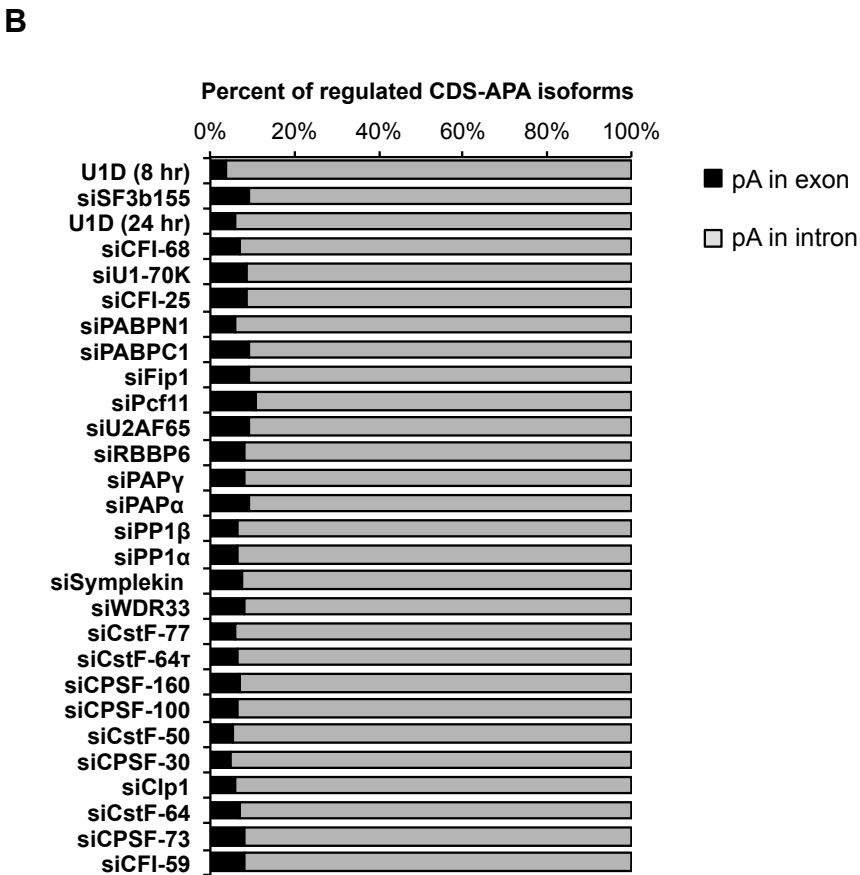

Supplement: S4 Fig — (A) Relative abundance of regulated CDS-APA isoforms in control C2C12 cells. The relative abundance of a pA isoform is the fraction of PASS reads for the pA of all PASS reads for the gene. The median value is indicated by a thick line and the interquartile range (between the 25th and 75th percentiles) is shown as a box. (B) Percent of regulated CDS-APA isoforms having pAs in introns or exons. Regulated CDS-APA isoforms are those with q-value < 0.05 (SAAP), based on comparison of one CDS-pA isoform with all other pA isoforms of the same gene. (PDF) [file pgen.1005166.s004.pdf]

Figure S5

A

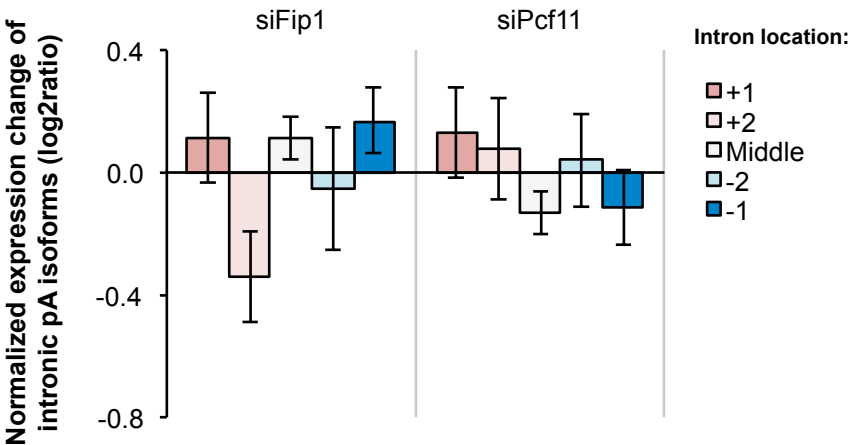

B

| Sample  | Intron size |    |   |    |    | 5'SS strength |    |   |    |    | 3'SS strength |    |   |    |    |
|---------|-------------|----|---|----|----|---------------|----|---|----|----|---------------|----|---|----|----|
|         | +1          | +2 | M | -2 | -1 | +1            | +2 | M | -2 | -1 | +1            | +2 | M | -2 | -1 |
| siFip1  | 0           | -1 | 0 | -1 | 0  | 0             | 0  | 0 | 1  | 0  | 0             | 0  | 1 | 0  | 0  |
| siPcf11 | 3           | 0  | 0 | 1  | 0  | 1             | 0  | 1 | 0  | 0  | 1             | 0  | 1 | 0  | 0  |

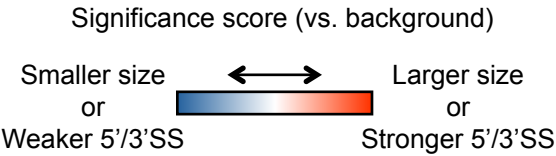

Supplement: S5 Fig — (A) Regulation of intronic pA isoforms by siFip1 and siPcf11. Data are presented as in Fig 3C. (B) Features of introns containing pAs of isoforms downregulated by siFip1 or siPcf11. Data are presented as in Fig 3D. (PDF) [file pgen.1005166.s005.pdf]

Figure S6

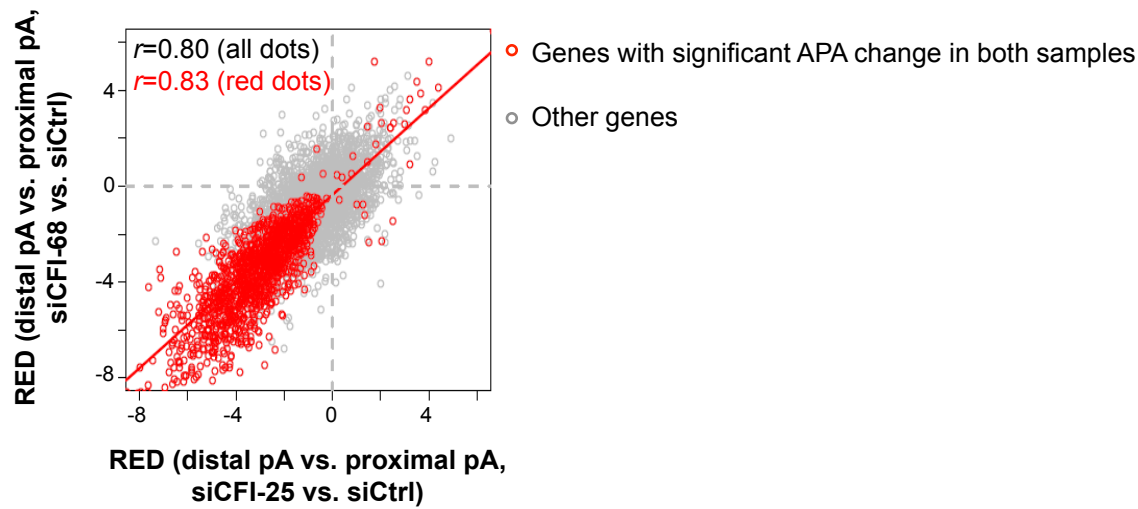

Supplement: S6 Fig — Only RED scores for 3’UTR-APA events are presented. Genes with or without significant APA changes are shown as red or gray dots, respectively. Q < 0.05 (SAAP) was used to select significantly regulated events. (PDF) [file pgen.1005166.s006.pdf]

Figure S7

A

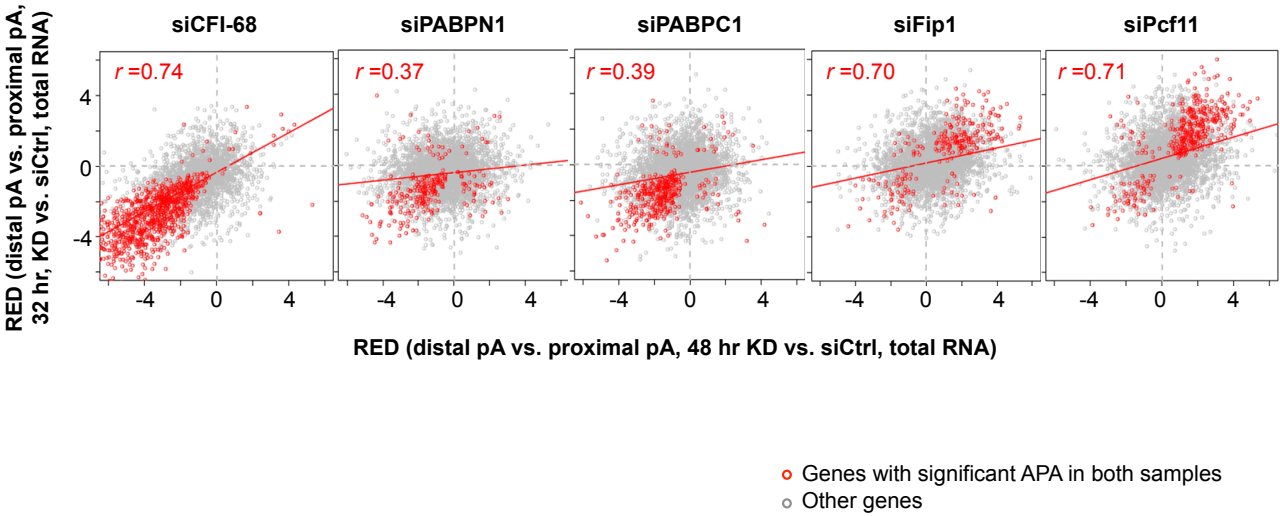

B

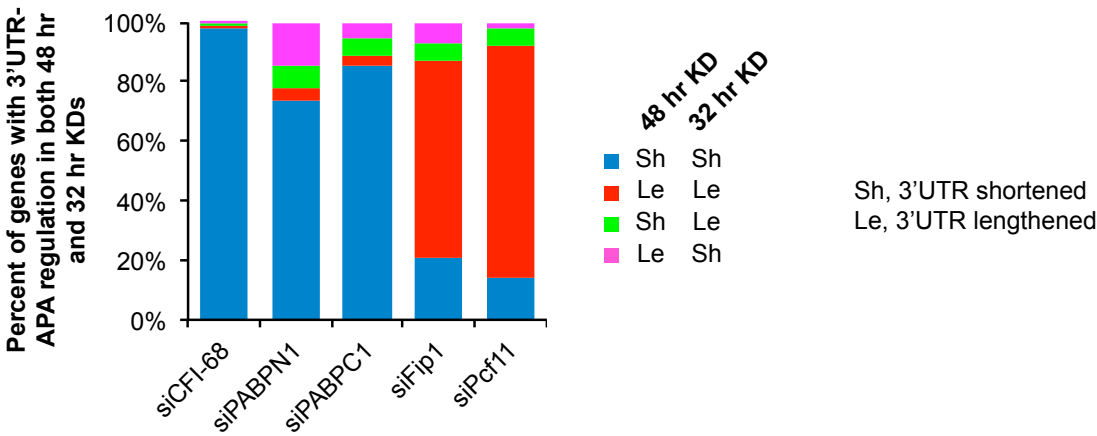

Supplement: S7 Fig — (A) Scatter plot of RED scores for 3’UTR-APA events in 48 hr KD samples (x-axis) vs. 32 hr KD (y-axis) samples. Genes with significant 3’UTR-APA (q-value < 0.05, SAAP) in both KD conditions are shown in red. Pearson correlation coefficient r based on all red dots is indicated in each graph. (B) Percent of genes with significant 3’UTR-APA regulation in both 48 hr and 32 hr KD samples are divided into 4 groups representing different consequences on 3’UTR size, as indicated next to the graph. Sh, 3’UTR shortened; Le, 3’UTR lengthened. (PDF) [file pgen.1005166.s007.pdf]

Figure S8

A

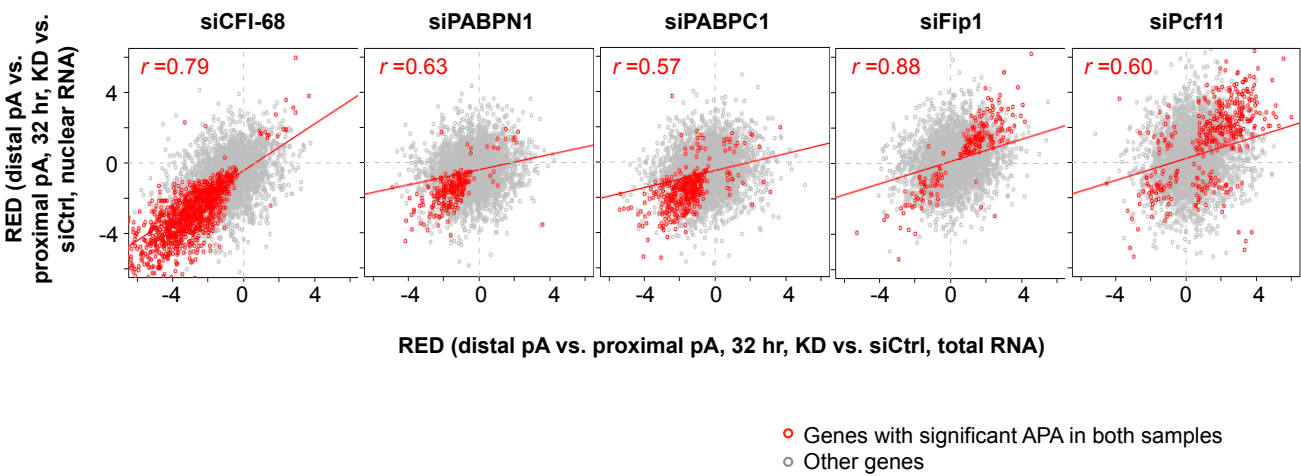

B

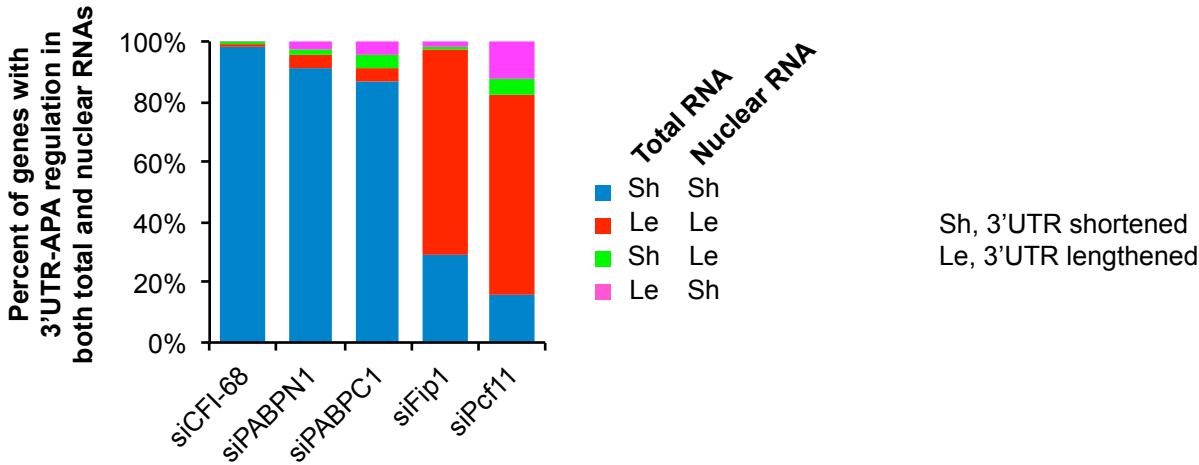

Supplement: S8 Fig — Data are presented as in S7 Fig except that APA events identified with total RNA are compared with events identified with nuclear RNA. (PDF) [file pgen.1005166.s008.pdf]

Figure S9

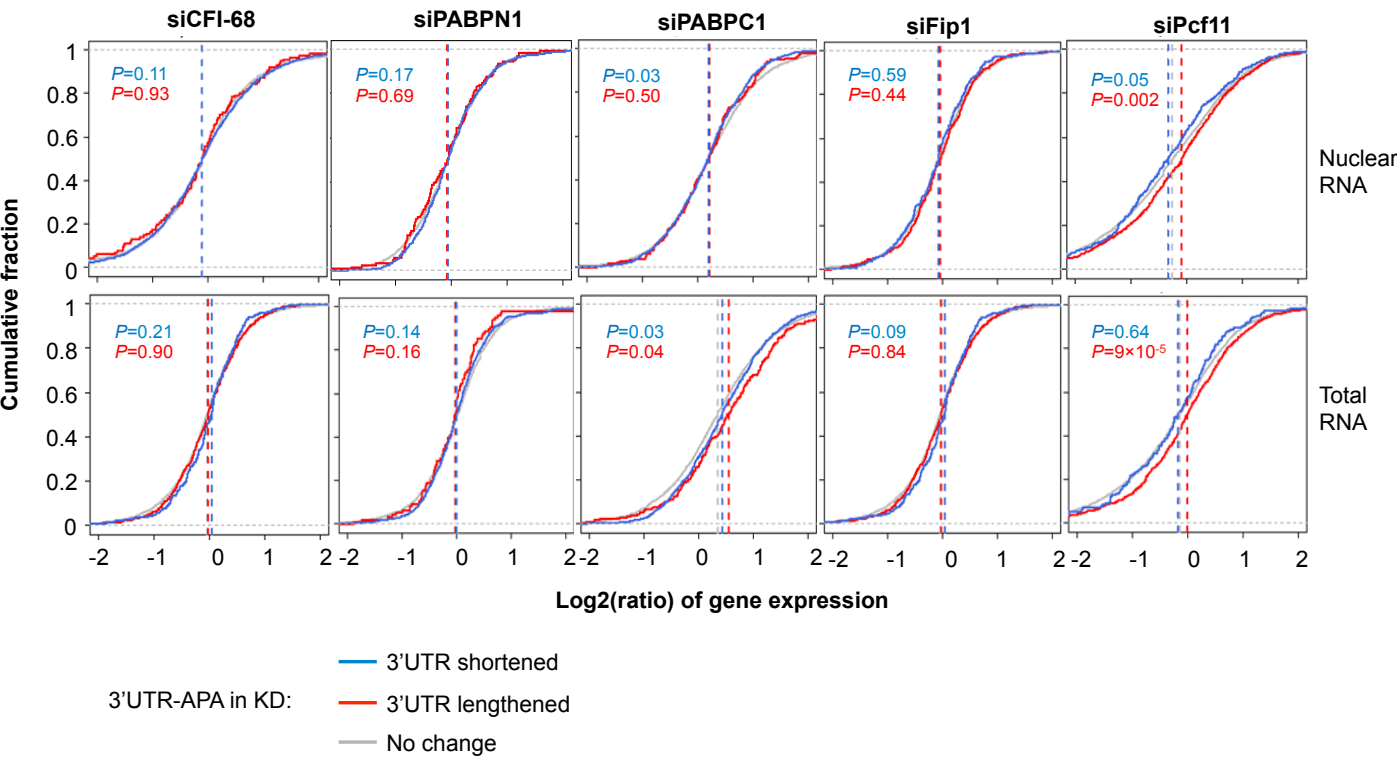

Supplement: S9 Fig — Top, plots using data from nuclear RNA; bottom, plots using data from total RNA. For each plot, genes are divided into three groups based on 3’UTR-APA regulation, including 3’UTR shortened, lengthened, and no change, using SAAP (q-value < 0.05), and are shown in the graph with different colors. The dotted vertical lines indicate median values for different groups. Gene expression was calculated using all poly(A) site-supporting reads in the 3’-most exon. P-values (Kolmogorov–Smirnov test) indicated in each graph are based on comparison of gene expression between genes with 3’UTR shortened (blue) or lengthened (red) and genes with no 3’UTR changes. (PDF) [file pgen.1005166.s009.pdf]

Figure S11

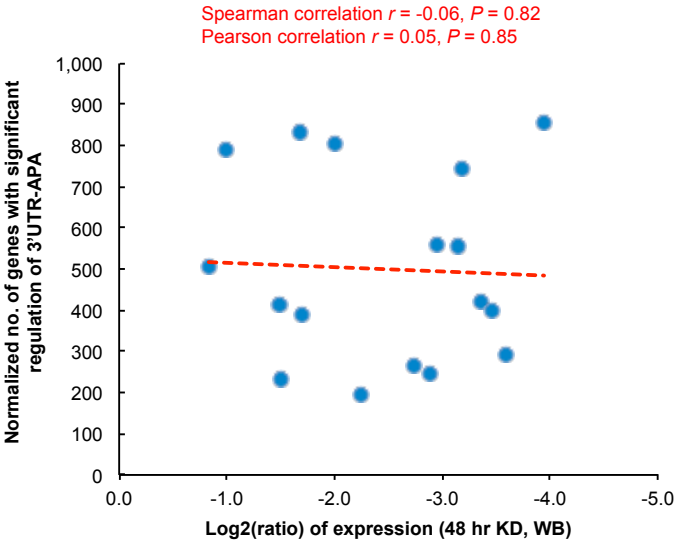

Supplement: S11 Fig — X-axis is log2(ratio) of protein expression as measured by Western Blot (S1 Fig); y-axis is the extent of 3’UTR-APA regulation derived from Fig 2D. Spearman and Pearson correlation coefficients are shown on the top with p-values indicating significance of correlation. siCFI-25 and siCFI-68 were not included in this plot because their APA values are substantially different than others. (PDF) [file pgen.1005166.s011.pdf]

Figure S12

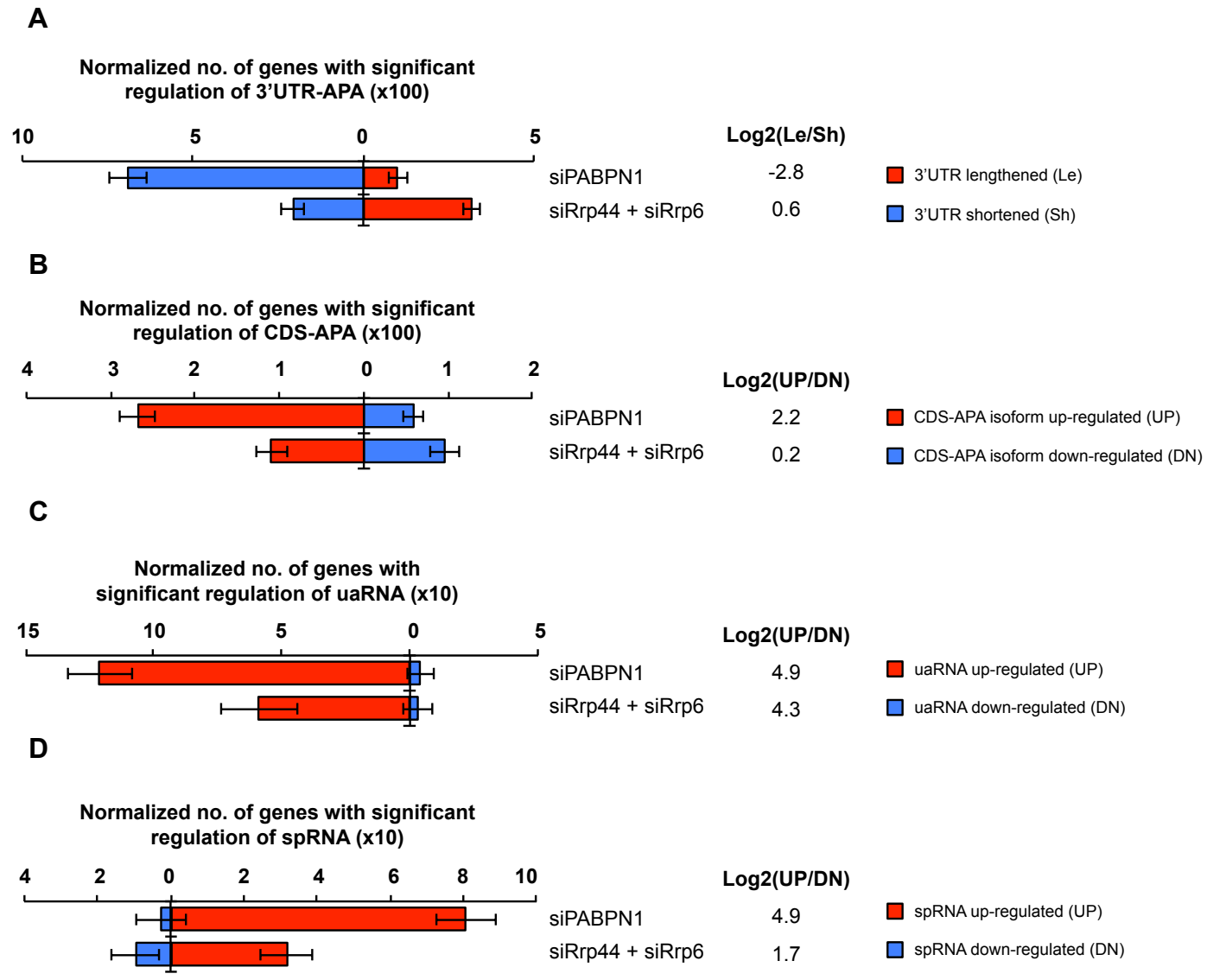

Supplement: S12 Fig — (A) 3’UTR-APA. (B) CDS-APA. (C) Regulation of uaRNAs. (D) Regulation of spRNAs. Data are presented as in Figs 2–4. (PDF) [file pgen.1005166.s012.pdf]

Figure S13

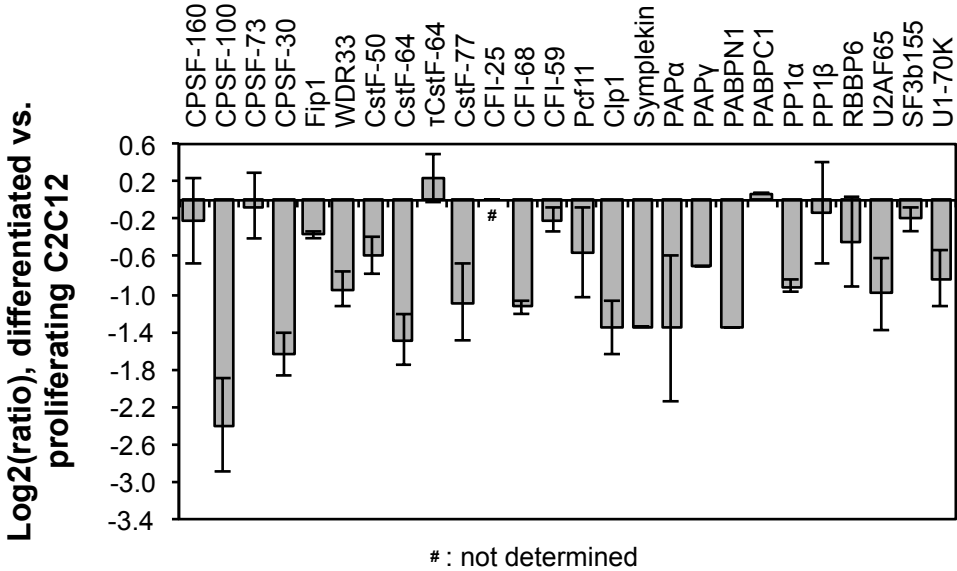

Supplement: S13 Fig — Gene expression analysis was based on two 3’READS data sets. Error bars are standard error of mean. A total PASS read number >20 per gene was required to calculate the log2 ratio between proliferating and differentiated cells. Regulation of CFI-25 was not determined due to a small number of reads. (PDF) [file pgen.1005166.s013.pdf]
