# Supplementary material for: Systematic Profiling of Poly(A)+ Transcripts Modulated by Core 3’ End Processing and Splicing Factors Reveals Regulatory Rules of Alternative Cleavage and Polyadenylation
Source: PLoS Genet. 2015 Apr 23;11(4):e1005166. doi: 10.1371/journal.pgen.1005166 (PMC4407891; doi:10.1371/journal.pgen.1005166)
Supplement: S2 Fig — (A) Data sampling in SAAP. As described in Materials and Methods, pA1 and pA2 are two pAs or two pA sets, and Sa and Sb are two samples, a and b. The observed Relative Expression Difference (RED) is calculated by log2((a1/a2)/(b1/b2)). Data are sampled by bootstrapping based on the assumption that the relative abundance (RA) of each pA isoform is the same in two samples. There are n number of genes. Bootstrapping is carried out m times. Sa’ and Sb’ are expected data after sampling, which are used to calculate expected RED scores. Both observed and expected RED scores were standardized to Z values using mean and standard deviation (SD) obtained from expected RED scores. Zo is observed Z and Ze is expected Z. (B) Calculation of FDR and q-value in SAAP. Zc is Z cutoff value. FDR = (#{|Ze| > Zc} / m)/(#{|Zo| > Zc}), where #{|Ze| > Zc} is number of APA events with |Ze| > |Zc| and #{|Zo| > Zc}) is number of events with |Zo| > Zc. Q-value for an APA event x is the FDR using the absolute value of its Zo (Zox) as Zc. (C) Schematic of GAAP. As described in Materials and Methods, A and B are two 3’READS data sets for two samples which are processed at the same time. For example, A is a KD sample and B is a control sample. A’ and B’ are randomly permutated data based on A and B with sample assignment shuffled. In permutation, poly(A) site-supporting (PASS) reads are randomly assigned to a set, with the total numbers of reads of the permutated sets A’ and B’ being the same as those of the original sets A and B, respectively. a and b are sampled by bootstrapping from A and B, respectively; a’ and b’ are data sets sampled by bootstrapping from A’ and B’, respectively. In this study, the total number of PASS reads in a, b, a’ and b’ are all 1.5M. Comparisons of a vs. b and a’ vs. b’ are carried out by SAAP. The result from a’ vs. b’ comparison provides expected values (exp), which are used to normalize observed values (obs) from the a vs. b comparison. (PDF) [file pgen.1005166.s002.pdf]

Figure S2

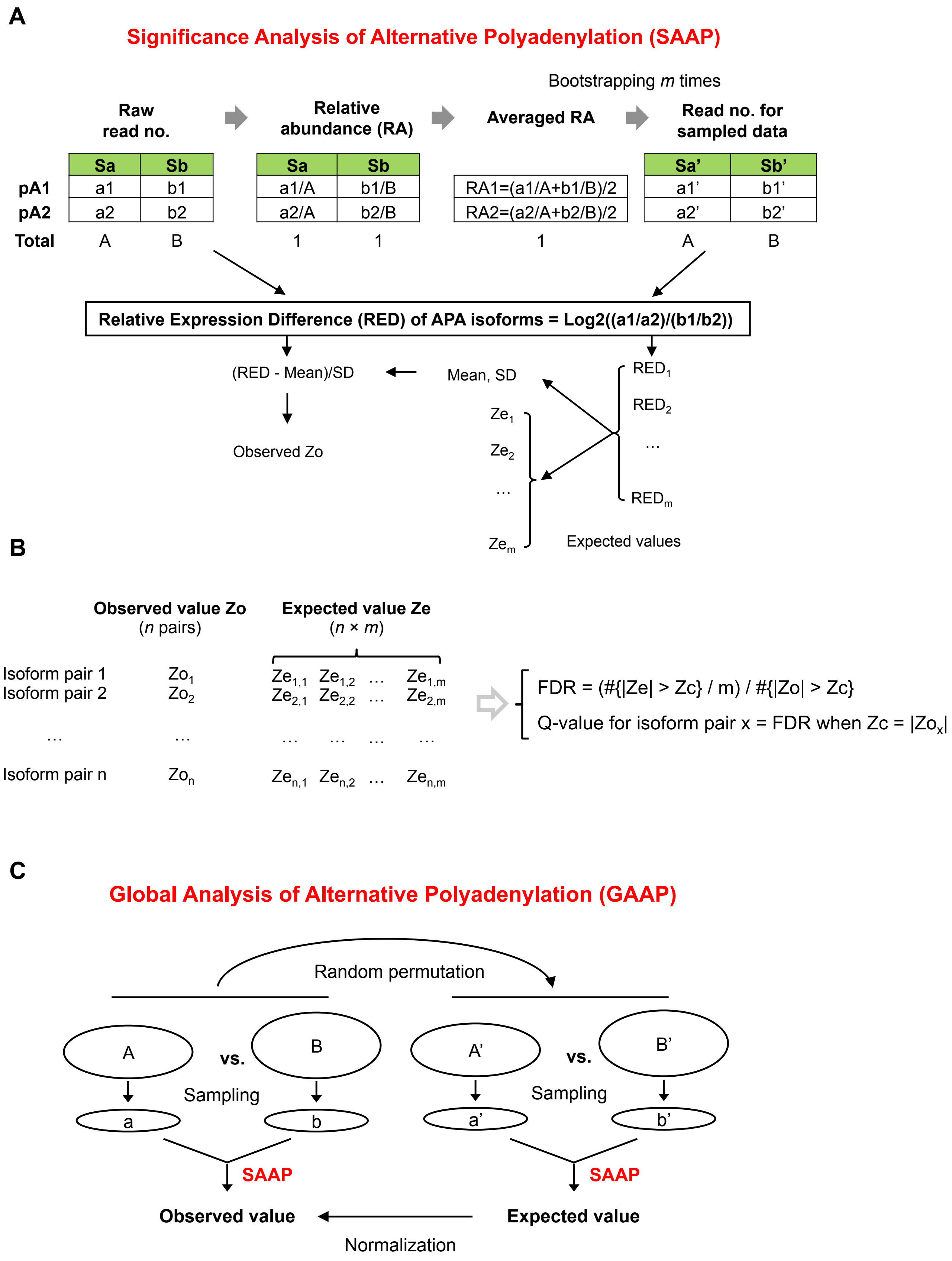
A
Significance Analysis of Alternative Polyadenylation (SAAP)
Bootstrapping m times
Raw read no.
Relative abundance (RA)
Averaged RA
Read no. for sampled data
Sa Sb
pA1 a1 b1
pA2 a2 b2
Total A B
Sa Sb
a1/A b1/B
a2/A b2/B
1 1
RA1=(a1/A+b1/B)/2
RA2=(a2/A+b2/B)/2
1
Sa' Sb'
a1' b1'
a2' b2'
A B
Relative Expression Difference (RED) of APA isoforms = Log2((a1/a2)/(b1/b2))
(RED - Mean)/SD
Mean, SD
Observed Zo
Ze_1
Ze_2
…
Ze_m
RED_1
RED_2
…
RED_m
Expected values
B
Observed value Zo
(n pairs)
Expected value Ze
(n × m)
Isoform pair 1 Zo_1 $Ze_{1,1}$ $Ze_{1,2}$ … $Ze_{1,m}$
Isoform pair 2 Zo_2 $Ze_{2,1}$ $Ze_{2,2}$ … $Ze_{2,m}$
… … … … … …
Isoform pair n Zo_n $Ze_{n,1}$ $Ze_{n,2}$ … $Ze_{n,m}$
FDR = (#{|Ze| > Zc} / m) / #{|Zo| > Zc}
Q-value for isoform pair x = FDR when Zc = $|Zo_x|$
C
Global Analysis of Alternative Polyadenylation (GAAP)
Random permutation
A vs. B
A' vs. B'
Sampling
a b
a' b'
SAAP
SAAP
Observed value
Expected value
Normalization
