# Supplementary material for: Systematic Profiling of Poly(A)+ Transcripts Modulated by Core 3’ End Processing and Splicing Factors Reveals Regulatory Rules of Alternative Cleavage and Polyadenylation
Source: PLoS Genet. 2015 Apr 23;11(4):e1005166. doi: 10.1371/journal.pgen.1005166 (PMC4407891; doi:10.1371/journal.pgen.1005166)
Supplement: S10 Fig — Biological Process (top) and Cellular Component (bottom) terms were analyzed. Genes in each group were compared with genes in other groups by the Fisher’s exact test. Numbers are –log10(P), where P is based on the Fisher’s exact test. (PDF) [file pgen.1005166.s010.pdf]

# Figure S10

**Gene groups shown in Fig. 7B**

**Biological Process**

| 1 | 2 | 3 | 4 | 5 | 7 | 8 | 9 | 10 | |
|---|---|---|---|---|---|---|---|---|---|
| 5.5 | 2.1 | 0.2 | 0.2 | 0.2 | 0.2 | 0.2 | 0.0 | 0.0 | regulation of phosphorylation |
| 4.4 | 0.2 | 0.8 | 0.0 | 0.3 | 0.5 | 0.8 | 0.1 | 0.0 | cell death |
| 4.1 | 1.4 | 0.2 | 0.3 | 0.2 | 0.5 | 0.3 | 0.3 | 0.0 | intracellular signal transduction |
| 0.3 | 0.0 | 0.1 | 0.3 | 0.2 | 0.0 | 0.0 | 3.9 | 0.1 | adaptive immune response based on somatic recombination of immune receptors built from… |
| 3.5 | 0.6 | 0.2 | 0.2 | 0.0 | 0.2 | 0.0 | 0.3 | 0.0 | actin polymerization or depolymerization |
| 0.6 | 0.4 | 3.5 | 0.2 | 0.1 | 0.2 | 0.2 | 0.3 | 0.0 | single-organism membrane organization |
| 0.2 | 0.2 | 3.5 | 0.1 | 0.5 | 0.0 | 0.9 | 0.3 | 0.0 | spermatogenesis |
| 3.3 | 0.7 | 0.2 | 0.0 | 0.0 | 0.0 | 0.0 | 0.1 | 0.0 | retina development in camera-type eye |
| 0.0 | 3.3 | 0.0 | 0.0 | 0.0 | 0.0 | 0.8 | 0.0 | 0.1 | endosome to lysosome transport |
| 3.2 | 0.0 | 0.4 | 0.2 | 0.2 | 0.2 | 0.0 | 0.3 | 0.3 | steroid metabolic process |
| 0.3 | 0.1 | 0.0 | 1.1 | 0.1 | 3.2 | 0.1 | 0.3 | 0.2 | cellular carbohydrate metabolic process |
| 0.2 | 3.1 | 0.6 | 0.0 | 0.0 | 0.2 | 0.3 | 0.1 | 0.0 | transforming growth factor beta receptor signaling pathway |
| 0.2 | 3.1 | 0.5 | 0.0 | 0.1 | 0.2 | 0.3 | 0.0 | 0.0 | cellular response to endogenous stimulus |
| 1.2 | 0.0 | 0.4 | 0.2 | 0.8 | 0.1 | 3.1 | 0.6 | 0.1 | oxidation-reduction process |
| 0.3 | 0.0 | 3.0 | 0.0 | 0.2 | 0.0 | 0.0 | 0.0 | 0.3 | actomyosin structure organization |
| 3.0 | 0.6 | 0.3 | 0.0 | 0.2 | 0.5 | 0.0 | 0.0 | 0.0 | cell fate specification |
| 0.3 | 0.2 | 3.0 | 0.2 | 0.5 | 0.6 | 0.3 | 0.1 | 0.0 | intracellular protein transport |
| 1.8 | 3.0 | 0.1 | 0.0 | 0.5 | 0.1 | 0.0 | 0.1 | 0.0 | regulation of cellular component movement |

**Cellular component**

| 1 | 2 | 3 | 4 | 5 | 7 | 8 | 9 | 10 | |
|---|---|---|---|---|---|---|---|---|---|
| 0.9 | 4.9 | 0.4 | 0.3 | 0.2 | 0.4 | 0.1 | 0.2 | 0.0 | cell leading edge |
| 0.5 | 1.9 | 4.1 | 0.0 | 0.1 | 0.5 | 0.5 | 0.0 | 0.0 | cell body |
| 2.8 | 0.0 | 0.6 | 0.1 | 0.2 | 0.0 | 0.6 | 3.9 | 0.0 | mitochondrial envelope |
| 0.0 | 3.7 | 0.0 | 0.0 | 0.3 | 0.4 | 0.0 | 0.0 | 0.0 | coated vesicle membrane |
| 0.2 | 0.2 | 0.1 | 0.0 | 0.0 | 0.0 | 3.6 | 0.2 | 0.2 | oxidoreductase complex |
| 0.6 | 1.8 | 3.5 | 0.1 | 0.1 | 0.2 | 0.4 | 0.0 | 0.0 | cytoplasmic vesicle |
| 0.7 | 3.5 | 0.3 | 0.1 | 0.1 | 0.2 | 0.2 | 0.2 | 0.0 | cell junction |
| 1.3 | 3.4 | 1.2 | 0.1 | 0.1 | 0.0 | 1.2 | 0.2 | 0.0 | endomembrane system |
| 0.1 | 0.2 | 0.6 | 0.3 | 3.1 | 0.0 | 0.0 | 0.0 | 0.1 | catalytic step 2 spliceosome |
