# Supplementary material for: Systematic Profiling of Poly(A)+ Transcripts Modulated by Core 3’ End Processing and Splicing Factors Reveals Regulatory Rules of Alternative Cleavage and Polyadenylation
Source: PLoS Genet. 2015 Apr 23;11(4):e1005166. doi: 10.1371/journal.pgen.1005166 (PMC4407891; doi:10.1371/journal.pgen.1005166)
Supplement: S1 Table — (PDF) [file pgen.1005166.s014.pdf]

Table S1. siRNAs used in this study

Factor	siRNA sequence[1]
CPSF-160	A: 5'-CCGAGCGUUUCACUUUGACAATT, 5'-UUGUCAAAGUGAAACGCUCGGTT<br>B: 5'-CCGCAAUCUUAUGGUGUAUATT, 5'-AUAUACACCAUAAGAUUGCGGTT
CPSF-100	5'-CUCGACACAAUACAGAAGAUUTT, 5'-AAUCUUCUGUAUUGUGUCGAGTT
CPSF-73	5'-CCAGCAAACCAGUGAAUUUAUTT, 5'-AUAAAUUCACUGGUUUGCUGGTT
CPSF-30	5'-ACGCCAACAGAUGCACCAAAGTT, 5'-CUUUGGUGCAUCUGUUGGCGUTT
Fip1	A: 5'-GUGCUGAUCUUUCUGAUUATT, 5'-UAAUCAGAAAGAUCAGCACTT<br>B: 5'-CGUCGCCAUGAAAGUGAAGAATT, 5'-UUCUUCACUUUCAUGGCGACGTT
WDR33	5'-CUCCAAAUGAGGUGCUGAAUTT, 5'-AUUCAGCACCUCAUUUGGAGTT
CstF-50	5'-GCUCUACGACGGUUACAUUTT, 5'-AAUGUAACCGUCGUAGAGCAG
CstF-64	A: 5'-GCUGGCAACAAAUCUGGAAAUTT, 5'-AUUUCCAGAUUUGUUGCCAGCTT<br>B: 5'-GCACAGGUAGUGAUGAGAAUUTT, 5'-AAUUCUCAUCACUACCUGUGCTT
CstF-64τ	5'-GCACAAGUGGUGAUGAGAATT, 5'-UUCUCAUCACCACUUGUGCCT
CstF-77	5'-CGAGGAUUCAGACGAAGAUTT, 5'-AUCUUCGUCUGAAUCCUCGTT
CFI-25	A: 5'-CGUCUCUAUGAGCACAGCUUATT, 5'-UAAGCUGUGCUCAUAGAGACGTT<br>B: 5'-GCACCAUUGUUUGAGCUGUAUTT, 5'-AUACAGCUCAAACAAUGGUGCTT
CFI-68	A: 5'-CCUGUUGUAACUCCAUGCAAUTT, 5'-AUUGCAUGGAGUUACAACAGGTT<br>B: 5'-CCGUUCAUUCUUUGGGAGUAATT, 5'-UUACUCCCAAAGAAUGAACGGTT
CFI-59	5'-GUCCUCAUCUCCUCUCUUATT, 5'-UAAGAGAGGAGAUGAGGACTT
Pcf11[2]	A: 5'-CGACAGCUAUUUCAGUAUCAATT, 5'-UUGAUACUGAAAUAGCUGUCGTT<br>B: 5'-GGGCAAAGAUGAAGAUGUATT, 5'-UACAUCUUCAUCUUUGCCCTT
Clp1	A: 5'-GAGAUGAACGUAUCCGUGATT, 5'-UCACGGAUACGUUCAUCUCTA<br>B: 5'-CGUCCUACUUACGUGGAGUUATT, 5'-UAACUCCACGUAAGUAGGACGTT
Symplekin	5'-UCAGGCACUCUGGACAAAUAUTT, 5'-AUAUUUGUCCAGAGUGCCUGATT
PAPα	5'-UUGCAGGGUAACCGAUGAAAUTT, 5'-AUUUCAUCGGUUACCCUGCAATT
PAPγ	5'-AUGUGGUUCCUUGGGAUAAUUTT, 5'-AAUUAUCCCAAGGAACCACAUTT
PABPN1[2]	A: 5'-UAGAGCGACAUCAUGGUAUUCTT, 5'-GAAUACCAUGAUGUCGCUCUATT<br>B: 5'-ACAGUUUGGUGUCCUUCCAGAGAGC, 5'-GCTCTCTGGUUGGUCUCCUUUCTGT
PABPC1[2]	A: 5'-CGUGCUUUGGACACCAUGAAUTT, 5'-AUUCAUGGUGUCCAAAGCACGTT<br>B: 5'-UAAAGCUACAUACAGUGGCUUCGUG, 5'-CUCGUUGCCUCTGTUTGTUGCTTTU
PP1α	5'-CCAGAUCGUUUGUACAGAAAUTT, 5'-AUUUCUGUACAAACGAUCUGGTT
PP1β	5'-GAGGAAACCAUGAGUGUGCUATT, 5'-UAGCACACUCAUGGUUUCCUCTT
RBBP6	A: 5'-GAUUGUCAGGAGGAUUCCUAUTT, 5'-AUAGGAAUCCUCCUGACAAUCTT<br>B: 5'-CGAUCACUUUCAAGUCAGUAUTT, 5'-AUACUGACUUGAAAGUGAUCGTT
U2AF65	A: 5'-GCAACUGGAAUGGCAGCAAUUTT, 5'-AAUUGCUGCCAUUCCAGUUGCTT<br>B: 5'-GUGAGUACGUGGACAUCAATT, 5'-UUGAUGUCCACGUACUCACTT
SF3b155	A: 5'-GCCUGCCUUAAUGAAUGAAUATT, 5'-UAUUCAUUCAUUAAGGCAGGCTT<br>B: 5'-GUCACUUGGUGUUUACGGATT, 5'-UCCGUAAACACCAAGUGACAT
U1-70K	A: 5'-GCACCAUACAUCCGAGAGUUUTT, 5'-AAACUCUCGGAUGUAUGGUGCTT<br>B: 5'-CCCUCACAAUGAUCCCAAUTT, 5'-AUUGGGAUCAUUGUGAGGGTC
RRP44	A: 5'-GCGUUAGAAGGACGGAGAAUUTT, 5'-AAUUCUCCGUCCUUCUAACGCTT<br>B: 5'-GCGGCUAUGAAUGAUGAUATT, 5'-UAUCAUCAUUCAUAGCCGCTT
RRP6	A: 5'-GUUCGGUGACGAGUAUGAUUUTT, 5'-AAAUCAUACUCGUCACCGAACTT<br>B: 5'-GGUCCAGAAAGAACCUAAATT, 5'-UUUAGGUUCUUUCUGGACCTT
siCtrl[3]	5'-UUCUCCGAACGUGUCACGUTT, 5'-ACGUGACACGUUCGGAGAATT

[1]Some knockdowns include two siRNAs. They are indicated by A and B.
[2]Only siRNA A was used for 48 hr knockdown samples. Both A and B siRNAs were used for 32 hr knockdown samples.
[3]siRNA negative control is a random sequence with no detectable homology with any mammalian genes (obtained from GenePharma).
