# Supplementary material for: Systematic Profiling of Poly(A)+ Transcripts Modulated by Core 3’ End Processing and Splicing Factors Reveals Regulatory Rules of Alternative Cleavage and Polyadenylation
Source: PLoS Genet. 2015 Apr 23;11(4):e1005166. doi: 10.1371/journal.pgen.1005166 (PMC4407891; doi:10.1371/journal.pgen.1005166)
Supplement: S2 Table — (PDF) [file pgen.1005166.s015.pdf]

Table S2. Antibodies used in this study.

Factor	Company / source	Catalog No.
CPSF-160	Abcam	ab81552
CPSF-100	Santa Cruz Biotechnology	sc-165983
CPSF-73	Bethyl Laboratories, Inc.	A301-091A
CPSF-30	Santa Cruz Biotechnology	sc-133480
Fip1	Abcam	ab80271
CstF-50	Novus Biologicals, LLC	nb100-60443
CstF-64	Gift from Dr. Clint McDonald	-
CstF-77	Bethyl Laboratories, Inc.	A301-094A
CFI-25	Santa Cruz Biotechnology	sc-81109
CFI-68	Santa Cruz Biotechnology	sc-100692
CFI-59	Bethyl Laboratories, Inc.	A301-360A
Pcf11	Santa Cruz Biotechnology	sc-161998
PAPα	Bethyl Laboratories, Inc.	A301-010A
PAPγ	Bethyl Laboratories, Inc.	A302-427A
PABPN1	Abcam	Ab75855
PABPC1	Abcam	Ab21060
U2AF65	Santa Cruz Biotechnology	sc-53942
SF3b155	Bethyl Laboratories, Inc.	A300-996A
U1-70K	Santa Cruz Biotechnology	sc-9571
