# Supplementary material for: Systematic Profiling of Poly(A)+ Transcripts Modulated by Core 3’ End Processing and Splicing Factors Reveals Regulatory Rules of Alternative Cleavage and Polyadenylation
Source: PLoS Genet. 2015 Apr 23;11(4):e1005166. doi: 10.1371/journal.pgen.1005166 (PMC4407891; doi:10.1371/journal.pgen.1005166)
Supplement: S3 Table — (PDF) [file pgen.1005166.s016.pdf]

Table S3. RT-qPCR primers used in this study

Factor	Primer sequence
CPSF-160	5'-CCAAGAAGATAGGTACCACGCCT, 5'-CACCAGAGCAGCATCCTAGAGTTAG
CPSF-100	5'-CAACAATCAAGTAGCAGTTCGCAG, 5'-GGCATACTGTTCATATAAAGGTCTCG
CPSF-73	5'-CAGCAAATATTAATCTGGAGACTCGG, 5'-GGGCGGCCAGTTCCA
CPSF-30	5'-CTGGAGCAAGTCACTTGCTACAAG, 5'-GCTGTCACTGCCCACTGAGA
Fip1	5'-ACAAGACACAAGTCCTCTCGCAG, 5'-TCTTTTCCTTCTTTGCTTCTTTTAGAC
WDR33	5'-CGGAAGACCGAGGAAGCAA, 5'-TCCGTCGTGATGGTCTCCAT
CstF-50	5'-CAAGGACGGCTGCATCAA, 5'-GCTACAGAGTCTTTTCCACTTGAGA
CstF-64	5'-GAGGACCCATGCTAGATCAGAGG, 5'-AAGCAGCCTTCTCATGATCC
CstF-64τ	5'-GTGATGAGAATCATGGATCCAGAGAT, 5'-AGATTTGCCTGGGATCAGTGG
CstF-77	5'-GAGGCCATGTCAGGAGAC, 5'-CATAAATCAATGTGCAAAACC
CFI-25	5'-CATTTCTAGTCTTCCTCAGCTGCTG, 5'-TGTAAGCTGTGCTCATAGAGACGG
CFI-68	5'-AAGTCCTATGGGTCTGGATCCAG, 5'-CTTTTGTCTTGGAAGGTGTTCCTC
CFI-59	5'-ATAGAGAACGGGACCGGCAC, 5'-TTTGCCTGGAGGAAGTTAGGG
Pcf11	5'-ACCATCCATCATGTTATGAAGATTATCA, 5'-TGCAATTCGTTTTTGACAATGTT
Clp1	5'-CCTATGTTGCTTTACCTCAACACTCA, 5'-ACCTTTGGTTGAACACATCTGCTAA
Symplekin	5'-CTGAAACCACACTGCTGGACTCTAG, 5'-CTTCTTCCACAGCATGGCTGA
PAPα	5'-CGGCTTCTCTGTTGGCCTC, 5'-TGGTTTTACCGATTCAGTCTCAGTT
PAPγ	5'-CCAACTATTGATACGGCACGC, 5'-GGTCAGGCGGATCGAGTTT
PABPN1	5'-CTACAGTGGTTTTAACAGCAGGCC, 5'-TTCCATCAAGGTCATCTTCTGTTTT
PABPC1	5'-GCCACTGGTGTTCCAACTGTCTA, 5'-TTAAGATATTTTTCTTCGGTGAAGCA
PP1α	5'-AGACCCTCATGTGTTCCTTCCA, 5'-TGGAGGCTATTTCTTGGCTTTG
PP1β	5'-TGAGACTTTGATGTGTTCATTCCAG, 5'-TTCACCTTTTCTTCGGTGGATT
RBBP6	5'-TCCACGCTTGTGGATTACACC, 5'-CAGCAGGAGCTGTGTTATCATTATTAT
U2AF65	5'-TTTGCCTTTTTGGAGTTCCG, 5'-TGGAAGATGATGCCATCAAA
SF3b155	5'-TGCTATTGGACCATGCAGAATG, 5'-GCATCCTGGGAGCCAATGTAG
U1-70K	5'-GGACCCTCACAATGATCCCA, 5'-TACACCTCAAACTCTCTCCG
RRP44	5'-TTACCACTAGTCTCCGTGGACTCA, 5'-CAGAGTCAAAGCCCCCTTTTC
RRP6	5'-TATGACCGAATGAGGCTGGAAC, 5'-CGAGTTCAGGTGCTTCTTTTGC
Timp2 proximal	5'-TTTCTTGACATCGAGGACCC, 5'-TCCAGGAAGGGATGTCAAAG
Timp2 distal	5'-ATGTGCGTGCTGGAATATGA, 5'-CTGATACAGAGCATCAGGCG
