# Supplementary material for: Systematic Profiling of Poly(A)+ Transcripts Modulated by Core 3’ End Processing and Splicing Factors Reveals Regulatory Rules of Alternative Cleavage and Polyadenylation
Source: PLoS Genet. 2015 Apr 23;11(4):e1005166. doi: 10.1371/journal.pgen.1005166 (PMC4407891; doi:10.1371/journal.pgen.1005166)
Supplement: S4 Table — (PDF) [file pgen.1005166.s017.pdf]

Table S4. Number of reads mapped to pAs in each sample

Sample	Replicate 1	Replicate 2	Replicate 3
siCPSF-160	3,538,673 (1)		
siCPSF-100	4,617,978 (3)		
siCPSF-73	3,568,061 (1)		
siCPSF-30	7,105,604 (3)		
siFip1	2,438,712 (2)	2,458,959 (8)	2,116,298 (9)
siWDR33	5,021,499 (4)		
siCstF-50	4,682,268 (4)		
siCstF-64	3,962,717 (1)		
siCstF-64τ	4,581,623 (4)		
siCstF-77	5,037,435 (5)		
siCFI-25	4,043,953 (1)		
siCFI-68	2,629,152 (1)	2,132,688 (8)	2,505,470 (9)
siCFI-59	3,729,298 (1)		
siPcf11	4,495,439 (4)	2,628,024 (8)	2,372,155 (9)
siClp1	5,482,199 (4)		
siSymplekin	5,242,727 (4)		
siPAPα	1,585,589 (6)		
siPAPγ	1,631,646 (6)		
siPABPN1	3,496,102 (3)	2,284,954 (8)	1,738,991 (9)
siPABPC1	5,157,454 (3)	2,912,024 (8)	1,835,084 (9)
siPP1α	4,638,723 (3)		
siPP1β	5,451,500 (3)		
siRBBP6	4,382,216 (4)		
siU2AF65	4,256,137 (7)		
siSF3b155	1,846,200 (7)		
siU1-70K	3,959,400 (7)		
siRRP44 + siRRP6	2,052,907 (6)		
U1D 8hr	2,054,949		
mU1D 8 hr	2,006,755		
U1D 24hr	2,042,368		
mU1D 24 hr	2,071,765		
siCtrl 1	2,309,371		
siCtrl 2	3,506,626		
siCtrl 3	3,440,068		
siCtrl 4	5,853,466		
siCtrl 5	4,642,375		
siCtrl 6	1,753,259		
siCtrl 7	6,659,829		
siCtrl 8 (nuclear RNA)	1,979,079		
siCtrl 9 (total RNA)	2,335,594		
Proliferating C2C12	1,829,374	1,781,818	
Differentiated C2C12	4,329,559	2,790,919	

The control siRNA (siCtrl) sample for each test sample is indicated in parenthesis.
