# Supplementary material for: Systematic Profiling of Poly(A)+ Transcripts Modulated by Core 3’ End Processing and Splicing Factors Reveals Regulatory Rules of Alternative Cleavage and Polyadenylation
Source: PLoS Genet. 2015 Apr 23;11(4):e1005166. doi: 10.1371/journal.pgen.1005166 (PMC4407891; doi:10.1371/journal.pgen.1005166)
Supplement: S5 Table — The data is also summarized in Fig 6B. Numbers are significance score (SS), which was calculated by –log10(P)*S, where P was based on the Fisher’s exact test and S = 1 for enrichment and -1 for depletion. (PDF) [file pgen.1005166.s018.pdf]

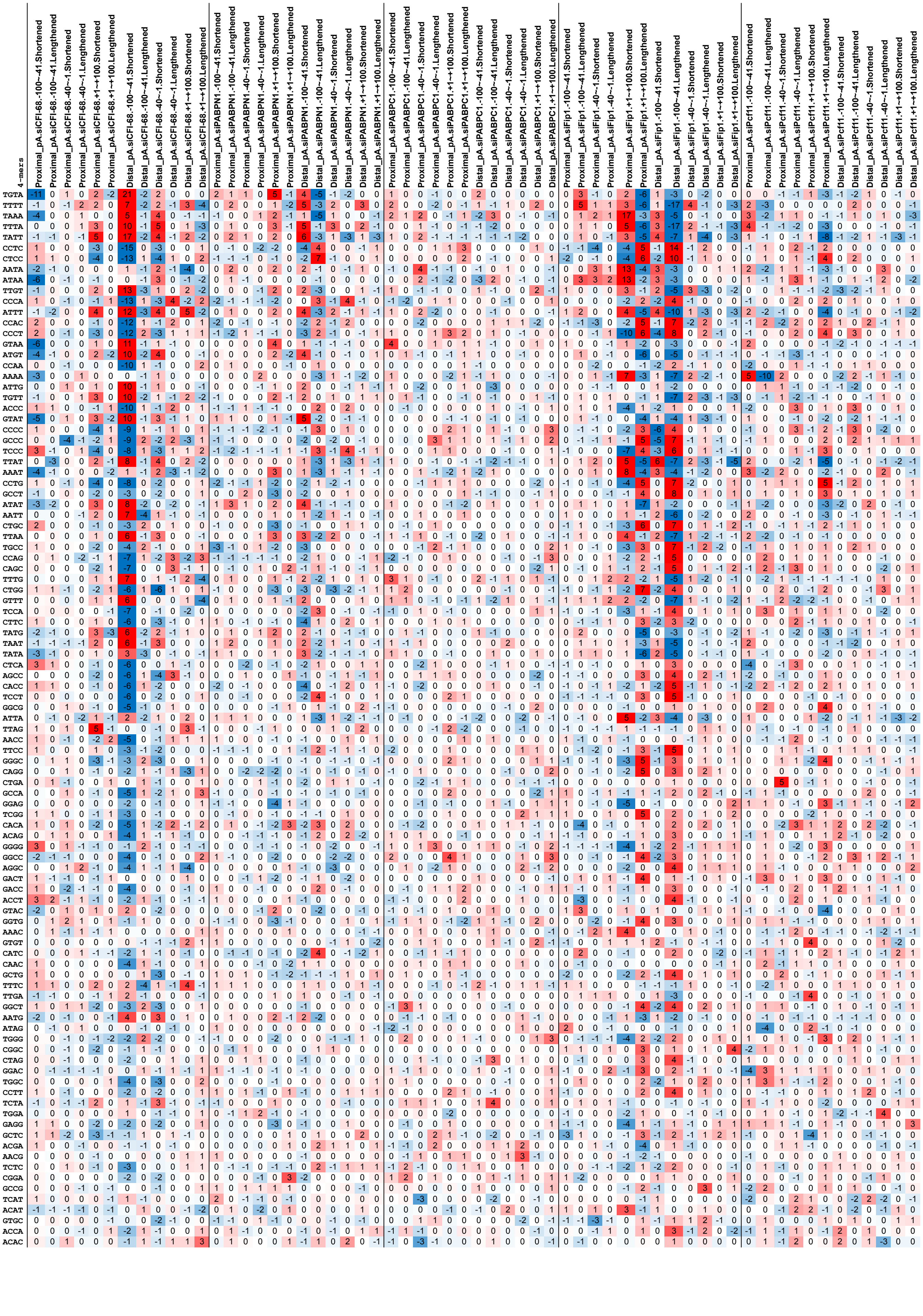
4-mers
Proximal_pA.siCFI-68.-100~-41.Shortened
Proximal_pA.siCFI-68.-100~-41.Lengthened
Proximal_pA.siCFI-68.-40~-1.Shortened
Proximal_pA.siCFI-68.-40~-1.Lengthened
Proximal_pA.siCFI-68.+1~+100.Shortened
Proximal_pA.siCFI-68.+1~+100.Lengthened
Distal_pA.siCFI-68.-100~-41.Shortened
Distal_pA.siCFI-68.-100~-41.Lengthened
Distal_pA.siCFI-68.-40~-1.Shortened
Distal_pA.siCFI-68.-40~-1.Lengthened
Distal_pA.siCFI-68.+1~+100.Shortened
Distal_pA.siCFI-68.+1~+100.Lengthened
Proximal_pA.siPABPN1.-100~-41.Shortened
Proximal_pA.siPABPN1.-100~-41.Lengthened
Proximal_pA.siPABPN1.-40~-1.Shortened
Proximal_pA.siPABPN1.-40~-1.Lengthened
Proximal_pA.siPABPN1.+1~+100.Shortened
Proximal_pA.siPABPN1.+1~+100.Lengthened
Distal_pA.siPABPN1.-100~-41.Shortened
Distal_pA.siPABPN1.-100~-41.Lengthened
Distal_pA.siPABPN1.-40~-1.Shortened
Distal_pA.siPABPN1.-40~-1.Lengthened
Distal_pA.siPABPN1.+1~+100.Shortened
Distal_pA.siPABPN1.+1~+100.Lengthened
Proximal_pA.siPABPC1.-100~-41.Shortened
Proximal_pA.siPABPC1.-100~-41.Lengthened
Proximal_pA.siPABPC1.-40~-1.Shortened
Proximal_pA.siPABPC1.-40~-1.Lengthened
Proximal_pA.siPABPC1.+1~+100.Shortened
Proximal_pA.siPABPC1.+1~+100.Lengthened
Distal_pA.siPABPC1.-100~-41.Shortened
Distal_pA.siPABPC1.-100~-41.Lengthened
Distal_pA.siPABPC1.-40~-1.Shortened
Distal_pA.siPABPC1.-40~-1.Lengthened
Distal_pA.siPABPC1.+1~+100.Shortened
Distal_pA.siPABPC1.+1~+100.Lengthened
Proximal_pA.siFip1.-100~-41.Shortened
Proximal_pA.siFip1.-100~-41.Lengthened
Proximal_pA.siFip1.-40~-1.Shortened
Proximal_pA.siFip1.-40~-1.Lengthened
Proximal_pA.siFip1.+1~+100.Shortened
Proximal_pA.siFip1.+1~+100.Lengthened
Distal_pA.siFip1.-100~-41.Shortened
Distal_pA.siFip1.-100~-41.Lengthened
Distal_pA.siFip1.-40~-1.Shortened
Distal_pA.siFip1.-40~-1.Lengthened
Distal_pA.siFip1.+1~+100.Shortened
Distal_pA.siFip1.+1~+100.Lengthened
Proximal_pA.siPcf11.-100~-41.Shortened
Proximal_pA.siPcf11.-100~-41.Lengthened
Proximal_pA.siPcf11.-40~-1.Shortened
Proximal_pA.siPcf11.-40~-1.Lengthened
Proximal_pA.siPcf11.+1~+100.Shortened
Proximal_pA.siPcf11.+1~+100.Lengthened
Distal_pA.siPcf11.-100~-41.Shortened
Distal_pA.siPcf11.-100~-41.Lengthened
Distal_pA.siPcf11.-40~-1.Shortened
Distal_pA.siPcf11.-40~-1.Lengthened
Distal_pA.siPcf11.+1~+100.Shortened
Distal_pA.siPcf11.+1~+100.Lengthened
TGTA
TTTT
TAAA
TTTA
TATT
CCTC
CTCC
AATA
ATAA
TTGT
CCCA
ATTT
CCAC
CCCT
GTAA
ATGT
CCAA
AAAA
ATTG
TGTT
ACCC
GTAT
CCCC
GCCC
TCCC
TTAT
AAAT
CCTG
GCCT
ATAT
AATT
CTGC
TTAA
TGCC
CCAG
CAGC
TTTG
CTGG
GTTT
TCCA
CTTC
TATG
TAAT
TATA
CTCA
AGCC
CACC
TCCT
GGCG
ATTA
TTAG
AACC
TTCC
GGGC
CAGG
CTGA
GCCA
GGAG
TCGG
CACA
ACAG
GGGG
GGCC
AGGC
GACT
GACC
ACCT
GTAC
GGTG
AAAC
GTGT
CATC
CAAC
GCTG
TTTC
TTGA
GGCT
AATG
ATAG
TGGG
CGGC
CTAG
GGAC
TGGC
CCTT
TCTA
TGGA
GAGG
GCTC
ACGA
AACG
TCTC
CGGA
GCCG
TCAT
ACAT
GTGC
ACCA
ACAC

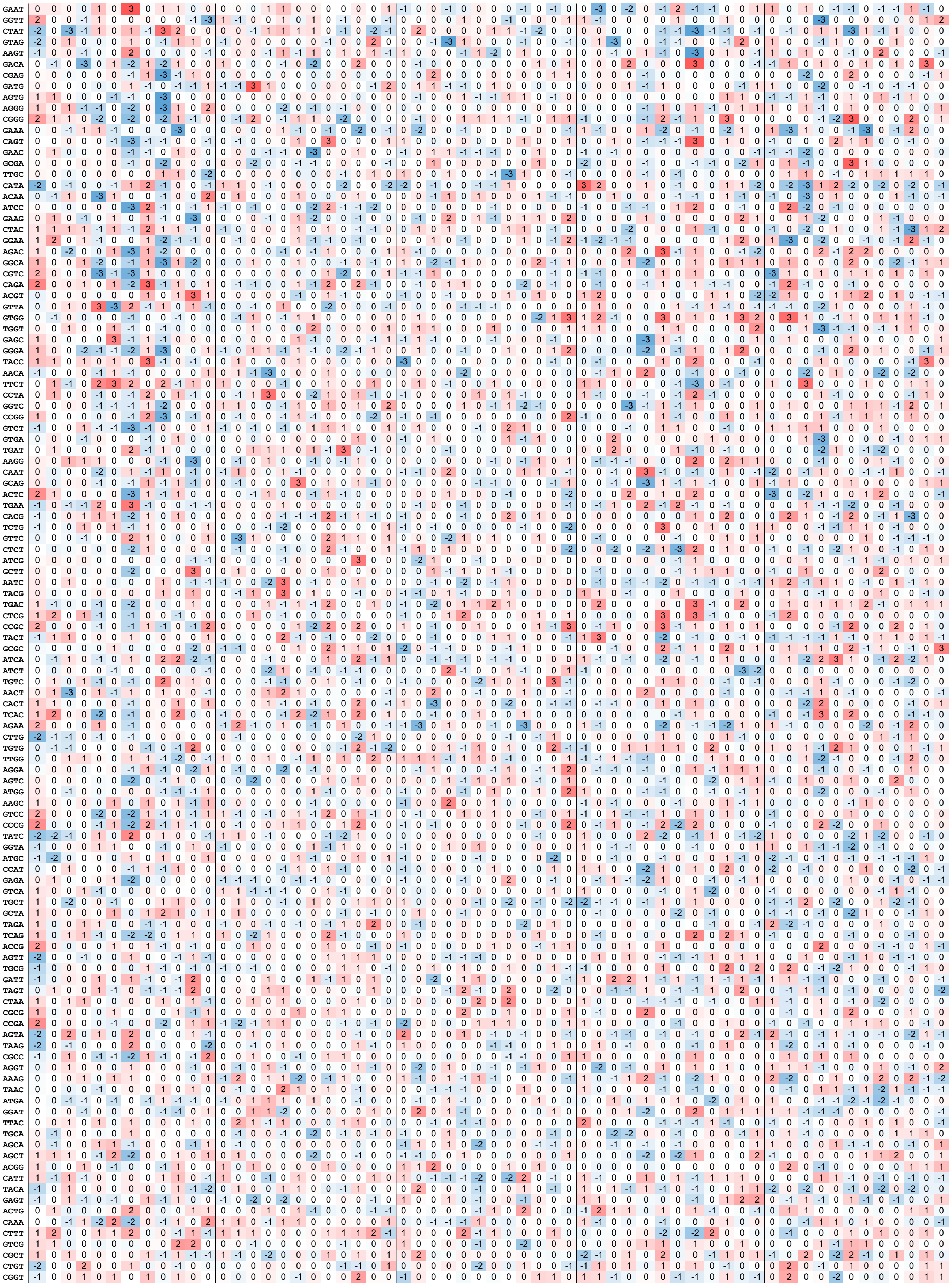
GAAT
GGTT
CTAT
GTAG
AAGT
GACA
CGAG
GATG
AGTG
AGGG
CGGG
GAAA
CAGT
GAAC
GCGA
TTGC
CATA
ACAA
ATCC
GAAG
CTAC
GGAA
AGAC
GGCA
CGTC
CAGA
ACGT
GTTA
GTGG
TGGT
GAGC
GGGA
TACC
AACA
TTCT
CCTA
GGTC
CCGG
GTCT
GTGA
TGAT
AAGG
CAAT
GCAG
ACTC
TGAA
CACG
TCTG
GTTC
CTCT
ATCG
GCTT
AATC
TACG
TGAC
CTCG
CCGC
TACT
GCGC
ATCA
ATCT
TGTC
AACT
CACT
TCAC
AGAA
CTTG
TGTG
TTGG
AGGA
AGTC
ATGG
AAGC
GTCC
CCCG
TATC
GGTA
ATGC
CCAT
GAGA
GTCA
TGCT
GCTA
TAGA
TCAG
ACCG
AGTT
TGCG
GATT
TAGT
CTAA
CGCG
CCGA
AGTA
TAAG
CGCC
AGGT
AAAG
TAAC
ATGA
GGAT
TTAC
TGCA
AGCA
AGCT
ACGG
CATT
TACA
GAGT
ACTG
CAAA
CTTT
GTCG
CGCT
CTGT
CGGT

| | | | | | | | | | | | | | | | | | | | | | | | | | | | | | | | | | | | | | | | | | | | | | | | | | | | | | | | | | | | | |
|---|---|---|---|---|---|---|---|---|---|---|---|---|---|---|---|---|---|---|---|---|---|---|---|---|---|---|---|---|---|---|---|---|---|---|---|---|---|---|---|---|---|---|---|---|---|---|---|---|---|---|---|---|---|---|---|---|---|---|---|---|
| TGAG | 0 | 0 | 0 | 0 | -1 | 1 | -1 | 0 | 0 | 1 | 0 | 1 | 0 | -1 | -1 | 0 | 0 | -1 | 0 | -1 | 0 | 1 | -2 | -1 | 0 | 0 | -1 | 1 | 0 | -1 | 0 | 0 | 0 | 2 | -1 | 0 | 0 | -1 | 1 | 0 | -1 | 1 | 0 | -1 | -2 | 1 | 0 | 1 | 0 | 0 | 2 | -1 | 0 | 1 | -1 | 0 | -1 | 0 | 0 | 0 |
| CGTG | 0 | 0 | 0 | -1 | 0 | 0 | 0 | 0 | 0 | 0 | 0 | 0 | 0 | -1 | 1 | 0 | 1 | 0 | 0 | 0 | 1 | 0 | 0 | 0 | 0 | 0 | 0 | 1 | 1 | -1 | 0 | 0 | -1 | -1 | 0 | 0 | -1 | 0 | -1 | 0 | 0 | 0 | -1 | 1 | 1 | 1 | 0 | -1 | -1 | 2 | 0 | 0 | 0 | -1 | 1 | 0 | 0 | 0 | 0 | 0 |
| ACTA | 0 | -1 | 0 | 1 | -1 | 0 | 0 | 0 | 1 | 1 | 1 | 2 | 1 | 0 | 0 | 0 | 1 | 0 | 0 | 0 | -1 | 0 | -1 | 0 | 0 | 0 | -1 | 0 | 0 | 0 | 0 | 2 | 0 | 0 | -1 | -1 | 1 | 0 | 0 | -1 | 1 | 0 | 0 | 0 | -2 | 0 | 1 | 0 | 0 | 0 | -1 | 1 | 1 | 1 | 0 | -1 | 0 | -1 | 0 | 0 |
| AGCG | 0 | 0 | -2 | 0 | 0 | 1 | 0 | 0 | -1 | 1 | 0 | 0 | 0 | 0 | 0 | 0 | 0 | 0 | -2 | 0 | 1 | 0 | 0 | 0 | -1 | 0 | 0 | 0 | 0 | 0 | 0 | 1 | 0 | 1 | -1 | 0 | 0 | 0 | 0 | 0 | -1 | 0 | 0 | -1 | 0 | 2 | 0 | 1 | 0 | 1 | -1 | 1 | 0 | 1 | 0 | 0 | 0 | 0 | 0 | 0 |
| CAAG | 0 | -1 | 1 | 0 | 0 | -1 | -1 | 2 | -2 | -1 | -1 | 0 | 0 | 0 | 0 | 0 | -1 | 0 | -1 | 0 | 0 | 0 | 0 | 0 | -1 | 1 | 0 | 0 | -1 | 0 | 0 | 0 | -1 | 0 | -1 | 0 | -1 | 0 | -1 | 0 | 0 | -1 | -2 | 2 | 0 | 1 | 0 | 0 | -1 | 0 | 0 | 0 | -1 | 0 | 1 | 0 | 0 | -1 | 1 | 0 |
| TCAA | 0 | 0 | 0 | 0 | 0 | -1 | 0 | 1 | 0 | 0 | -1 | -1 | 0 | 0 | 0 | -1 | -1 | 0 | 0 | 1 | 0 | 1 | 1 | 1 | -1 | -1 | 0 | -1 | 0 | 0 | 0 | -1 | 0 | 0 | 1 | -1 | 0 | -1 | -2 | 0 | 0 | -2 | 0 | 0 | -1 | 1 | 0 | 0 | 0 | 0 | -1 | 1 | 1 | 0 | 1 | 0 | 0 | 0 | 1 | -1 |
| TTCG | 0 | 1 | 0 | 0 | 0 | 0 | 0 | 0 | 0 | 0 | 0 | 0 | 1 | 0 | 0 | 1 | 0 | 0 | -1 | -1 | 0 | 2 | 0 | 0 | 0 | 0 | 0 | 1 | 1 | 0 | 1 | 0 | 0 | 0 | 0 | 0 | 0 | 0 | 1 | 1 | 0 | 1 | 0 | 1 | -1 | 0 | -1 | 1 | 0 | 0 | 0 | 0 | 0 | 0 | 1 | 0 | -1 | 0 | -1 | 1 |
| GATC | 0 | 0 | 0 | 0 | -1 | 0 | -1 | 0 | -1 | 1 | -1 | 0 | 0 | 0 | 0 | 1 | 0 | 0 | -1 | 0 | 0 | 0 | -1 | 0 | -1 | 0 | 0 | 0 | 0 | 0 | 0 | 0 | -1 | 0 | 1 | 1 | 0 | -1 | 1 | 0 | 0 | 1 | 1 | 2 | 1 | 2 | 0 | 0 | -1 | 0 | 0 | 0 | 0 | 0 | 0 | 0 | 0 | 0 | 0 | -1 |
| GCGT | 0 | 0 | -1 | 0 | 0 | 0 | -2 | 0 | 0 | 0 | 0 | 1 | 0 | 0 | 0 | 0 | 0 | 0 | -1 | 0 | 0 | 0 | 0 | 0 | -1 | 0 | 1 | 1 | 0 | 0 | 0 | 1 | 0 | 0 | 0 | -1 | -2 | -1 | 0 | 0 | 0 | 0 | 0 | 0 | 0 | 2 | 1 | 1 | 0 | 1 | 0 | 0 | -1 | 0 | 1 | 0 | 1 | 0 | 1 | -1 |
| CGCA | 1 | 0 | -1 | 0 | 0 | 1 | -1 | 0 | 0 | 1 | -1 | 0 | 1 | 0 | 1 | 0 | 0 | 1 | 0 | 1 | 0 | -1 | -1 | 1 | 0 | -1 | 0 | 0 | 0 | 0 | 0 | 0 | 0 | 0 | -1 | 1 | 1 | 0 | 1 | 0 | -1 | 2 | -1 | 1 | 0 | 1 | 0 | 0 | 0 | -1 | 0 | 1 | 0 | 0 | 1 | 0 | 1 | 0 | 1 | 2 |
| TCGT | 1 | 1 | 0 | 0 | -1 | 0 | 0 | 0 | 1 | 0 | 1 | 0 | 0 | 0 | 0 | 0 | 0 | 0 | 0 | 0 | -1 | 1 | 0 | 0 | 0 | 0 | 0 | 0 | 1 | -1 | 0 | 0 | 0 | -1 | 0 | 0 | -2 | 0 | 0 | 0 | 0 | 0 | 0 | 1 | -1 | 0 | 0 | -1 | 0 | 1 | 0 | 0 | 0 | 0 | 1 | 1 | 0 | 0 | 0 | 1 |
| TCCG | 2 | 0 | 1 | 0 | -1 | -1 | 0 | 2 | -1 | -1 | -2 | 1 | 0 | -1 | 0 | 0 | 0 | 0 | -2 | 2 | -1 | 0 | -1 | 1 | 0 | 0 | 0 | -1 | 0 | 1 | -1 | 1 | -1 | 0 | -1 | 1 | 0 | -1 | 0 | -1 | 1 | 0 | 0 | 2 | 0 | 0 | 1 | 1 | -1 | 0 | -1 | 0 | 0 | 0 | 1 | 0 | 1 | 1 | 0 | 0 |
| CGTA | -1 | 0 | 0 | 1 | 0 | 1 | 0 | 0 | 0 | 2 | 0 | 0 | -1 | 0 | -1 | 0 | 1 | 1 | 1 | 1 | -1 | 0 | -1 | 0 | 0 | -1 | 0 | 0 | 0 | 0 | 0 | 0 | 0 | 0 | 0 | 0 | 0 | 1 | 0 | 1 | 0 | -1 | 0 | 0 | 0 | 0 | -1 | 0 | 0 | 0 | -1 | 1 | 0 | 0 | 1 | 1 | 0 | 0 | 0 | -1 |
| AAGA | 0 | 0 | 0 | -1 | 0 | 0 | 0 | 1 | 0 | -1 | 0 | 0 | -1 | 2 | -1 | 0 | 0 | 0 | -2 | 0 | 1 | -1 | -1 | 1 | 1 | -2 | 0 | 0 | 0 | 1 | -2 | 0 | 0 | 0 | 0 | 0 | 0 | 0 | 1 | 2 | 1 | -1 | 0 | -1 | 0 | 0 | 0 | 0 | 0 | -1 | 0 | 0 | 0 | 1 | -1 | 0 | 0 | 2 | 0 | 0 |
| AGAG | 1 | 1 | 0 | 0 | 0 | 0 | -1 | 1 | -1 | 0 | -1 | 0 | 0 | 0 | -1 | 0 | -2 | 0 | -1 | 0 | 0 | 0 | 0 | 1 | 0 | -1 | 1 | 0 | 0 | 0 | 0 | 1 | 0 | -1 | -2 | 0 | 0 | 0 | 0 | 0 | -2 | 2 | 0 | 1 | 0 | 0 | 0 | 1 | 0 | 0 | 0 | 0 | -1 | 0 | 0 | 1 | -1 | 0 | 0 | 1 |
| CGAC | 1 | 1 | 0 | 0 | -1 | 0 | 0 | -1 | 0 | 0 | 0 | 2 | 1 | 0 | 0 | 0 | 0 | 0 | 0 | 2 | 0 | 1 | 0 | -1 | 0 | 0 | 0 | 0 | 0 | 1 | 0 | -1 | 0 | 2 | 0 | 0 | 0 | 0 | 2 | 0 | -1 | -1 | 0 | 2 | -1 | 0 | -1 | 0 | 0 | 0 | 0 | 0 | 0 | 0 | 0 | 0 | 0 | -1 | 0 | 0 |
| CGAA | 1 | 1 | 0 | 1 | 0 | 0 | 0 | -1 | -1 | 0 | 0 | 0 | -1 | 0 | -2 | 0 | 0 | 0 | 0 | -1 | 0 | 1 | -1 | 1 | -2 | -1 | -1 | 0 | 0 | 0 | 0 | 0 | 0 | 1 | 0 | -1 | 0 | 0 | -1 | 0 | 1 | -1 | 0 | 0 | -1 | 0 | 0 | 0 | 0 | 0 | 0 | 2 | -1 | 0 | 0 | 0 | 0 | 0 | 0 | 0 |
| CTTA | 2 | 0 | 0 | -1 | 1 | 0 | 0 | 0 | 0 | 1 | 1 | 1 | 0 | 1 | 1 | 0 | 1 | 1 | 1 | 0 | 0 | 0 | 1 | 0 | 1 | 0 | 0 | 0 | 0 | 0 | 0 | 0 | -1 | 0 | 0 | 0 | 0 | 0 | 0 | -1 | 0 | 0 | 0 | 0 | 0 | 0 | 0 | -1 | -1 | -1 | 0 | 0 | 0 | -1 | 0 | 0 | 2 | 0 | -1 | 1 |
| GCGG | -1 | 0 | 0 | 0 | -1 | 0 | -1 | 0 | 0 | 1 | 0 | -1 | 0 | -1 | 1 | 0 | 0 | 2 | 0 | 0 | 0 | 0 | 1 | 0 | 0 | 1 | 0 | 0 | 1 | 0 | 0 | 1 | 0 | 1 | 2 | 1 | 2 | 0 | 0 | -1 | -1 | 1 | 0 | 0 | 1 | 1 | 0 | 0 | -1 | 2 | 0 | 0 | -1 | 2 | -1 | 0 | -1 | 0 | 0 | 0 |
| ATAC | 0 | 0 | 0 | 0 | 0 | 0 | 0 | 1 | 0 | 0 | -1 | 0 | 1 | 0 | 0 | 0 | 1 | 0 | 0 | 0 | -1 | 0 | 0 | 0 | -1 | 1 | 0 | 0 | 0 | -1 | -1 | 0 | 0 | 0 | 0 | -1 | 0 | -1 | 2 | 0 | 0 | -1 | 1 | 0 | -1 | 0 | 0 | 0 | 0 | 0 | -1 | 0 | 0 | -1 | 0 | -1 | 1 | 0 | 0 | -1 |
| ACGC | 0 | -1 | 1 | 0 | 1 | 0 | -1 | 1 | -1 | 1 | -2 | 0 | 0 | -1 | 0 | 0 | 0 | 0 | -1 | 0 | 0 | -1 | 0 | 0 | 1 | 0 | 0 | -1 | 0 | 0 | 1 | 1 | 0 | 1 | 0 | 1 | 0 | 1 | 0 | 0 | -1 | 1 | -1 | 1 | -1 | 0 | 1 | 0 | 0 | 0 | 0 | 0 | 0 | 1 | 0 | 0 | 0 | -1 | 1 | 1 |
| TAGG | 0 | 0 | 1 | -1 | 0 | -1 | 0 | 1 | 0 | 1 | 0 | 0 | 1 | -1 | 1 | 1 | 0 | 1 | 0 | -1 | 0 | 0 | 0 | -1 | -1 | 0 | 0 | 0 | 0 | 0 | 0 | 0 | 0 | 0 | 0 | 0 | 0 | -1 | -1 | 0 | 0 | 0 | 0 | 1 | -2 | 0 | 0 | 0 | 1 | 0 | 0 | -1 | 0 | -1 | 0 | 1 | 0 | 0 | 0 | 1 |
| TCGA | 0 | 1 | 0 | 1 | 0 | 0 | 0 | 0 | 0 | 0 | 0 | 0 | 0 | 2 | -1 | 1 | 0 | 0 | -1 | -1 | 0 | 2 | -1 | 0 | 0 | -2 | 0 | 0 | 0 | 0 | 0 | 0 | 0 | 0 | 1 | 0 | 0 | 0 | -1 | 0 | 0 | 0 | 0 | 1 | 0 | -1 | 0 | -1 | 0 | 1 | 0 | 0 | 0 | 0 | 0 | 0 | 1 | 0 | -1 | 0 |
| TCTT | 0 | 0 | 0 | 0 | 1 | 0 | 1 | 0 | 0 | 0 | 0 | 0 | 2 | 0 | 0 | 0 | -1 | 0 | 0 | 1 | 0 | 0 | 2 | 0 | 0 | 1 | 0 | 1 | 0 | -1 | 0 | 0 | 0 | 0 | 1 | 0 | 1 | 0 | -1 | 0 | 1 | -1 | 0 | -1 | 0 | -1 | 0 | -1 | -1 | 0 | 1 | 0 | 0 | 0 | 0 | 1 | 0 | 1 | 1 | 0 |
| CCGT | 1 | 0 | 0 | 0 | -1 | 0 | 0 | 1 | 0 | 1 | -2 | 0 | 1 | 0 | 1 | 0 | 2 | 0 | 0 | 1 | 0 | 1 | 0 | 1 | 1 | 0 | 1 | -1 | 0 | 0 | 0 | 0 | -1 | -2 | 0 | 1 | 0 | -1 | 0 | 0 | 0 | 1 | 0 | 1 | 0 | 1 | 0 | 0 | -1 | 0 | 0 | 1 | 0 | -1 | 1 | 0 | 0 | 0 | 0 | 0 |
| GGGT | 1 | 0 | 0 | 0 | 0 | 0 | -2 | 1 | -2 | 1 | 0 | 0 | 0 | 0 | -1 | -1 | -2 | 0 | 0 | 1 | -1 | 0 | 0 | 0 | -1 | 1 | 1 | 0 | 0 | 0 | 0 | 0 | -1 | 0 | 0 | 1 | 0 | 0 | 0 | -1 | -1 | 1 | -1 | 1 | 0 | 0 | 1 | 1 | 1 | 1 | 1 | -2 | 0 | 1 | 0 | 1 | 0 | 2 | 0 | 0 |
| CGTT | 1 | 0 | 0 | 0 | 0 | 0 | 0 | 1 | 0 | 0 | 1 | 0 | 1 | 0 | 1 | 0 | 0 | 0 | -1 | 0 | 0 | 0 | 0 | 0 | 0 | 0 | 0 | 0 | 0 | 0 | 0 | 0 | 1 | 0 | 0 | 0 | 0 | 0 | 2 | 0 | 1 | 0 | 0 | 1 | 0 | 0 | 1 | 0 | 0 | 1 | 0 | 0 | 0 | 0 | 1 | 0 | 0 | 0 | -1 | 0 |
| TAGC | 0 | 0 | 0 | -1 | 1 | 0 | -1 | 0 | -1 | 0 | 0 | 1 | 0 | 2 | 0 | 0 | -1 | 0 | 0 | 0 | 1 | -1 | 0 | 0 | 0 | -1 | 0 | -1 | 1 | -1 | 0 | 0 | 0 | 0 | -1 | 0 | 0 | 0 | 0 | 0 | -1 | 0 | -1 | 1 | 0 | 1 | 1 | 0 | 1 | -1 | 0 | -1 | 1 | 0 | -1 | 0 | 1 | 0 | 0 | 1 |
| GCAC | 0 | 0 | 0 | 0 | 0 | 0 | -1 | 1 | -1 | 0 | -1 | 0 | 1 | 0 | 1 | 1 | -1 | -1 | -2 | 0 | 0 | 0 | 0 | 0 | 0 | 0 | -1 | 0 | 1 | 0 | 0 | 0 | 0 | 0 | 0 | 0 | 0 | 0 | 0 | 0 | -2 | 0 | -1 | 1 | 0 | 1 | 0 | 1 | 0 | 1 | 0 | 1 | 1 | 0 | 0 | 0 | 1 | -1 | 1 | 1 |
| TCGC | 0 | 0 | 0 | 0 | 0 | 0 | 0 | 0 | -1 | 0 | 0 | 0 | 2 | 1 | 0 | -1 | 0 | 0 | 0 | 0 | 0 | 0 | 0 | 0 | 0 | 0 | 0 | 1 | 1 | -1 | 0 | 0 | -1 | 0 | 0 | 1 | 0 | 0 | 1 | 0 | 0 | 0 | 0 | 1 | 0 | -1 | -1 | 0 | -1 | -1 | 0 | 1 | 0 | 0 | 1 | -1 | 0 | 2 | 0 | 0 |
| TTCA | 1 | -1 | 1 | 0 | 1 | -1 | 1 | -1 | 0 | -1 | 2 | 0 | 2 | 0 | 0 | 0 | 0 | 0 | 0 | 1 | 0 | 0 | 1 | 0 | 1 | 0 | -1 | 0 | 0 | 0 | 0 | -1 | 0 | 0 | -1 | -1 | 0 | -1 | 0 | 0 | 0 | -1 | 0 | 0 | 0 | 1 | 1 | 0 | -1 | 0 | -1 | 1 | 1 | 0 | 0 | 0 | 0 | -1 | 0 | -1 |
| GCAT | 1 | -1 | 0 | 1 | 0 | 0 | 0 | 0 | -1 | 0 | -1 | 0 | 0 | -2 | 0 | 0 | 0 | 0 | -1 | 0 | -1 | -1 | 0 | 0 | 0 | 0 | -2 | 0 | 0 | -1 | 0 | 0 | 1 | 0 | -1 | 0 | 1 | 1 | -2 | -1 | 0 | 0 | -1 | 0 | 0 | 1 | 0 | 1 | 1 | 0 | -1 | 0 | 0 | 0 | -1 | -1 | -1 | 0 | 1 | 0 |
| AGAT | 0 | -1 | 1 | 1 | 0 | 0 | 1 | 0 | 0 | 0 | 0 | 0 | -1 | -1 | 0 | 0 | -1 | -1 | 0 | 0 | 0 | 2 | 0 | 0 | -1 | 0 | 0 | -1 | 0 | 0 | 0 | 0 | 0 | 0 | 0 | 0 | -1 | 0 | 1 | 0 | 0 | -1 | 0 | -1 | 0 | 1 | 0 | 0 | 0 | -1 | -1 | -1 | 1 | -1 | 0 | 0 | 0 | -1 | 0 | -1 |
| ATTC | 0 | 0 | 1 | 0 | 1 | 0 | 1 | 0 | 0 | 0 | -1 | 0 | 1 | 0 | -1 | 0 | 0 | 1 | 0 | 0 | 0 | 0 | 1 | 0 | 0 | 0 | 0 | 0 | 0 | -1 | -1 | 0 | -1 | 0 | 0 | 0 | 1 | 0 | 0 | 0 | 0 | 0 | 0 | 0 | 0 | 0 | 1 | 0 | 0 | 1 | 0 | 0 | 0 | -2 | 0 | -1 | 0 | 0 | 0 | 0 |
| ACTT | 0 | 1 | -1 | 0 | 1 | 0 | 1 | -1 | 1 | 0 | 1 | -1 | 0 | 0 | 0 | 0 | 1 | -1 | 0 | 0 | 0 | 0 | 0 | -1 | 0 | 0 | 0 | 0 | 0 | 0 | 1 | 0 | -1 | 0 | 0 | -1 | 0 | 0 | 0 | 1 | 0 | 0 | 0 | -2 | 0 | 0 | 0 | 0 | 0 | 0 | -1 | 1 | 0 | 0 | 0 | 0 | 0 | 0 | 0 | 1 |
| CGAT | 1 | 0 | 0 | 0 | 1 | 0 | 0 | 0 | 0 | 0 | 0 | -1 | 0 | 1 | -1 | 1 | 0 | 0 | -1 | 0 | 0 | 0 | -1 | 1 | -1 | 0 | 0 | 1 | 0 | 1 | 1 | 1 | 0 | 1 | 1 | 0 | 0 | 0 | 0 | 0 | 0 | 0 | 0 | 0 | 0 | 0 | 0 | 0 | 0 | 2 | 0 | 0 | 0 | 0 | 1 | 0 | 0 | 0 | -1 | -1 |
| GCAA | 1 | 0 | 0 | 0 | 0 | 1 | 0 | 0 | 1 | 0 | -1 | 0 | 0 | 0 | 0 | 0 | 1 | -1 | 0 | -1 | 0 | 0 | 0 | 0 | -1 | 0 | 1 | 1 | 0 | 0 | 0 | 0 | 0 | 1 | -1 | 0 | 0 | 0 | 0 | 0 | 1 | 0 | 0 | 0 | 2 | 0 | 1 | 1 | 0 | 0 | 0 | 0 | 0 | 0 | 0 | 0 | 0 | -1 | 0 | 0 |
| CATG | 0 | 0 | 1 | 0 | 0 | 0 | 0 | -1 | 0 | -1 | -1 | 0 | 0 | 0 | 0 | 0 | -1 | -1 | 1 | 0 | -1 | 0 | 1 | 0 | -1 | 0 | 0 | 1 | 0 | 1 | 0 | 0 | 0 | 0 | 0 | 0 | 0 | 0 | 1 | 0 | 0 | -1 | 0 | 0 | 0 | 0 | 0 | 1 | -1 | 1 | -1 | 0 | 0 | 0 | 0 | -1 | 0 | -1 | 1 | 0 |
| GTTG | 0 | 0 | 0 | 0 | 0 | 0 | 1 | -1 | 0 | 0 | 0 | -1 | 0 | 0 | 1 | 0 | 1 | 0 | 1 | 0 | 1 | 1 | 0 | 0 | 0 | 0 | -1 | 0 | -1 | 0 | 0 | 0 | -1 | 0 | 0 | 0 | 0 | -1 | 0 | 0 | 0 | 0 | 0 | -1 | 1 | 0 | 0 | 0 | 0 | 0 | 0 | -1 | 0 | 0 | 0 | 0 | -1 | 1 | 1 | 0 |
| GATA | 0 | 0 | 0 | 0 | 1 | 0 | 1 | -1 | 0 | -1 | 0 | 0 | 0 | 0 | 0 | 0 | -1 | 0 | 0 | 0 | 0 | 0 | 0 | 0 | 0 | 0 | 1 | 0 | 0 | 0 | 0 | 0 | 0 | 0 | 0 | 0 | 0 | 0 | 0 | -1 | -1 | -1 | 0 | -1 | -1 | 0 | 0 | 0 | 0 | 0 | 0 | -1 | 1 | 0 | 0 | -1 | 0 | -1 | -1 | 0 |
| GACG | 1 | 0 | 0 | 1 | 0 | -1 | 0 | 1 | -1 | 0 | 0 | 0 | 0 | -1 | 0 | 0 | -1 | 1 | 0 | 1 | -1 | 0 | 0 | 0 | 1 | 0 | 0 | 0 | 0 | -1 | 1 | 0 | 0 | 0 | 0 | 0 | 1 | 0 | 0 | 0 | 0 | 0 | 0 | 0 | -1 | 0 | 1 | -1 | 0 | 1 | 0 | 0 | 0 | 0 | 0 | -1 | 1 | 0 | 0 | 0 |
