# Supplementary material for: Systematic Profiling of Poly(A)+ Transcripts Modulated by Core 3’ End Processing and Splicing Factors Reveals Regulatory Rules of Alternative Cleavage and Polyadenylation
Source: PLoS Genet. 2015 Apr 23;11(4):e1005166. doi: 10.1371/journal.pgen.1005166 (PMC4407891; doi:10.1371/journal.pgen.1005166)
Supplement: S6 Table — Only those with P≤0.001 (Fisher’s exact test) in any one of the comparisons are shown. Numbers are significance score (SS), which was calculated by –log10(P)*S, where P was based on the Fisher’s exact test and S = 1 for enrichment and -1 for depletion. (PDF) [file pgen.1005166.s019.pdf]

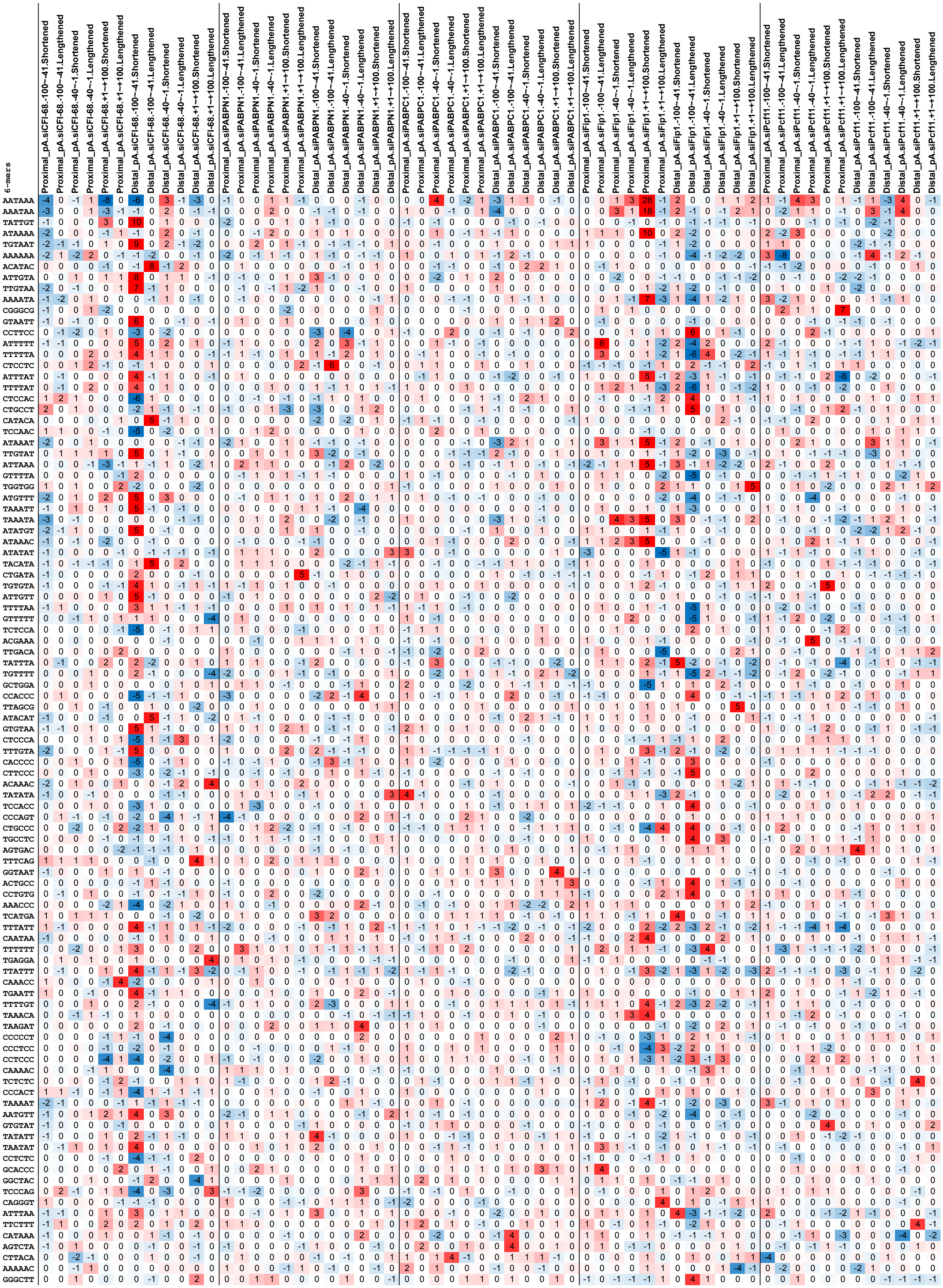
6-mers
Proximal_pA.siCFI-68.-100~-41.Shortened
Proximal_pA.siCFI-68.-100~-41.Lengthened
Proximal_pA.siCFI-68.-40~-1.Shortened
Proximal_pA.siCFI-68.-40~-1.Lengthened
Proximal_pA.siCFI-68.+1~+100.Shortened
Proximal_pA.siCFI-68.+1~+100.Lengthened
Distal_pA.siCFI-68.-100~-41.Shortened
Distal_pA.siCFI-68.-100~-41.Lengthened
Distal_pA.siCFI-68.-40~-1.Shortened
Distal_pA.siCFI-68.-40~-1.Lengthened
Distal_pA.siCFI-68.+1~+100.Shortened
Distal_pA.siCFI-68.+1~+100.Lengthened
Proximal_pA.siPABPN1.-100~-41.Shortened
Proximal_pA.siPABPN1.-100~-41.Lengthened
Proximal_pA.siPABPN1.-40~-1.Shortened
Proximal_pA.siPABPN1.-40~-1.Lengthened
Proximal_pA.siPABPN1.+1~+100.Shortened
Proximal_pA.siPABPN1.+1~+100.Lengthened
Distal_pA.siPABPN1.-100~-41.Shortened
Distal_pA.siPABPN1.-100~-41.Lengthened
Distal_pA.siPABPN1.-40~-1.Shortened
Distal_pA.siPABPN1.-40~-1.Lengthened
Distal_pA.siPABPN1.+1~+100.Shortened
Distal_pA.siPABPN1.+1~+100.Lengthened
Proximal_pA.siPABPC1.-100~-41.Shortened
Proximal_pA.siPABPC1.-100~-41.Lengthened
Proximal_pA.siPABPC1.-40~-1.Shortened
Proximal_pA.siPABPC1.-40~-1.Lengthened
Proximal_pA.siPABPC1.+1~+100.Shortened
Proximal_pA.siPABPC1.+1~+100.Lengthened
Distal_pA.siPABPC1.-100~-41.Shortened
Distal_pA.siPABPC1.-100~-41.Lengthened
Distal_pA.siPABPC1.-40~-1.Shortened
Distal_pA.siPABPC1.-40~-1.Lengthened
Distal_pA.siPABPC1.+1~+100.Shortened
Distal_pA.siPABPC1.+1~+100.Lengthened
Proximal_pA.siFip1.-100~-41.Shortened
Proximal_pA.siFip1.-100~-41.Lengthened
Proximal_pA.siFip1.-40~-1.Shortened
Proximal_pA.siFip1.-40~-1.Lengthened
Proximal_pA.siFip1.+1~+100.Shortened
Proximal_pA.siFip1.+1~+100.Lengthened
Distal_pA.siFip1.-100~-41.Shortened
Distal_pA.siFip1.-100~-41.Lengthened
Distal_pA.siFip1.-40~-1.Shortened
Distal_pA.siFip1.-40~-1.Lengthened
Distal_pA.siFip1.+1~+100.Shortened
Distal_pA.siFip1.+1~+100.Lengthened
Proximal_pA.siPcf11.-100~-41.Shortened
Proximal_pA.siPcf11.-100~-41.Lengthened
Proximal_pA.siPcf11.-40~-1.Shortened
Proximal_pA.siPcf11.-40~-1.Lengthened
Proximal_pA.siPcf11.+1~+100.Shortened
Proximal_pA.siPcf11.+1~+100.Lengthened
Distal_pA.siPcf11.-100~-41.Shortened
Distal_pA.siPcf11.-100~-41.Lengthened
Distal_pA.siPcf11.-40~-1.Shortened
Distal_pA.siPcf11.-40~-1.Lengthened
Distal_pA.siPcf11.+1~+100.Shortened
Distal_pA.siPcf11.+1~+100.Lengthened
AATAAA
AAATAA
TATTGT
ATAAAA
TGTAAT
AAAAAA
ACATAC
ATTGTA
TTGTAA
AAAATA
CGGGCG
GTAATT
CCTTCC
ATTTTT
TTTTTA
CTCCTC
ATTTAT
TTTTAT
CTCCAC
CTGCCT
CATACA
TCCAAC
ATAAAT
TTGTAT
ATTAAA
GTTTTA
TGGTGG
ATGTTT
TAAATT
TAAATA
ATATGT
ATAAAC
ATATAT
TACATA
CTGATA
TGTGTA
ATTGTT
TTTTAA
GTTTTT
TCTCCA
ACGAAA
TTGACA
TATTTA
TGTTTT
GCTGGA
CCACCC
TTAGCG
ATACAT
GTGTAA
CTCCCA
TTTGTA
CACCCC
CTTCCC
ACAAAC
TATATA
TCCACC
CCCAGT
CTGCCC
TGCCTC
AGTGAC
TTTCAG
GGTAAT
ACTGCC
CCTGTG
AAACCC
TCATGA
TTTATT
CAATAA
TTTTTT
TGAGGA
TTATTT
CAAACC
TGAATT
TTTTGT
TAAACA
TAAGAT
CCCCCT
CCCTCC
CCTCCC
CAAAAC
TCTCTC
CCCACT
TAAAAT
AATGTT
GTGTAT
TATATT
TAATAT
CCTCTC
GCACCC
GGCTAC
TCCCAG
CAGGGT
ATTTAA
TTCTTT
CATAAA
AGTCTA
CTTACA
AAAAAC
GGGCTT

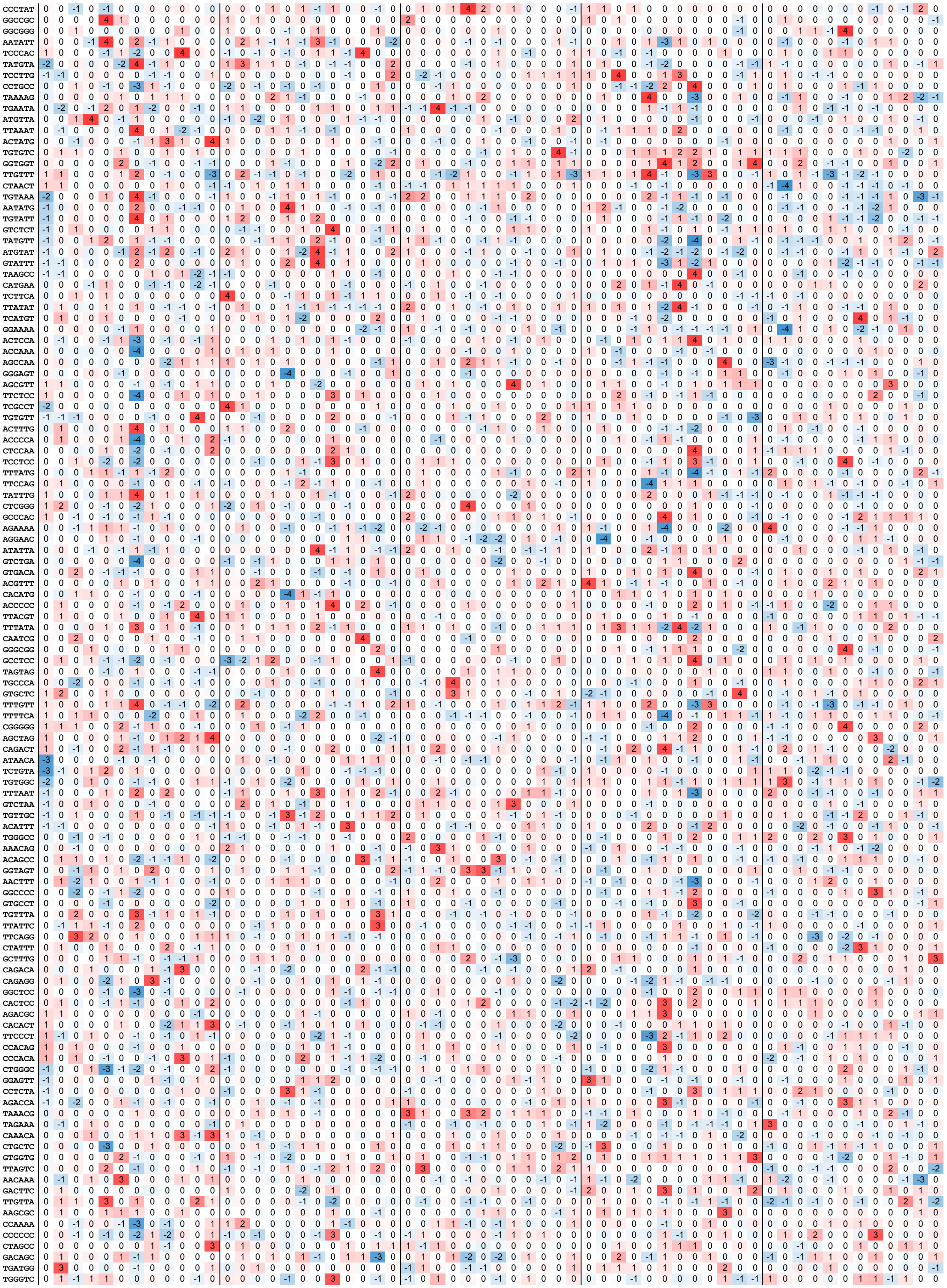
CCCTAT
GGCCGC
GGCGGG
AATATT
TCCCAC
TATGTA
TCCTTG
CCTGCC
TAAAAG
TGAATA
ATGTTA
TTAAAT
ACTATG
TGTGTC
GGTGGT
TTGTTT
CTAACT
TGTAAA
AATATG
TGTATT
GTCTCT
TATGTT
ATGTAT
GTATTT
TAAGCC
CATGAA
TCTTCA
TTATAT
TCATGT
GGAAAA
ACTCCA
ACCAAA
AGCCAA
GGGAGT
AGCGTT
TTCTCC
TCGCCT
TGTGTT
ACTTTG
ACCCCA
CTCCAA
TCCTCC
TTTATG
TTCCAG
TATTTG
CTCGGG
GCCCAC
AGAAAA
AGGAAC
ATATTA
GTCTGA
GTGACA
ACGTTT
CACATG
ACCCCC
TTACGT
TTTATA
CAATCG
GGGCGG
GCCTCC
TAGTAG
TGCCCA
GTGCTC
TTTGTT
TTTTCA
CGGGGG
AGCTAG
CAGACT
ATAACA
TCTGTA
TGTGGC
TTTAAT
GTCTAA
TGTTGC
ACATTT
TGGGCC
AAACAG
ACAGCC
GGTAGT
AACTTT
GGCCCC
GTGCCT
TGTTTA
TTATTC
TTCAGG
CTATTT
GCTTTG
CAGACA
CAGAGG
GGCTCC
CACTCC
AGACGC
CACACT
TTCCCT
CCACAG
CCCACA
CTGGGC
GGAGTT
CCTCTA
AGACCA
TAAACG
TAGAAA
CAAACA
CTGCTC
GTGGTG
TTAGTC
AACAAA
GACTTC
TTGTTA
AAGCGC
CCAAAA
CCCCCC
CTAGCC
GACAGC
TGATGG
TGGGTC

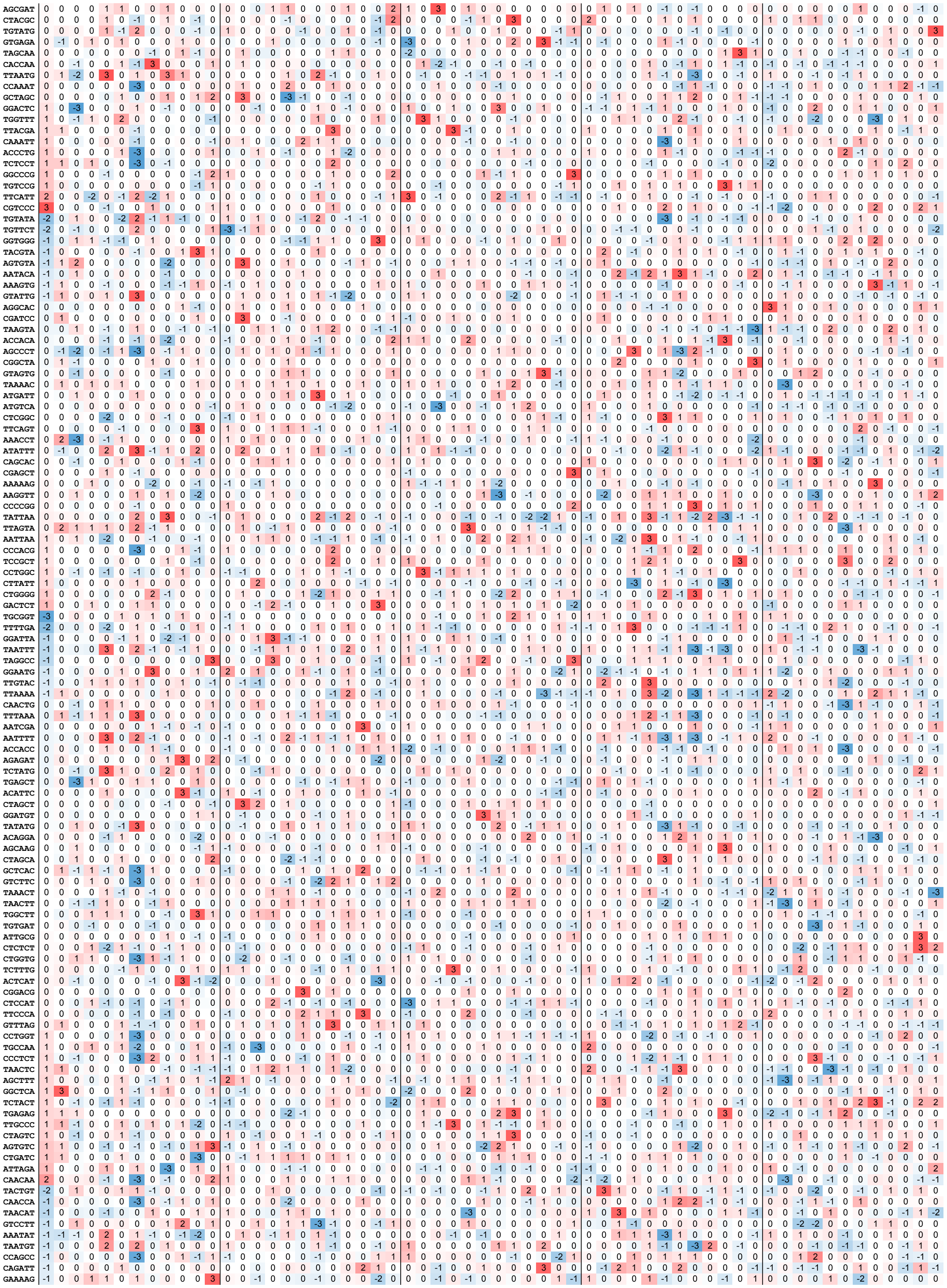
AGCGAT
CTACGC
TGTATG
GTGAGA
TAGCAA
CACCAA
TTAATG
CCAAAT
GCTAGC
GGACTC
TGGTTT
TTACGA
CAAATT
ACCCTG
TCTCCT
GGCCCG
TGTCCG
TTCATT
CGTCCC
TGTATA
TGTTCT
GGTGGG
TACGTA
AGTGTA
AATACA
AAAGTG
GTATTG
AGGCAC
CGATCC
TAAGTA
ACCACA
AGCCCT
CGGCTA
GTAGTG
TAAAAC
ATGATT
ATGTCA
CTCGGC
TTCAGT
AAACCT
ATATTT
CAGCAC
CGAGCT
AAAAAG
AAGGTT
CCCCGG
TATTAA
TTAGTA
AATTAA
CCCACG
TCCGCT
CCTGGC
CTTATT
CTGGGG
GACTCT
TGCGGT
TTTTGA
GGATTA
TAATTT
TAGGCC
GGAATG
TTGTAC
TTAAAA
CAACTG
TTTAAA
AATCGA
AATTTT
ACCACC
AGAGAT
TCTATG
TGAGCT
ACATTC
CTAGCT
GGATGT
TATATG
ACAGGA
AGCAAG
CTAGCA
GCTCAC
GTCTTC
TAAACT
TAACTT
TGGCTT
TGTGAT
ATTGCG
CTCTCT
CTGGTG
TCTTTG
ACTCAT
CGGACG
CTCCAT
TTCCCA
GTTTAG
CCTGGT
TGCCAA
CCCTCT
TAACTC
AGCTTT
GGCTCA
TCTACT
TGAGAG
TTGCCC
CTAGTC
AGTGTC
CTGATC
ATTAGA
CAACAA
TACTGT
CAACCA
TAACAT
GTCCTT
AAATAT
TAATGT
CCAGCC
CAGATT
GAAAAG

TGTTAA	-1	0	0	1	1	0	2	0	1	0	0	0	0	0	-3	0	1	-1	0	0	1	0	-1	-1	0	0	1	1	0	0	0	0	1	0	-1	0	0	1	0	0	0	-1	1	-2	0	-1	2	0	0	0	0	0	-1	0	1	0	0	0	0	-1
TCTCCC	0	-1	0	0	0	0	-1	1	-1	1	3	1	0	-1	0	0	0	0	0	1	0	2	0	0	1	0	-1	1	0	1	0	0	0	0	0	0	-1	0	0	0	-2	1	-1	1	0	1	0	-1	1	0	0	1	0	0	0	1	0	0	0	0
TTCTGG	0	0	-1	0	1	1	0	1	-1	0	0	1	0	0	-1	0	0	0	-3	0	-1	-1	0	0	0	0	-1	0	0	0	0	0	-1	0	0	0	1	0	0	0	1	1	0	0	0	0	0	0	1	0	1	0	0	0	1	0	0	0	0	0
GGAGGC	0	0	0	0	0	0	-2	0	0	0	0	0	0	-1	0	0	0	0	0	0	0	0	0	0	1	0	-1	0	1	1	0	1	0	0	0	0	-1	0	0	0	0	1	0	0	0	0	2	1	3	0	-1	0	0	1	0	1	0	0	0	0
GTAAAA	0	0	0	0	1	0	1	0	0	0	0	0	-1	-1	0	1	1	0	0	-1	-1	0	0	0	3	1	0	1	0	1	0	0	-1	2	-1	0	0	1	-1	-2	1	0	-1	-1	0	0	1	-1	0	-3	0	1	-1	-1	-1	0	0	0	0	-1
TGGAGC	0	0	0	1	-1	2	0	0	-1	1	0	0	0	0	0	0	0	3	-1	0	0	0	0	0	0	1	0	0	0	0	0	0	-1	1	-1	0	0	0	1	0	0	1	0	0	0	0	0	1	0	0	1	-1	0	0	-1	0	0	1	0	0
CACTGT	0	1	0	0	0	1	0	0	0	1	1	0	0	1	0	0	1	0	-1	0	0	1	1	1	-3	0	-2	0	0	-1	0	-1	0	1	0	0	0	1	0	0	0	0	0	0	0	0	1	0	0	1	0	1	0	0	0	0	0	0	0	0
CTGTGC	0	0	0	2	0	-1	0	1	0	0	1	1	-1	0	0	1	-1	0	0	0	0	0	-1	1	1	0	0	0	0	1	0	0	0	-1	-1	0	-3	0	-1	2	0	1	0	1	0	0	0	0	0	1	0	0	0	2	0	0	0	0	0	0
GAGGAC	0	0	0	0	0	0	0	1	0	1	-1	2	2	1	0	0	-3	-1	-1	0	0	1	0	0	0	0	0	0	0	0	-1	0	0	0	0	0	1	0	1	0	0	1	1	0	0	1	0	0	-1	0	0	0	0	0	-1	1	0	0	0	0
CCCCTC	0	0	0	0	-1	0	-3	0	0	0	0	1	0	-1	0	0	0	0	1	0	1	1	0	0	0	0	0	0	2	1	1	0	-1	0	1	0	0	0	-1	1	-1	1	-1	3	0	0	1	0	0	0	0	3	0	0	-1	1	0	-1	0	0
GATGGA	0	1	0	0	-2	0	1	0	-1	0	0	0	0	0	1	1	0	1	-1	0	0	0	0	2	0	-2	0	1	-3	0	0	0	-1	0	0	0	0	0	0	2	0	1	0	0	0	1	-1	-1	0	1	1	1	0	1	-1	0	0	0	0	0
TCAAAT	0	0	0	0	0	0	-1	0	0	0	0	-1	0	-2	0	0	0	0	0	1	0	0	1	-1	0	0	-1	-2	0	1	0	-1	0	0	0	1	0	0	0	1	-1	-2	0	0	0	1	0	0	2	0	0	1	1	0	3	1	-1	1	1	0
TCTAGA	0	0	-1	0	1	0	1	-1	0	0	0	0	0	0	0	0	-1	0	0	0	0	0	1	0	0	0	1	0	0	0	1	0	1	1	0	3	0	0	0	1	0	-1	0	1	1	0	0	0	1	0	0	0	-1	0	0	0	-1	0	0	0
AGTGAA	0	2	0	0	0	1	1	0	0	1	-1	0	1	0	0	1	0	0	0	0	1	0	0	0	0	0	1	1	0	0	0	0	1	1	1	0	0	-1	1	-1	1	0	0	-1	0	0	0	0	0	0	1	-1	-1	0	0	3	-1	1	-1	0
ATCCGT	0	0	0	0	0	0	0	3	0	0	-1	0	0	0	0	0	0	0	-1	1	-1	0	0	0	1	0	1	0	0	0	0	2	0	0	1	0	0	0	0	0	0	-1	0	1	0	0	1	0	0	1	0	0	0	-1	1	0	0	0	0	0
CAGGCA	0	0	0	0	-1	1	-1	1	0	1	-3	0	0	1	0	0	0	2	0	0	-1	0	0	0	0	0	0	0	0	0	0	0	1	0	-1	1	1	0	1	1	-1	1	0	1	0	1	0	0	0	1	0	1	0	0	1	0	0	0	0	1
CCTCCT	0	0	0	1	-1	-1	-2	0	0	1	0	0	-1	0	0	0	-1	2	0	3	0	0	0	1	0	1	1	0	0	1	0	0	1	0	0	0	-2	0	0	0	0	3	0	2	-1	1	0	1	0	0	0	0	0	2	-2	-2	0	0	0	0
TCCCTC	0	0	1	0	-1	0	-2	0	-2	0	0	0	0	0	0	0	0	0	-2	1	-1	0	0	1	0	1	0	0	0	0	0	1	0	1	0	0	0	0	0	0	-3	1	-1	0	0	1	0	0	0	0	0	0	0	2	1	0	0	0	0	1
TTAAAC	0	0	0	1	0	0	0	0	1	0	0	1	0	0	0	1	1	0	1	0	0	0	0	-2	0	0	1	1	0	0	1	1	0	0	1	0	-1	0	0	1	3	0	0	-1	2	-1	0	0	0	1	0	0	0	-2	0	-1	0	-2	0	-1
CCCCAC	0	0	2	0	0	1	-3	-1	0	3	0	2	-1	0	0	0	0	1	-2	2	-1	0	0	0	0	0	1	0	0	1	-1	0	0	1	-1	0	0	-1	1	0	0	1	-1	3	0	0	0	0	0	1	-1	0	0	1	-1	2	0	0	1	2
TACTAT	0	0	0	-1	-1	0	0	0	1	0	0	0	1	0	0	0	1	1	0	0	0	0	0	-1	0	0	0	0	0	0	0	0	0	-1	0	-1	0	0	0	0	1	-1	0	0	0	0	0	0	0	-1	0	0	3	1	0	1	1	0	1	0
TCCTGG	0	1	0	0	-1	0	-3	0	0	0	0	0	-1	0	0	0	-1	0	0	1	0	0	-1	-1	0	1	1	0	0	0	0	0	0	0	0	0	-1	-1	-1	0	-1	1	-1	1	0	0	0	0	0	-1	1	1	0	0	0	0	0	0	0	0
ACCATT	0	0	1	0	0	1	0	0	0	0	0	0	0	1	1	0	0	-1	1	0	0	-1	1	0	1	0	0	0	-1	0	0	0	0	0	0	0	0	0	1	0	0	1	0	0	0	0	1	0	0	-2	0	0	1	0	3	1	1	-1	0	0
CACAAC	0	1	0	0	-1	0	-2	0	0	1	-1	1	0	0	-1	0	-3	0	0	0	0	1	1	0	0	0	-1	0	0	1	-1	0	0	0	0	0	-1	0	0	0	0	2	0	0	0	0	-1	-1	0	0	-1	1	0	0	2	0	0	0	0	0
CTCCTG	0	1	0	0	-3	0	-1	-1	-1	0	-1	0	0	0	0	2	0	0	0	1	0	0	0	0	0	0	0	1	0	1	0	0	1	0	0	0	-1	0	0	0	-1	1	0	2	0	1	1	1	-1	0	0	0	1	2	-1	1	0	0	1	0
AATGGC	0	0	0	0	0	-1	1	0	-1	0	0	0	0	1	-1	0	1	0	1	0	1	3	1	0	0	0	0	0	0	0	1	0	0	0	-1	0	0	0	0	0	0	0	0	0	0	0	-1	0	0	0	0	0	-1	1	0	-1	0	1	1	0
AGCATG	0	0	0	1	1	2	-1	0	0	0	0	0	0	0	0	2	1	0	0	1	0	0	1	0	-3	0	-1	0	0	0	1	0	0	0	0	0	0	1	-1	0	0	1	-1	1	0	0	0	0	0	-1	0	0	-1	0	0	0	0	0	0	-1
CGCCAA	0	0	0	0	-3	0	-1	0	-1	0	0	2	0	0	0	0	0	0	0	1	0	0	-1	0	0	0	0	0	0	0	0	1	0	0	0	0	0	1	0	0	0	0	0	1	0	0	0	0	0	0	0	0	0	0	0	0	0	1	0	0
TGCAAG	0	0	1	0	1	0	0	0	0	1	0	1	1	0	0	0	0	0	0	0	0	0	0	0	-1	1	1	1	-1	1	0	1	0	0	-1	0	0	0	0	0	1	0	0	0	0	-1	-1	0	-1	-3	-1	-3	0	0	0	0	0	0	1	0
TGTCGG	0	1	0	0	0	1	0	0	0	0	1	0	0	1	0	0	0	1	0	0	0	0	0	0	0	1	-1	0	0	0	0	0	0	0	0	0	0	0	0	0	3	2	0	0	1	0	1	0	0	0	0	0	-1	0	0	0	0	0	0	0
GGGAGC	1	1	0	0	-2	1	-1	1	0	0	0	0	0	0	0	0	0	1	0	0	0	0	0	0	0	1	0	0	-1	0	0	1	2	1	0	0	0	0	0	0	-1	0	0	0	0	0	1	1	-1	1	1	0	1	3	1	0	0	-1	0	2
GTGTTT	1	0	0	1	0	0	1	0	0	0	3	1	0	-1	0	-1	0	1	0	1	0	0	0	1	1	1	0	1	0	0	1	0	0	1	0	0	0	0	0	1	0	0	0	-1	0	-1	-1	-3	0	0	1	0	0	-1	1	1	0	1	1	0
GTTTTC	1	0	-1	1	1	0	1	-2	0	0	1	-1	0	1	0	1	0	0	0	0	0	0	0	0	-1	0	-2	0	0	-3	2	-1	0	0	0	-1	0	1	1	0	1	-1	0	-1	1	0	-2	-1	0	0	1	1	0	0	0	0	-1	0	0	0
TGCAAC	1	0	0	0	0	0	0	0	3	0	0	0	1	0	0	0	0	0	-1	1	0	0	0	0	1	0	0	0	1	1	0	1	0	2	-1	0	0	-1	0	0	0	0	0	0	1	-2	0	1	0	0	0	0	0	0	0	0	0	0	1	0
GAGAGA	1	0	0	0	0	0	1	0	1	1	0	0	1	0	0	0	-3	-1	0	1	0	0	0	0	0	0	0	1	1	0	1	1	1	0	0	0	0	0	0	0	-2	1	0	0	0	0	-1	-1	0	0	0	0	1	1	0	0	0	0	1	-1
ATGGCA	1	1	0	0	-1	0	0	0	-2	0	0	1	1	0	0	0	0	-1	1	0	1	3	0	0	1	0	-1	0	0	0	2	0	0	1	-1	0	0	0	0	0	0	0	0	0	1	0	0	1	0	0	0	1	0	2	0	-1	-2	0	-1	0
GTCCAC	1	0	-1	0	0	0	0	0	-1	0	0	0	0	1	0	0	0	1	1	1	0	1	0	0	-1	-1	0	1	0	0	2	0	1	0	0	0	-2	0	-1	0	0	0	0	0	0	3	-1	0	0	1	-1	0	0	0	-1	-1	-1	0	0	0
TCACAG	3	1	0	0	-2	0	-1	1	0	3	-2	0	-2	0	0	-1	0	0	0	1	0	0	0	0	-1	-1	0	-1	0	1	0	-1	0	0	1	0	0	-1	0	0	1	1	0	0	0	0	0	1	1	0	0	0	-1	0	0	0	0	0	0	0
