# Supplementary material for: Systematic Profiling of Poly(A)+ Transcripts Modulated by Core 3’ End Processing and Splicing Factors Reveals Regulatory Rules of Alternative Cleavage and Polyadenylation
Source: PLoS Genet. 2015 Apr 23;11(4):e1005166. doi: 10.1371/journal.pgen.1005166 (PMC4407891; doi:10.1371/journal.pgen.1005166)
Supplement: S7 Table — (PDF) [file pgen.1005166.s020.pdf]

Gene Group	Gene Symbol
1	2400001E08Rik
1	2410091C18Rik
1	2610101N10Rik
1	2700023E23Rik
1	5730403B10Rik
1	6720456H20Rik
1	Abhd13
1	Abhd2
1	Actn1
1	Adnp2
1	Adsl
1	Aif1l
1	Ak3
1	Akap8
1	Akt1s1
1	Ankib1
1	Ankrd33b
1	Ap1ar
1	Ap1b1
1	Apex2
1	Arf6
1	Arhgap24
1	Arhgap29
1	Arhgef19
1	Arl2bp
1	Atg5
1	Atp5a1
1	Azin1
1	Bag4
1	Bard1
1	Bdnf
1	Bmpr1a
1	Bnip2
1	Brf1
1	Brix1
1	C1galt1c1
1	Canx
1	Ccnh
1	Cdk19
1	Cdk5
1	Cfl2
1	Chst2
1	Cox15
1	Cpsf7
1	Crk
1	Cyp1b1
1	Cyp51

1	Dcaf10
1	Dctn2
1	Ddx3x
1	Derl1
1	Dmrta2
1	Dnajc10
1	Dock5
1	Dr1
1	Egln3
1	Eid1
1	Enah
1	Etaa1
1	Fam110b
1	Fam3c
1	Fam59a
1	Fbrs
1	Fbxo28
1	Fbxo3
1	Fech
1	Fem1a
1	Fgfr1
1	Flrt2
1	Fnta
1	Frrs1
1	Gapvd1
1	Gdap2
1	Ghitm
1	Gm10052
1	Gm17296
1	Gm5643
1	Gnb1
1	Gpr137b
1	Gtf2e1
1	Gxylt2
1	Hif1a
1	Hipk3
1	Hnrnpab
1	Homer1
1	Hoxa10
1	Hoxc5
1	Ifit2
1	Ikbkb
1	Imp4
1	Incenp
1	Isoc1
1	Kdelr2
1	Kif14
1	Ldlr

1	Lpcat3
1	Lrp5
1	Lrrc8b
1	Mapk8
1	Mapk9
1	Matr3
1	Mdm1
1	Mecp2
1	Med28
1	Mfap3l
1	Mical2
1	Mmd
1	Mon2
1	Morc2a
1	Mrs2
1	Mta1
1	Mtpn
1	Myadm
1	Naa30
1	Naa50
1	Nck1
1	Ndufb5
1	Nfyb
1	Nktr
1	Nop10
1	Notch2
1	Nprl3
1	Nras
1	Pde7a
1	Pdp1
1	Phf12
1	Pik3ca
1	Pik3cb
1	Pkp4
1	Plcl1
1	Ppm1a
1	Prdx1
1	Prepl
1	Psmc6
1	Psmd8
1	Rab33b
1	Rab8a
1	Ranbp2
1	Rap2b
1	Rbm39
1	Rbpj
1	Rcan2
1	Rer1

1	Rnf217
1	Rreb1
1	Rrm2
1	Rtn3
1	Rwdd3
1	Sco1
1	Sdcbp
1	Sel1l
1	Senp2
1	Senp5
1	Serf2
1	Serinc3
1	Sgk3
1	Sh3glb1
1	Shmt1
1	Shmt2
1	Slc25a17
1	Slc25a25
1	Slc30a7
1	Slc38a1
1	Slc7a5
1	Smndc1
1	Snrpd1
1	Snx21
1	Snx7
1	Sox9
1	Spcs3
1	Ssb
1	Ssr1
1	Star
1	Steap3
1	Strap
1	Swap70
1	Syde2
1	Synj2
1	Syt13
1	Tfam
1	Tfdp1
1	Tiparp
1	Tmem167
1	Tmem30a
1	Tmem64
1	Tprgl
1	Tra2b
1	Traf6
1	Trappc10
1	Trim23
1	Trub2

1	Ttc9
1	Tusc3
1	Txlna
1	Uba1
1	Ube2b
1	Ube2e2
1	Ube2g1
1	Ubqln1
1	Uhrf1bp1l
1	Vgll3
1	Vps36
1	Ypel5
1	Ywhaz
1	Zbtb41
1	Zc3h14
1	Zeb2
1	Zfp189
1	Zfp260
1	Zfp316
1	Zfp655
1	Zmym2
2	0610009B22Rik
2	0610030E20Rik
2	1110002B05Rik
2	1110004F10Rik
2	1110031I02Rik
2	1110032A03Rik
2	1110034B05Rik
2	1500011H22Rik
2	1700020I14Rik
2	1700020O03Rik
2	1700025G04Rik
2	1700066M21Rik
2	1810009A15Rik
2	1810030O07Rik
2	1810043G02Rik
2	2310028H24Rik
2	2310035C23Rik
2	2310045N01Rik
2	2310057M21Rik
2	2410004B18Rik
2	2410042D21Rik
2	2410075B13Rik
2	2410127L17Rik
2	2610002I17Rik
2	2610002M06Rik
2	2610507B11Rik
2	2700078E11Rik

2	2810008M24Rik
2	2810407C02Rik
2	2810408M09Rik
2	2900026A02Rik
2	2900053A13Rik
2	2900064A13Rik
2	3110043O21Rik
2	3830406C13Rik
2	4921531C22Rik
2	4922501C03Rik
2	4930402H24Rik
2	4930422G04Rik
2	4930430F08Rik
2	4930453N24Rik
2	4930471M23Rik
2	4930506M07Rik
2	4930579G24Rik
2	4931406P16Rik
2	4931414P19Rik
2	4931428F04Rik
2	4933403F05Rik
2	5930434B04Rik
2	6030446N20Rik
2	6030458C11Rik
2	6430548M08Rik
2	6720463M24Rik
2	8430410K20Rik
2	9230114K14Rik
2	9430016H08Rik
2	9430020K01Rik
2	9830001H06Rik
2	A130022J15Rik
2	A530084C06Rik
2	A730008H23Rik
2	AC114005.5
2	AI450353
2	AI597468
2	AI646023
2	AI848100
2	AW146020
2	Aasdhppt
2	Abca5
2	Abcb7
2	Abcc4
2	Abce1
2	Abhd10
2	Abhd5
2	Ablim1

2	Abr
2	Acad8
2	Acap2
2	Acbd4
2	Acer3
2	Acp1
2	Acp2
2	Acsl1
2	Acsl4
2	Actr3
2	Acvr2a
2	Adam12
2	Adam17
2	Adamts1
2	Adamts2
2	Adamts5
2	Adamts6
2	Adarb1
2	Add3
2	Adipor1
2	Ado
2	Adrbk1
2	Adss
2	Aen
2	Aff1
2	Afg3l1
2	Agtpbp1
2	Ahctf1
2	Akap10
2	Akap11
2	Akap7
2	Akirin2
2	Akr1e1
2	Aktip
2	Alcam
2	Aldh5a1
2	Alg10b
2	Alg14
2	Als2
2	Ampd3
2	Anapc10
2	Ank
2	Ankfy1
2	Ankrd10
2	Ankrd13c
2	Ankrd28
2	Ankrd40
2	Ankrd50

2	Ankrd57
2	Anln
2	Anxa7
2	Ap1g1
2	Ap2a2
2	Ap3m1
2	Ap4e1
2	Apbb2
2	Aph1b
2	App
2	Appl1
2	Arcn1
2	Arfip1
2	Arfip2
2	Arhgap11a
2	Arhgap32
2	Arhgap5
2	Arhgap8
2	Arhgef10l
2	Arhgef17
2	Arhgef2
2	Arid2
2	Arid4b
2	Arid5b
2	Arl13b
2	Arl15
2	Arl4a
2	Arl4c
2	Arl5b
2	Arl6ip1
2	Arl6ip6
2	Arl8b
2	Armc8
2	Arnt
2	Arpp19
2	Arsa
2	Ash1l
2	Asph
2	Aste1
2	Asxl1
2	Atad2
2	Atad2b
2	Atad5
2	Atf2
2	Atg12
2	Atl3
2	Atm
2	Atn1

2	Atp11c
2	Atp13a3
2	Atp2a2
2	Atp2b1
2	Atp2c1
2	Atp6ap2
2	Atp6v1a
2	Atp6v1b2
2	Atp6v1c1
2	Atp6v1g1
2	Atp6v1g2
2	Atp8b2
2	Atpbd4
2	Atrx
2	Atxn1l
2	Avl9
2	B230219D22Rik
2	B3galtl
2	B4galt1
2	B630005N14Rik
2	BC003965
2	BC005537
2	BC024659
2	BC037034
2	BC055324
2	Bace1
2	Baz2a
2	Baz2b
2	Bcas2
2	Bccip
2	Bclaf1
2	Bcorl1
2	Bcr
2	Bend3
2	Bhlhe40
2	Bhlhe41
2	Bicd2
2	Blmh
2	Blzf1
2	Bmpr2
2	Bnip3l
2	Bola2
2	Bpnt1
2	Braf
2	Brap
2	Brd3
2	Brd8
2	Bri3bp

2	Brwd3
2	Btbd1
2	Btbd10
2	Btbd11
2	Btg2
2	C030006K11Rik
2	C030046E11Rik
2	C1d
2	C1galt1
2	C230081A13Rik
2	C230096C10Rik
2	C330027C09Rik
2	C3ar1
2	C78339
2	Cab39
2	Cables2
2	Cachd1
2	Cadm1
2	Cand1
2	Caprin1
2	Capza2
2	Capzb
2	Car8
2	Card10
2	Casc3
2	Cask
2	Cbfb
2	Cblb
2	Cbll1
2	Ccbe1
2	Ccdc137
2	Ccdc15
2	Ccdc25
2	Ccdc55
2	Ccdc59
2	Ccdc86
2	Ccdc90a
2	Ccm2
2	Ccnb1
2	Ccng1
2	Ccni
2	Ccnl2
2	Ccny
2	Ccrn4l
2	Cct4
2	Cd164
2	Cd34
2	Cd44

2	Cdc14b
2	Cdc27
2	Cdc37
2	Cdc37l1
2	Cdc42bpa
2	Cdca3
2	Cdca7l
2	Cdipt
2	Cdk17
2	Cdk7
2	Cdkn2aip
2	Cdkn3
2	Cdon
2	Cdr2l
2	Cebpb
2	Cebpz
2	Celf2
2	Cenpf
2	Cenph
2	Cenpq
2	Cep120
2	Cep57
2	Cep68
2	Cerk
2	Chchd4
2	Chd1
2	Chd2
2	Chd7
2	Chd9
2	Chek1
2	Chm
2	Chmp2b
2	Chmp3
2	Chmp4b
2	Chmp5
2	Chmp7
2	Chn1
2	Chn2
2	Chordc1
2	Chst3
2	Ciao1
2	Cited2
2	Cldn25
2	Cldnd1
2	Clic4
2	Clint1
2	Clip1
2	Clip3

2	Cln8
2	Clspn
2	Clta
2	Cltb
2	Cmc1
2	Cnnm2
2	Cno
2	Cnot2
2	Cnot4
2	Cnot7
2	Cnpy3
2	Cntf
2	Cobll1
2	Cog3
2	Col12a1
2	Col16a1
2	Col1a2
2	Col5a2
2	Col8a1
2	Commd7
2	Commd8
2	Copa
2	Cops2
2	Cops5
2	Copz1
2	Coq10b
2	Coq3
2	Cpd
2	Cpne2
2	Cpne3
2	Cpsf2
2	Cpsf6
2	Cradd
2	Cramp1l
2	Creb3
2	Crebbp
2	Crim1
2	Crkl
2	Crtc1
2	Csgalnact1
2	Csgalnact2
2	Csnk1a1
2	Csnk1g1
2	Csrnp1
2	Cstf2
2	Ctbp2
2	Ctdsp2
2	Ctnna1

2	Ctnnd1
2	Ctps
2	Cttn
2	Cul4a
2	Cul4b
2	Cyb5d2
2	Cyb5r4
2	Cyfip1
2	Cyld
2	Cyp2j6
2	Cyth3
2	D030016E14Rik
2	D17Wsu92e
2	D730040F13Rik
2	D930016D06Rik
2	Daam1
2	Dbnl
2	Dcaf12
2	Dcaf7
2	Dcaf8
2	Dcbld1
2	Dcbld2
2	Dck
2	Dctd
2	Dcun1d4
2	Ddhd1
2	Ddi2
2	Ddit4
2	Ddr2
2	Ddx51
2	Ddx58
2	Dedd2
2	Dennd1b
2	Dennd5b
2	Dera
2	Dgcr8
2	Dgkh
2	Dgkq
2	Dhx40
2	Diablo
2	Dido1
2	Dirc2
2	Dkc1
2	Dlat
2	Dlc1
2	Dlg3
2	Dna2
2	Dnaja2

2	Dnaja3
2	Dnajb1
2	Dnajc16
2	Dnajc5
2	Dnlz
2	Dnm1l
2	Dock4
2	Dph1
2	Dram2
2	Dscr3
2	Dst
2	Dusp16
2	Dusp18
2	Dusp6
2	Dync1li2
2	Dynll1
2	Dyrk1a
2	Dyrk2
2	Dzip1
2	Dzip3
2	E130308A19Rik
2	E2f3
2	
2	Ebag9
2	Ecd
2	Echdc1
2	Echs1
2	Edc3
2	Edem1
2	Edem3
2	Eed
2	Efha1
2	Efna5
2	Efnb2
2	Ehd4
2	Eif1a
2	Eif2ak3
2	Eif3a
2	Eif5
2	Elac1
2	Elf2
2	Elf4
2	Elmod2
2	Elovl7
2	Emp2
2	Enc1
2	Enoph1
2	Enox2

2	Enpp4
2	Epb4.1l3
2	Epha4
2	Ephb2
2	Ephx1
2	Epm2aip1
2	Eps15
2	Ept1
2	Erap1
2	Erbb2ip
2	Ercc4
2	Ercc6
2	Ergic1
2	Ergic2
2	Erich1
2	Ermp1
2	Esco2
2	Etf1
2	Etl4
2	Etnk1
2	Ets2
2	Exoc5
2	Exoc8
2	Exosc9
2	Eya1
2	Fads3
2	Faim
2	Fam101b
2	Fam107b
2	Fam110c
2	Fam114a2
2	Fam115a
2	Fam117b
2	Fam118a
2	Fam120a
2	Fam120b
2	Fam126b
2	Fam131b
2	Fam133b
2	Fam135a
2	Fam149a
2	Fam160b1
2	Fam164a
2	Fam171a1
2	Fam175b
2	Fam179b
2	Fam18b
2	Fam20b

2	Fam33a
2	Fam53b
2	Fam76b
2	Fam83d
2	Fanca
2	Fancd2
2	Fancm
2	Far1
2	Fasn
2	Fat1
2	Fbn1
2	Fbxl3
2	Fbxo21
2	Fbxo25
2	Fbxo30
2	Fbxo42
2	Fbxo45
2	Fem1c
2	Fer
2	Fermt2
2	Fert2
2	Fgd3
2	Fgfr1op
2	Fitm2
2	Fkbp1a
2	Fkbp5
2	Fkbp9
2	Fmn1
2	Fmnl2
2	Fmr1
2	Fnbp1
2	Fnbp4
2	Fndc3b
2	Fntb
2	Fosl2
2	Foxn2
2	Foxo1
2	Fpgt
2	Frmd4a
2	Fsd1l
2	Fubp3
2	Fundc1
2	Fyb
2	Fyco1
2	Fyttd1
2	Fzd1
2	Fzd2
2	Fzd6

2	G3bp1
2	Gabpb1
2	Galnt2
2	Galnt7
2	Gas2l3
2	Gata2
2	Gatad1
2	Gatad2b
2	Gbas
2	Gcc2
2	Gcfc1
2	Gclc
2	Gdpd1
2	Gen1
2	Gfm1
2	Ggnbp2
2	Ghr
2	Gipc1
2	Git2
2	Gjc1
2	Glrb
2	Glrp1
2	Glrx2
2	Glrx5
2	Gls
2	Gm15450
2	Gm15772
2	Gm2058
2	Gm9790
2	Gmcl1
2	Gmeb1
2	Gmps
2	Gna11
2	Gnb4
2	Gng11
2	Gnl1
2	Gnl3l
2	Golga2
2	Golga5
2	Golim4
2	Golt1b
2	Gorasp2
2	Gosr1
2	Gosr2
2	Got1
2	Gpatch2
2	Gpbp1
2	Gpcpd1

2	Gpd1l
2	Gpd2
2	Gpn1
2	Gpr126
2	Gpt2
2	Grem1
2	Grk6
2	Grlf1
2	Grpel1
2	Gtf2h5
2	Gtf3c2
2	H3f3b
2	Hcfc1
2	Hdac6
2	Heatr5a
2	Heatr6
2	Heca
2	Hectd1
2	Herc4
2	Hexim1
2	Hgf
2	Hhip
2	Hiat1
2	Hinfp
2	Hipk2
2	Hist3h2a
2	Hivep1
2	Hjurp
2	Hk2
2	Hmgcr
2	Hmgn1
2	Hmgxb4
2	Hnrnpk
2	Hnrnpul2
2	Hnrpdl
2	Hook3
2	Hoxa13
2	Hps3
2	Hs1bp3
2	Hsd17b12
2	Hspa13
2	Hspa1b
2	Hspa4l
2	Hspa5
2	Htr1b
2	Hus1
2	Iah1
2	Ick

2	Ids
2	Ifnar1
2	Ift122
2	Ift88
2	Igf2bp2
2	Ikbkap
2	Ikbkg
2	Il13ra1
2	Il1rap
2	Il34
2	Ilf3
2	Impa2
2	Impad1
2	Inadl
2	Ing2
2	Ing4
2	Insig2
2	Ints2
2	Ints7
2	Ipo5
2	Ipo7
2	Ipo9
2	Iqce
2	Iqsec2
2	Irf2bp1
2	Irf9
2	Isca1
2	Itpr1
2	Ivns1abp
2	Iws1
2	Jag1
2	Jak1
2	Jhdm1d
2	Jmjd1c
2	Jmy
2	Jun
2	Jup
2	Katnal1
2	Kbtbd2
2	Kcmf1
2	Kcnj15
2	Kctd11
2	Kctd5
2	Kctd6
2	Kdm1b
2	Kdm3b
2	Kdm5b
2	Kdm5c

2	Kdm6a
2	Khdrbs3
2	Kif13a
2	Kif16b
2	Kif18b
2	Kif21a
2	Kif21b
2	Kif26b
2	Kif3a
2	Kirrel
2	Klf11
2	Klf3
2	Klf5
2	Klf6
2	Klhdc10
2	Klhl24
2	Klhl9
2	Kpna1
2	Kpna3
2	Kpna4
2	Krit1
2	Ktn1
2	Lancl3
2	Laptm4b
2	Lass5
2	Lats1
2	Lats2
2	Lclat1
2	Lcor
2	Lemd2
2	Lemd3
2	Lepr
2	Lepre1
2	Leprot
2	Leprotl1
2	Lfng
2	Lgr6
2	Lhfpl2
2	Lias
2	Lig3
2	Limd1
2	Limd2
2	Lims1
2	Litaf
2	Lix1l
2	Lman2l
2	Lmbrd1
2	Lmbrd2

2	Lmf2
2	Lnp
2	Lonrf3
2	Loxl4
2	Lpgat1
2	Lphn2
2	Lpin2
2	Lpp
2	Lrp12
2	Lrp6
2	Lrr1
2	Lrrc1
2	Lrrc14
2	Lrrc47
2	Lrrc58
2	Lrrc8d
2	Lrrfip1
2	Lsm10
2	Lsm11
2	Ltbp1
2	Ltn1
2	Luc7l
2	Luc7l3
2	Luzp1
2	Lypla1
2	Lztfl1
2	Maged1
2	Magi2
2	Magt1
2	Maml2
2	Man1a
2	Man1a2
2	Man1c1
2	Man2a1
2	Map1lc3b
2	Map2k2
2	Map4k5
2	Mapk6
2	Mapre3
2	March5
2	March8
2	Marcks
2	Mark3
2	Mars
2	Mars2
2	Mbd2
2	Mbnl1
2	Mcm2

2	Mcm3
2	Mdfic
2	Mdh1
2	Med13
2	Med13l
2	Med23
2	Meis1
2	Memo1
2	Met
2	Metap1
2	Mettl13
2	Mettl21d
2	Mettl4
2	Mettl6
2	Mettl9
2	Mex3a
2	Mex3c
2	Mex3d
2	Mfhas1
2	Mfn1
2	Mgat2
2	Mgea5
2	Mgrn1
2	Mgst3
2	Mid2
2	Mier1
2	Mis12
2	Mkl2
2	Mkrn2
2	Mll5
2	Mmab
2	Mob1b
2	Mob2
2	Mob4
2	Morc3
2	Mpp1
2	Mpp5
2	Mprip
2	Mpzl1
2	Mri1
2	Mrpl12
2	Mrpl16
2	Mrpl17
2	Mrpl3
2	Mrpl42
2	Mrpl47
2	Mrpl50
2	Mrps14

2	Mrrf
2	Msi2
2	Mtap
2	Mtdh
2	Mtpap
2	Mtr
2	Mttp
2	Mtus1
2	Mul1
2	Mus81
2	Mxra8
2	Mycbp
2	Myef2
2	Myf5
2	Mynn
2	Myo1b
2	Myo5a
2	Myo9a
2	Myo9b
2	Myof
2	Mysm1
2	Myst3
2	Mzt1
2	N4bp2
2	N4bp2l2
2	Naa15
2	Naa16
2	Naa40
2	Naalad2
2	Nab1
2	Naf1
2	Nampt
2	Nanos1
2	Narf
2	Nars
2	Ncapd3
2	Ncaph2
2	Ncbp1
2	Nceh1
2	Nckap1
2	Ncoa2
2	Ncoa3
2	Ncoa7
2	Ncor1
2	Ncs1
2	Ndfip2
2	Ndufaf4
2	Ndufc1

2	Necap2
2	Nedd4l
2	Nefl
2	Nek6
2	Neo1
2	Nexn
2	Nf1
2	Nfatc3
2	Nfya
2	Nin
2	Nipbl
2	Nipsnap3a
2	Nipsnap3b
2	Nkain1
2	Nkap
2	Nkiras1
2	Nkrf
2	Nlk
2	Nln
2	Nmd3
2	Nme1
2	Noc3l
2	Nom1
2	Nop16
2	Notch1
2	Nox4
2	Npepps
2	Npm1
2	Nptn
2	Nqo2
2	Nr2c2
2	Nr2f2
2	Nr4a1
2	Nrd1
2	Nsun2
2	Nsun3
2	Nt5c2
2	Nt5c3
2	Nudcd1
2	Nudcd2
2	Nudt12
2	Nudt3
2	Nudt4
2	Nufip2
2	Nup107
2	Nup153
2	Nup155
2	Nup160

2	Nup62
2	Nus1
2	Nxn
2	Nxt1
2	Ogfrl1
2	Ola1
2	Osbpl3
2	Osbpl6
2	Osbpl8
2	Osgep
2	Osgin2
2	Otub1
2	Ovca2
2	Oxa1l
2	Oxnad1
2	Oxr1
2	Pabpc4
2	Paip2
2	Pak2
2	Pak3
2	Pank1
2	Papd7
2	Papola
2	Pappa
2	Paqr8
2	Pard3
2	Parvb
2	Pawr
2	Pbrm1
2	Pccb
2	Pcdh7
2	Pcf11
2	Pcgf2
2	Pcid2
2	Pcmtd1
2	Pcmtd2
2	Pcnx
2	Pcsk7
2	Pcyox1
2	Pdcd6
2	Pdcl
2	Pdcl3
2	Pde12
2	Pde4d
2	Pdf
2	Pdgfd
2	Pdlim5
2	Pdlim7

2	Pds5b
2	Per3
2	Pex11b
2	Pfas
2	Pfdn2
2	Pgam5
2	Pgm1
2	Pgrmc1
2	Phf13
2	Phf2
2	Phf20
2	Phf20l1
2	Phf21a
2	Phldb2
2	Pi4k2b
2	Pias1
2	Pias2
2	Pigh
2	Pigk
2	Pign
2	Pigp
2	Pigq
2	Pin1
2	Pja2
2	Pkd1
2	Pkd2
2	Pkn2
2	Pknox1
2	Pkp1
2	Pla2g12a
2	Plagl2
2	Pldn
2	Plekha1
2	Plekhf2
2	Plekhh2
2	Plekhm3
2	Plin2
2	Plin4
2	Plk4
2	Plod2
2	Pltp
2	Pnn
2	Pno1
2	Pnpla2
2	Podxl
2	Poglut1
2	Pole3
2	Pole4

2	Polr1e
2	Polr3b
2	Polr3k
2	Popdc3
2	Pot1b
2	Ppapdc2
2	Ppat
2	Ppid
2	Ppig
2	Ppm1d
2	Ppm1e
2	Ppp1cb
2	Ppp1r12a
2	Ppp1r2
2	Ppp1r7
2	Ppp2ca
2	Ppp2r2d
2	Ppp2r5c
2	Ppp3cc
2	Ppp3r1
2	Ppp4r2
2	Pppde2
2	Pptc7
2	Pqlc3
2	Prdm15
2	Prep
2	Prickle3
2	Prkacb
2	Prkag2
2	Prkar1a
2	Prkar2b
2	Prkca
2	Prkcd
2	Prkci
2	Prkd3
2	Prkg2
2	Prkra
2	Prkx
2	Prmt6
2	Prmt7
2	Prpf18
2	Prpf4b
2	Prr12
2	Prr5
2	Prrc1
2	Psma3
2	Psmb1
2	Psmd10

2	Psmd4
2	Psme3
2	Pspc1
2	Psrc1
2	Ptbp2
2	Pten
2	Pter
2	Ptges3
2	Ptgr1
2	Ptk2
2	Ptp4a2
2	Ptplb
2	Ptpn13
2	Ptpre
2	Ptprk
2	Ptprm
2	Pum2
2	Pura
2	Pus10
2	Pwp1
2	Pwwp2b
2	Pxdn
2	Qk
2	Rab1
2	Rab18
2	Rab21
2	Rab27b
2	Rab35
2	Rab3ip
2	Rab5a
2	Rab8b
2	Rab9
2	Rabep1
2	Rabgef1
2	Rabggtb
2	Rac1
2	Rad18
2	Rad21
2	Rad50
2	Rad51
2	Rad51ap1
2	Rai14
2	Rala
2	Ralb
2	Ralbp1
2	Ralgapa1
2	Ranbp6
2	Ranbp9

2	Rap1gds1
2	Rap2a
2	Rapgef2
2	Rapgef6
2	Rara
2	Rasa1
2	Rasa2
2	Rbbp4
2	Rbbp5
2	Rbm18
2	Rbm20
2	Rbm22
2	Rbm34
2	Rbm45
2	Rbm7
2	Rbm8a
2	Rbms3
2	Rbpms2
2	Rc3h1
2	Rc3h2
2	Rce1
2	Rcor1
2	Rdh10
2	Recql
2	Reep3
2	Rftn1
2	Rftn2
2	Rfx7
2	Rg9mtd2
2	Rg9mtd3
2	Rheb
2	Rhob
2	Rhobtb3
2	Rhoq
2	Rhou
2	Rictor
2	Rlim
2	Rnaseh2a
2	Rnf115
2	Rnf138
2	Rnf14
2	Rnf141
2	Rnf149
2	Rnf157
2	Rnf168
2	Rnf169
2	Rnf214
2	Rnf38

2	Rnf4
2	Rnf41
2	Rnf44
2	Rnf5
2	Rngtt
2	Robo1
2	Rpl26
2	Rpl27a
2	Rpl31
2	Rpl37a
2	Rpp30
2	Rprd2
2	Rps23
2	Rps3
2	Rps6ka3
2	Rraga
2	Rras2
2	Rrm1
2	Rrp8
2	Rsc1a1
2	Rsu1
2	Rtcd1
2	Rtf1
2	Rtn4
2	Rtn4ip1
2	Runx1
2	Runx2
2	Runx3
2	Rxra
2	Ryk
2	S100pbp
2	Samd4
2	Samd8
2	Sap30l
2	Sav1
2	Sbf2
2	Scai
2	Scamp1
2	Scamp2
2	Scamp5
2	Scaper
2	Sco2
2	Scp2
2	Scyl2
2	Sdad1
2	Sec23ip
2	Sec24b
2	Sec24c

2	Secisbp2l
2	Sema3e
2	Sema4f
2	Senp1
2	Sepsecs
2	Sept10
2	Sept7
2	Serinc5
2	Serp1
2	Serpinb9
2	Serpinb9c
2	Set
2	Setd2
2	Setd3
2	Setd5
2	Setd8
2	Sf3a1
2	Sf3b2
2	Sf3b4
2	Sfrs18
2	Sgcb
2	Sgcd
2	Sh2b3
2	Sh3bgrl
2	Sh3d19
2	Sh3pxd2a
2	Sh3rf1
2	Shb
2	Shoc2
2	Shox2
2	Siah1a
2	Siah2
2	Sike1
2	Six1
2	Skap2
2	Ski
2	Skil
2	Skp1a
2	Slain2
2	Slc10a7
2	Slc11a2
2	Slc16a1
2	Slc16a11
2	Slc16a6
2	Slc1a4
2	Slc23a2
2	Slc25a11
2	Slc25a15

2	Slc25a24
2	Slc25a26
2	Slc25a30
2	Slc25a32
2	Slc25a36
2	Slc25a40
2	Slc25a46
2	Slc27a4
2	Slc2a1
2	Slc30a1
2	Slc31a1
2	Slc35a1
2	Slc35b4
2	Slc35d2
2	Slc36a1
2	Slc38a2
2	Slc39a10
2	Slc39a13
2	Slc39a6
2	Slc41a2
2	Slc4a1ap
2	Slc5a3
2	Slc6a6
2	Slc7a11
2	Slc9a3r2
2	Slc9a6
2	Slk
2	Slmap
2	Slu7
2	Smad1
2	Smad2
2	Smad4
2	Smad5
2	Smad9
2	Smap2
2	Smarcad1
2	Smarcd2
2	Smc5
2	Smpd3
2	Smurf2
2	Smyd3
2	Smyd4
2	Snrnp27
2	Snrnp48
2	Snrpb
2	Sntb1
2	Sntb2
2	Snx16

2	Snx27
2	Snx4
2	Snx6
2	Son
2	Spag9
2	Spata13
2	Spata2
2	Spata6
2	Spc24
2	Specc1
2	Spin1
2	Spire1
2	Spop
2	Spp1
2	Spred1
2	Spry2
2	Spryd4
2	Sptlc1
2	Sqstm1
2	Srek1
2	Srek1ip1
2	Sri
2	Srpk1
2	Srpk2
2	Srr
2	Srsf1
2	Srsf11
2	Srsf3
2	Ssbp3
2	Ssx2ip
2	Stag2
2	Stam
2	Stam2
2	Stard4
2	Stat3
2	Stat5b
2	Stau2
2	Stc2
2	Stk10
2	Stmn2
2	Strn
2	Stt3b
2	Stx12
2	Stx7
2	Stxbp4
2	Stxbp5
2	Styk1
2	Sufu

2	Sulf2
2	Supt16h
2	Supv3l1
2	Surf4
2	Suv420h1
2	Syap1
2	Sykb
2	Syncrip
2	Synrg
2	Sypl
2	Tab2
2	Tada1
2	Tada2a
2	Taf1
2	Taf13
2	Taf4a
2	Taf5
2	Taldo1
2	Tanc1
2	Tars
2	Tasp1
2	Tbc1d15
2	Tbcc
2	Tbcel
2	Tbl1x
2	Tbl2
2	Tbpl1
2	Tceal8
2	Tcf4
2	Tead1
2	Tecpr2
2	Tef
2	Tes
2	Tfap2a
2	Tfap4
2	Tfdp2
2	Tgfb1i1
2	Tgfbr2
2	Tgoln2
2	Thap1
2	Thap2
2	Thbd
2	Thoc3
2	Thumpd1
2	Tial1
2	Tjp1
2	Tkt
2	Tlk1

2	Tlr4
2	Tmbim1
2	Tmcc1
2	Tmed5
2	Tmed7
2	Tmed8
2	Tmeff1
2	Tmem101
2	Tmem106a
2	Tmem127
2	Tmem135
2	Tmem167b
2	Tmem168
2	Tmem170b
2	Tmem183a
2	Tmem188
2	Tmem209
2	Tmem214
2	Tmem33
2	Tmem38b
2	Tmem48
2	Tmem59
2	Tmem62
2	Tmem65
2	Tmem87b
2	Tmem95
2	Tmpo
2	Tmtc4
2	Tmx3
2	Tnfrsf1a
2	Tnks2
2	Tnrc6c
2	Tns3
2	Tomm70a
2	Tor1aip1
2	Tor1b
2	Tpbg
2	Tpd52
2	Tpp2
2	Tpt1
2	Tpx2
2	Trabd
2	Traf3
2	Traf3ip1
2	Tram2
2	Trappc6b
2	Trdmt1
2	Trib1

2	Trim16
2	Trim33
2	Trim39
2	Trim59
2	Trio
2	Trip11
2	Trip12
2	Trp2
2	Trp53inp2
2	Trpm7
2	Tsc1
2	Tsga14
2	Tsn
2	Tspan11
2	Tspan13
2	Tspan3
2	Tspan6
2	Ttbk2
2	Ttc14
2	Ttc19
2	Ttc3
2	Ttc33
2	Ttll4
2	Ttpal
2	Tubgcp4
2	Twf1
2	Twsg1
2	Txn2
2	Txnrd1
2	Tymp
2	Uba6
2	Ubac1
2	Ube2d2
2	Ube2h
2	Ube2j1
2	Ube2k
2	Ube2q2
2	Ube2w
2	Ube3c
2	Ube4b
2	Ubfd1
2	Ubox5
2	Ubr3
2	Ubr7
2	Ubxn2a
2	Ubxn4
2	Ufm1
2	Ugcg

2	Uhrf1bp1
2	Ulk1
2	Ulk2
2	Umps
2	Uqcc
2	Uqcrfs1
2	Urb2
2	Uso1
2	Usp12
2	Usp20
2	Usp37
2	Usp4
2	Usp42
2	Usp47
2	Usp48
2	Utp14b
2	Utp15
2	Vamp3
2	Vapa
2	Vbp1
2	Vcam1
2	Vcl
2	Vcpip1
2	Vdr
2	Vezf1
2	Vezt
2	Vma21
2	Vps13a
2	Vps13c
2	Vps18
2	Vps25
2	Vps26a
2	Vps4a
2	Vps4b
2	Vps54
2	Vrk1
2	Vta1
2	Wapal
2	Wasl
2	Wbp5
2	Wdfy1
2	Wdfy2
2	Wdr1
2	Wdr3
2	Wdr48
2	Wdr5
2	Wdr77
2	Wdr82

2	Wee1
2	Whsc1
2	Whsc2
2	Wipf1
2	Wisp1
2	Wls
2	Wnk1
2	Wrn
2	Wsb1
2	Wwc2
2	Xkr8
2	Xpo1
2	Xpot
2	Xpr1
2	Xrcc2
2	Xrcc4
2	Yaf2
2	Yipf3
2	Yipf4
2	Yipf5
2	Yipf6
2	Yme1l1
2	Yod1
2	Yrdc
2	Ythdc1
2	Ythdf1
2	Ywhab
2	Zbtb4
2	Zbtb42
2	Zbtb6
2	Zc3h11a
2	Zcchc2
2	Zcchc8
2	Zdhhc2
2	Zdhhc20
2	Zdhhc3
2	Zdhhc7
2	Zfand3
2	Zfhx4
2	Zfp187
2	Zfp192
2	Zfp217
2	Zfp238
2	Zfp292
2	Zfp326
2	Zfp329
2	Zfp346
2	Zfp36

2	Zfp365
2	Zfp408
2	Zfp451
2	Zfp472
2	Zfp503
2	Zfp507
2	Zfp52
2	Zfp592
2	Zfp597
2	Zfp609
2	Zfp62
2	Zfp68
2	Zfp697
2	Zfp703
2	Zfp704
2	Zfp780b
2	Zfp828
2	Zfp830
2	Zfp874b
2	Zfp937
2	Zfr
2	Zfyve1
2	Zmat2
2	Zmpste24
2	Zmym4
2	Zmynd11
2	Zmynd8
2	Znrf2
2	Zranb1
2	Zranb2
2	Zscan20
2	Zscan21
2	Zscan22
2	Zswim4
2	Zwilch
2	Zzz3
3	0610007L01Rik
3	1110003E01Rik
3	1110018J18Rik
3	1600012H06Rik
3	1700081L11Rik
3	1810074P20Rik
3	2310022B05Rik
3	2810403A07Rik
3	4732418C07Rik
3	4933439F18Rik
3	5031439G07Rik
3	6330578E17Rik

3	8030462N17Rik
3	9530068E07Rik
3	AF064781
3	AI316807
3	Acsl3
3	Actr2
3	Aebp2
3	Aes
3	Agfg1
3	Ahcyl1
3	Ahcyl2
3	Akap2
3	Alg5
3	Alkbh1
3	Amfr
3	Ammecr1l
3	Anapc1
3	Angptl2
3	Ankle2
3	Anp32b
3	Anp32e
3	Anxa4
3	Ap3s2
3	Api5
3	Apln
3	Appbp2
3	Arfgef2
3	Arl5a
3	Armc10
3	Arpc5l
3	Atp1b1
3	Atp1b3
3	Atp5f1
3	Atp6v0a2
3	Atp6v1h
3	Atxn10
3	B4galt6
3	BC031781
3	Bcat1
3	Bcl2l11
3	Bet1
3	Bves
3	C80913
3	Calm1
3	Camk2d
3	Cast
3	Ccdc88a
3	Ccdc93

3	Ccl2
3	Ccna2
3	Ccnb2
3	Cct8
3	Cd2ap
3	Cd47
3	Cd99l2
3	Cdca2
3	Cdk1
3	Cdk14
3	Cdkn2aipnl
3	Cds2
3	Cenpa
3	Cep170
3	Cep350
3	Cep76
3	Cetn3
3	Cggbp1
3	Chchd3
3	Chchd5
3	Cisd2
3	Clcn5
3	Clns1a
3	Clock
3	Cnih4
3	Cnn3
3	Col4a1
3	Col4a5
3	Col5a1
3	Creb5
3	Crem
3	Cript
3	Crnkl1
3	Csnk1d
3	Ctage5
3	Ctsb
3	Ctsl
3	Cul1
3	Cxcr4
3	Cyb5b
3	Cyhr1
3	D16Ertd472e
3	D19Bwg1357e
3	D4Bwg0951e
3	D630045J12Rik
3	Dazap1
3	Dctn5
3	Ddx17

3	Ddx21
3	Ddx46
3	Decr1
3	Dhx36
3	Dnajc18
3	Dph3
3	Dpp8
3	Dpy19l4
3	Dqx1
3	Dusp11
3	Dusp19
3	Eef2
3	Ehd1
3	Eif2s2
3	Elavl1
3	Elovl6
3	Emb
3	En1
3	Eny2
3	Epb4.1l1
3	Erlin1
3	Ets1
3	Exoc2
3	Exoc3
3	Exosc4
3	Eya3
3	Eya4
3	Fam13b
3	Fam198b
3	Fbxl20
3	Fbxw11
3	Fem1b
3	Fgfr1op2
3	Fndc3a
3	Foxp1
3	Fut11
3	G3bp2
3	Gabarapl1
3	Gabpa
3	Gatc
3	Gch1
3	Gga2
3	Glg1
3	Gmfb
3	Golga3
3	Gpr137b
3	Guf1
3	Gxylt1

3	Hells
3	Herc2
3	Hhipl1
3	Higd1a
3	Hmga2
3	Hnrnpu
3	Hsbp1
3	Hspa4
3	Htt
3	Ide
3	Idh3a
3	Ier5
3	Igf1r
3	Ing3
3	Ing5
3	Ino80c
3	Ino80e
3	Ireb2
3	Irf2bp2
3	Irs1
3	Isy1
3	Itgb1
3	Jam2
3	Jund
3	Kctd10
3	Kif1b
3	Kif2a
3	Kifap3
3	Klhdc5
3	Klraq1
3	Kpna6
3	Kras
3	Lamc1
3	Lamp2
3	Lgals1
3	Lgals8
3	Limk2
3	Lipa
3	Llph
3	Lox
3	Lrpap1
3	Lrrk2
3	Map3k7
3	Map4k4
3	Mapre1
3	Mat2b
3	Mbnl2
3	Mdp1

3	Me2
3	Metap2
3	Mff
3	Mfsd1
3	Mier3
3	Mkln1
3	Mllt11
3	Mrps5
3	Msl3
3	Mtap1b
3	Mtf1
3	Mtfr1
3	Mtmr4
3	Mudeng
3	Myh10
3	Myh9
3	Myo10
3	N4bp1
3	Nacc2
3	Nans
3	Nap1l1
3	Napg
3	Nasp
3	Nav2
3	Ncapg2
3	Ncl
3	Ndfip1
3	Ndufs4
3	Nedd4
3	Neurl1a
3	Nfkb1
3	Nmt1
3	Nmt2
3	Nob1
3	Nolc1
3	Nop58
3	Nub1
3	Nucks1
3	Nudt19
3	Opa1
3	Orc1
3	Osbpl9
3	Ostf1
3	Otud3
3	Oxct1
3	Pa2g4
3	Pacs2
3	Pafah1b1

3	Pank2
3	Pank3
3	Papd4
3	Parva
3	Pcmt1
3	Pdcd6ip
3	Pde5a
3	Pea15a
3	Pfkp
3	Phax
3	Phf6
3	Pik3r1
3	Pikfyve
3	Pitpna
3	Plekha2
3	Pmepa1
3	Pold3
3	Polk
3	Polr3e
3	Polr3f
3	Pomt2
3	Pphln1
3	Ppp1r15b
3	Ppp2cb
3	Ppp6r3
3	Pramef8
3	Prdx6
3	Prkaa1
3	Prkab2
3	Prmt1
3	Prosc
3	Prpf39
3	Prpf40a
3	Prrc2b
3	Psen1
3	Psmd12
3	Psmd9
3	Psmf1
3	Ptgs1
3	Ptpmt1
3	Ptpn14
3	Pwp2
3	Rab10
3	Rab11a
3	Rab2a
3	Rab31
3	Racgap1
3	Ralgapb

3	Ralgps2
3	Rbm25
3	Rbms2
3	Rbx1
3	Rcc2
3	Rchy1
3	Rdh11
3	Reep5
3	Rere
3	Rfk
3	Rfxap
3	Rgl1
3	Riok3
3	Rnmt
3	Rod1
3	Rpa1
3	Rpf2
3	Rpl22
3	Rpl5
3	Rpn2
3	Rrp1b
3	Rsrc1
3	Samd14
3	Samhd1
3	Sap30bp
3	Sbds
3	Sc4mol
3	Scarb2
3	Scd2
3	Scrn1
3	Sdc2
3	Sdha
3	Sec14l1
3	Sec22b
3	Sec22c
3	Sec63
3	Sephs1
3	Sept8
3	Setd7
3	Sfmbt1
3	Sgms2
3	Sh3kbp1
3	Sik2
3	Slc25a37
3	Slc26a2
3	Slc30a4
3	Slc38a6
3	Slc39a14

3	Slc39a3
3	Slc45a4
3	Slc6a8
3	Smug1
3	Smurf1
3	Snrpd3
3	Snx30
3	Snx5
3	Snx8
3	Socs4
3	Sos1
3	Spast
3	Spcs2
3	Spg20
3	Sppl3
3	Srebf2
3	Srf
3	Srp72
3	Srsf2
3	Srsf6
3	Srxn1
3	Ssh1
3	Ssr3
3	St13
3	St6galnac4
3	Steap2
3	Stk24
3	Stk35
3	Sugt1
3	Syf2
3	Tbc1d1
3	Tbl1xr1
3	Tbrg4
3	Tceb1
3	Tfrc
3	Thap7
3	Thbs1
3	Thoc4
3	Timm13
3	Timp2
3	Tiprl
3	Tm9sf2
3	Tm9sf4
3	Tmed10
3	Tmem110
3	Tmem115
3	Tmem164
3	Tmem19

3	Tmem55a
3	Tmem57
3	Tmem9b
3	Tmf1
3	Tnrc6a
3	Tpd52l2
3	Tpm1
3	Tram1
3	Trappc5
3	Trim2
3	Tsc22d1
3	Tspan5
3	Tsr2
3	Ttc39b
3	Tubb5
3	Txndc5
3	Txnl1
3	Uba5
3	Ubac2
3	Ube2e3
3	Ubl4
3	Ubtf
3	Unc119b
3	Usp15
3	Vamp2
3	Vapb
3	Vcan
3	Vim
3	Wdr26
3	Wdr91
3	Wwtr1
3	Xpnpep3
3	Xpo5
3	Yap1
3	Yeats4
3	Ythdf3
3	Ywhag
3	Yy1
3	Zbtb44
3	Zc3h15
3	Zfp207
3	Zfp598
3	Zfp706
3	Zfp91
3	Zhx3
3	Znhit3
3	Znrf1
4	0610037P05Rik

4	1110034A24Rik
4	2210012G02Rik
4	2210016L21Rik
4	2210404J11Rik
4	2310016M24Rik
4	4930455C21Rik
4	6330416G13Rik
4	9330151L19Rik
4	Add2
4	Agk
4	Angel2
4	Atp10a
4	Azi2
4	BC017158
4	BC027231
4	Cand2
4	Ccne2
4	Cdc42
4	Cdc42se2
4	Cgref1
4	Ciapin1
4	Cpne8
4	Cpt1b
4	Ctxn1
4	Cyb5r1
4	Dcun1d1
4	Ddx10
4	Dmxl1
4	Dnajc14
4	Dnajc27
4	Dnmt3a
4	Drp2
4	Eif2s1
4	Eif4e
4	Fam149b
4	Fam55c
4	Fbxo7
4	Fcgr4
4	Gne
4	Gnptab
4	Golph3
4	Gstcd
4	Hapln4
4	Hnrnpa1
4	Ifrd2
4	Ints10
4	Lin7c
4	Lrrcc1

4	Mcph1
4	Msrb3
4	Myst4
4	Nckipsd
4	Nit2
4	Pcyt2
4	Phka1
4	Plscr4
4	Prnp
4	Rab27a
4	Rnf181
4	Rock1
4	Scaf11
4	Sdhaf2
4	Serpinh1
4	Sfpq
4	Sh3glb2
4	Sh3pxd2b
4	Sms
4	Snapc1
4	Snhg4
4	Sra1
4	Srprb
4	Tank
4	Tbc1d9b
4	Tcf12
4	Terf2ip
4	Tesk1
4	Tinf2
4	Tmem134
4	Tnrc18
4	Tpm4
4	Trmt6
4	Ugp2
4	Vkorc1l1
4	Vmp1
4	Wtap
4	Zdhhc5
4	Zfp169
4	Zfp784
5	0610007P08Rik
5	1110065P20Rik
5	1500001M20Rik
5	1600014C10Rik
5	1700021F05Rik
5	1810008A18Rik
5	1810013L24Rik
5	2010012O05Rik

5	2010305A19Rik
5	2200002D01Rik
5	2310011J03Rik
5	2310033P09Rik
5	2310044G17Rik
5	2310061I04Rik
5	2410002O22Rik
5	2410131K14Rik
5	2610029I01Rik
5	2610034B18Rik
5	2810021B07Rik
5	2810055F11Rik
5	4930427A07Rik
5	5033414D02Rik
5	6230409E13Rik
5	6330503K22Rik
5	9330154J02Rik
5	9630033F20Rik
5	A230050P20Rik
5	A230051G13Rik
5	A330021E22Rik
5	AI597479
5	Aacs
5	Abhd6
5	Abi2
5	Acd
5	Aco1
5	Acot9
5	Acox1
5	Actr1b
5	Agpat3
5	Agps
5	Agtrap
5	Ahsa1
5	Akr1c14
5	Akt2
5	Alad
5	Alkbh5
5	Anapc4
5	Ank1
5	Ankrd26
5	Antxr1
5	Anxa3
5	Ap2m1
5	Ap4b1
5	Apbb1ip
5	Aptx
5	Arf4

5	Arhgap1
5	Arhgap17
5	Arhgap18
5	Arhgap28
5	Arl3
5	Armc1
5	Arpc3
5	Arsj
5	Asf1a
5	Ash2l
5	Ate1
5	Atf1
5	Atg13
5	Atg4b
5	Atic
5	Atl2
5	Atmin
5	Atp6v0c
5	Atp6v1e1
5	Atxn7l3
5	B230118H07Rik
5	B230120H23Rik
5	BC003266
5	BC067068
5	Bbx
5	Bcl10
5	Bmi1
5	Bod1l
5	Bysl
5	Bzw1
5	Cant1
5	Capn7
5	Cars
5	Cars2
5	Cbr4
5	Cbx4
5	Ccdc123
5	Ccdc41
5	Ccdc43
5	Ccdc50
5	Ccdc71
5	Ccnl1
5	Ccnt2
5	Cd109
5	Cd2bp2
5	Cda
5	Cdc123
5	Cdc42se1

5	Cdk12
5	Cdkn2c
5	Chaf1a
5	Chchd1
5	Chrna1
5	Chtf8
5	Clcn3
5	Clk4
5	Cnot10
5	Cnpy2
5	Copg
5	Cox16
5	Cox5b
5	Crls1
5	Crot
5	Ctdspl
5	Ctnnbip1
5	Ctso
5	Cwc15
5	Cx3cl1
5	D230037D09Rik
5	D3Ertd254e
5	D4Ertd22e
5	Dap3
5	Dcaf4
5	Dcaf5
5	Dctn1
5	Dcxr
5	Dda1
5	Ddah1
5	Ddit3
5	Ddit4l
5	Ddx31
5	Ddx49
5	Ddx59
5	Dennd4a
5	Denr
5	Dgcr2
5	Dlg5
5	Dlgap4
5	Dnm1
5	Dnttip2
5	Dpcd
5	Dpm2
5	Drosha
5	Dstyk
5	Dus3l
5	Dusp4

5	Ebf3
5	Ebna1bp2
5	Eci1
5	Eef1d
5	Efhd2
5	Eftud2
5	Egln2
5	Ehd2
5	Eif2c1
5	Eif4a2
5	Eif4g1
5	Eif4g3
5	Eif5a
5	Elk1
5	Elp4
5	Epc1
5	Ephb6
5	Ern1
5	Ero1l
5	Erp44
5	Esyt1
5	Faf1
5	Faf2
5	Fam162a
5	Fam188a
5	Fam35a
5	Fam53a
5	Fam53c
5	Fam54b
5	Fbxl13
5	Fgfrl1
5	Fh1
5	Foxk2
5	Foxred2
5	Frg1
5	Frk
5	Ftl1
5	Ftsj3
5	Fzd3
5	Fzd5
5	G6pdx
5	Gabpb2
5	Gapdh
5	Gatad2a
5	Gcc1
5	Gfer
5	Ggh
5	Gins3

5	Git1
5	Gm10639
5	Gm527
5	Gna13
5	Gnpat
5	Golgb1
5	Golph3l
5	Gopc
5	Gpr146
5	Gpr172b
5	Gsk3b
5	Gsta2
5	Gtpbp4
5	Gypc
5	H13
5	H1f0
5	H47
5	Haus6
5	Havcr2
5	Hcfc2
5	Hdac2
5	Hdlbp
5	Hgsnat
5	Hist1h3i
5	Hist1h4a
5	Hnrnpa2b1
5	Hoxa11
5	Hoxa7
5	Hoxa9
5	Hspa14
5	Hspb11
5	Hspe1
5	Iars2
5	Id1
5	Ier3ip1
5	Igfbp5
5	Inhbb
5	Ip6k1
5	Ipo13
5	Irak1bp1
5	Itgb1bp1
5	Itpr3
5	Jag2
5	Jak2
5	Jarid2
5	Kcnmb4
5	Kif11
5	Kitl

5	Klhl11
5	Kremen1
5	Lactb2
5	Lancl1
5	Lass6
5	Ldlrap1
5	Letm1
5	Lif
5	Loxl1
5	Loxl3
5	Lrrc28
5	Lrrc59
5	Lrrk1
5	Lrwd1
5	Lsg1
5	Lsm12
5	Lsm5
5	Lyn
5	Lyrm5
5	M6pr
5	Mak16
5	March6
5	Mast2
5	Mast3
5	Mcl1
5	Med11
5	Med16
5	Med9
5	Megf10
5	Melk
5	Mll1
5	Mll3
5	Mllt1
5	Mllt6
5	Mmachc
5	Mmgt1
5	Mr1
5	Mras
5	Mre11a
5	Mrpl14
5	Mrpl40
5	Mrpl53
5	Mrps23
5	Mrps33
5	Mrps35
5	Mta2
5	Mterfd1
5	Mthfr

5	Mvk
5	Mxi1
5	Myl12a
5	Naa38
5	Nadk
5	Naglu
5	Ncoa1
5	Ncoa6
5	Ndor1
5	Ndst1
5	Ndufaf3
5	Nedd1
5	Neurl2
5	Nfe2l1
5	Nfib
5	Nfkb2
5	Nid1
5	Nipa2
5	Nipal3
5	Noa1
5	Npcd
5	Nploc4
5	Nptx1
5	Nptxr
5	Nr1d1
5	Nubp1
5	Nup133
5	ORF61
5	Oat
5	Ogfod1
5	Orc4
5	Osbpl1a
5	Ostm1
5	Otud4
5	Otud7b
5	Pafah2
5	Paip1
5	Pak1
5	Panx1
5	Pcdh18
5	Pcyt2
5	Pdcd5
5	Pdlim1
5	Pdlim2
5	Pdpk1
5	Pdxk
5	Pgrmc2
5	Pgs1

5	Phf17
5	Phf5a
5	Phkb
5	Pitx2
5	Pkn1
5	Pla2g16
5	Plaur
5	Plcd3
5	Plekhj1
5	Plin3
5	Plscr2
5	Pnrc1
5	Pold4
5	Ppa1
5	Ppil3
5	Ppp2r2a
5	Ppp2r5e
5	Ppp4r1l-ps
5	Pppde1
5	Prc1
5	Prkrip1
5	Prmt3
5	Prpf19
5	Prpf4
5	Prps2
5	Prpsap1
5	Prr11
5	Psmc4
5	Ptbp1
5	Ptp4a1
5	Ptpra
5	Pvt1
5	Pygb
5	Qser1
5	Rab11b
5	Rab11fip5
5	Rab1b
5	Rab23
5	Rabif
5	Raly
5	Ran
5	Rap2c
5	Rassf2
5	Rbfox2
5	Rbm28
5	Rbm33
5	Rbm41
5	Rcan1

5	Rcbtb1
5	Rexo1
5	Rfc2
5	Rffl
5	Rgmb
5	Rgs12
5	Riok2
5	Ripk1
5	Rnase4
5	Rnf126
5	Rnf128
5	Rnf139
5	Rnf150
5	Rnf185
5	Rp2h
5	Rpl10
5	Rpl21
5	Rpl23
5	Rpl4
5	Rpl41
5	Rpl7a
5	Rps14
5	Rps15a
5	Rps2
5	Rps6kb2
5	Rsbn1
5	Rspry1
5	Rwdd1
5	Sars
5	Sclt1
5	Sdc4
5	Sdf4
5	Sec11a
5	Sec31a
5	Sema6a
5	Sept9
5	Sf3b1
5	Sgol1
5	Sgsm3
5	Slc25a33
5	Slc25a4
5	Slc25a43
5	Slc35e2
5	Slc39a9
5	Slc4a3
5	Slc6a17
5	Slc9a8
5	Slco3a1

5	Smarca5
5	Snrnp40
5	Snrpd2
5	Snrpe
5	Snx18
5	Snx3
5	Sqle
5	Srp68
5	Ssr2
5	St3gal1
5	Stx16
5	Suds3
5	Taf3
5	Taok1
5	Tbc1d19
5	Tbc1d2b
5	Tbx1
5	Tceb2
5	Tet3
5	Tex10
5	Tfip11
5	Thap4
5	Thoc7
5	Thumpd3
5	Timm50
5	Tjp2
5	Tm4sf1
5	Tm7sf3
5	Tm9sf1
5	Tm9sf3
5	Tmc6
5	Tmcc3
5	Tmem128
5	Tmem141
5	Tmem184b
5	Tmem194
5	Tmem206
5	Tmem5
5	Tnnt1
5	Tnpo1
5	Tomm20
5	Top1
5	Tor3a
5	Tpr
5	Tprkb
5	Traf7
5	Trim37
5	Trim44

5	Trip4
5	Trp53bp1
5	Trp53inp1
5	Tsc22d3
5	Tsfm
5	Tsnax
5	Tsr1
5	Ttc28
5	Tuba4a
5	Tubgcp2
5	Twist2
5	Txn1
5	Ube2c
5	Ube2f
5	Ube2l3
5	Ubr1
5	Ubr2
5	Ufd1l
5	Uggt1
5	Uqcrc2
5	Urm1
5	Urod
5	Usp7
5	Vdac2
5	Vegfa
5	Vhl
5	Vps53
5	Wbp4
5	Wdr13
5	Wdr18
5	Wdr37
5	Wdr4
5	Wdr76
5	Wipi1
5	Wwp2
5	Ywhae
5	Zbtb7b
5	Zcchc17
5	Zcchc9
5	Zdhhc17
5	Zdhhc21
5	Zdhhc9
5	Zfp106
5	Zfp142
5	Zfp148
5	Zfp202
5	Zfp236
5	Zfp275

5	Zfp281
5	Zfp367
5	Zfp386
5	Zfp462
5	Zfp608
5	Zkscan6
5	Zmat3
5	Znhit6
5	Zyg11b
6	Gsg2
6	Il6st
6	Ipp
6	Mrpl15
6	Tmx1
7	2810417H13Rik
7	4930444A02Rik
7	Adnp
7	Agl
7	Akt3
7	Anapc11
7	Ankrd46
7	Atf7ip
7	Atg4d
7	Avpi1
7	B3gnt1
7	BC018507
7	Baz1a
7	Bcl6b
7	Bri3
7	Brwd1
7	Btg1
7	C330006K01Rik
7	C430048L16Rik
7	Casp8
7	Ccdc101
7	Ccna2
7	Cdk5rap2
7	Crebzf
7	Ctu2
7	Dab2
7	Dpy19l3
7	Dus1l
7	Emp1
7	Eno2
7	Erf
7	Evi2b
7	Fam175a
7	Fam73b

7	Fbxl19
7	Fnip2
7	Frs2
7	Fzd7
7	Gab2
7	Galnt1
7	Gk5
7	Gm129
7	Gm5506
7	Gorab
7	Gpsm2
7	Hist1h2bg
7	Hnrnpd
7	Hnrnpul1
7	Hsdl1
7	Hsp90aa1
7	Inpp5e
7	Itgb7
7	Krcc1
7	Map3k3
7	Med29
7	Mrc2
7	Mrps17
7	Mtif3
7	Mxd1
7	Net1
7	Nhlrc3
7	Pacs1
7	Pex7
7	Phospho2
7	Polm
7	Pon2
7	Pus3
7	Rab13
7	Rasa4
7	Rbck1
7	Rfesd
7	Rfx1
7	Rhoa
7	Riok1
7	Rpl17
7	Rps24
7	Samd12
7	Sept2
7	Sfxn3
7	Smarca4
7	Smarcc2
7	Smcr8

7	Snip1
7	Srgap3
7	St3gal4
7	Stxbp1
7	Surf2
7	Suv39h2
7	Tapt1
7	Tbx3
7	Thap11
7	Tmem140
7	Tmem161a
7	Tmem175
7	Tmub2
7	Tra2a
7	Tshz3
7	Usp6nl
7	Wdr55
7	Zfp334
7	Zfp36l1
7	Zfp498
7	Zfp790
7	Zfp866
8	4831426I19Rik
8	4833439L19Rik
8	Axl
8	Bcl2l13
8	Cacna2d1
8	Cct5
8	Crcp
8	Cyb561d1
8	Dgkd
8	Dlst
8	Dpm1
8	Entpd7
8	Esyt2
8	Fbln2
8	Fbxo8
8	Fcgrt
8	Fubp1
8	Fundc2
8	Herpud2
8	Hmgb2
8	Hp1bp3
8	Ilvbl
8	Kpnb1
8	Lrrc27
8	Map3k2
8	Mdm2

8	Med12
8	Mesdc2
8	Mpi
8	Ndrg4
8	Ndufb3
8	Ndufv2
8	Nif3l1
8	Papd5
8	Papolg
8	Pcgf3
8	Pdgfc
8	Phc3
8	Plekhm1
8	Pnkp
8	Pnrc2
8	Prdx3
8	Prpf38b
8	Psmd14
8	Ptgs2
8	Ptrh1
8	Pyroxd1
8	Qdpr
8	Rnf11
8	Rplp1
8	Rrp12
8	Rwdd4a
8	Sar1a
8	Sertad2
8	Slc48a1
8	Sod2
8	Sort1
8	Sox11
8	Sp1
8	Sptlc2
8	Stau1
8	Stk11
8	Sys1
8	Tmem120a
8	Tns4
8	Tyw3
8	Uqcr10
8	Vps26b
8	Wasf2
8	Yif1a
8	Zdhhc8
8	Zfp191
9	0610010F05Rik
9	1110012D08Rik

9	1110018G07Rik
9	1110021J02Rik
9	1110049F12Rik
9	1110054O05Rik
9	1110058L19Rik
9	1200011I18Rik
9	1600002K03Rik
9	1700017B05Rik
9	1700052N19Rik
9	1810012P15Rik
9	1810063B05Rik
9	2010002N04Rik
9	2010109K11Rik
9	2210015D19Rik
9	2210016F16Rik
9	2310003F16Rik
9	2310022A10Rik
9	2310061C15Rik
9	2510003E04Rik
9	2510039O18Rik
9	2610008E11Rik
9	2610021A01Rik
9	2610044O15Rik
9	2700050L05Rik
9	2700062C07Rik
9	2700089E24Rik
9	3110001D03Rik
9	4930420K17Rik
9	4933407H18Rik
9	4933427D14Rik
9	4933428G20Rik
9	5730494M16Rik
9	5730528L13Rik
9	6530418L21Rik
9	6720456B07Rik
9	8430419L09Rik
9	9930021J03Rik
9	A430033K04Rik
9	A530013C23Rik
9	A730011L01Rik
9	AI480653
9	AU019823
9	Aak1
9	Abcb6
9	Abhd1
9	Abl1
9	Abl2
9	Acadl

9	Acads
9	Acap3
9	Acat2
9	Acin1
9	Acn9
9	Actn4
9	Actr8
9	Adam9
9	Adar
9	Adh5
9	Adi1
9	Adra1d
9	Afap1
9	Aff4
9	Afg3l2
9	Aggf1
9	Agpat1
9	Agxt2l2
9	Aip
9	Akap1
9	Akap9
9	Aldh2
9	Aldh3b1
9	Alkbh3
9	Alox5ap
9	Amacr
9	Amd1
9	Amd2
9	Amot
9	Anapc2
9	Ankmy2
9	Ankrd9
9	Anp32a
9	Antxr2
9	Ap1g2
9	Apc
9	Apc2
9	Aph1a
9	Arf1
9	Arf3
9	Arfgap2
9	Arglu1
9	Arhgap12
9	Arhgap21
9	Arid4a
9	Arl16
9	Arl6ip5
9	Arntl

9	Arv1
9	Asap3
9	Asf1b
9	Asl
9	Atad1
9	Atf6b
9	Atg7
9	Atp5b
9	Atp7a
9	Atrn
9	Atxn3
9	Auts2
9	B3gnt2
9	B4galnt1
9	BC005624
9	BC022687
9	BC026590
9	Baiap2l1
9	Banp
9	Bbip1
9	Bcl2l2
9	Bcl9l
9	Bcor
9	Bdh1
9	Becn1
9	Bet1l
9	Bid
9	Birc2
9	Bnip1
9	Bod1
9	Bola1
9	Brms1l
9	Brp44
9	Btaf1
9	Btbd6
9	Btbd7
9	Btg3
9	Btrc
9	C1qbp
9	C330019G07Rik
9	C77080
9	Camk2g
9	Camsap2
9	Car13
9	Car5b
9	Carkd
9	Carm1
9	Casc5

9	Caskin2
9	Cav1
9	Cav2
9	Cbx1
9	Cby1
9	Ccar1
9	Ccbl2
9	Ccdc126
9	Ccdc127
9	Ccdc45
9	Ccdc75
9	Ccdc90b
9	Ccnf
9	Cdc14a
9	Cdk13
9	Cdk6
9	Cenpt
9	Cenpw
9	Cep70
9	Cept1
9	Cetn2
9	Chd4
9	Chmp1b
9	Chst1
9	Chst15
9	Cinp
9	Cirh1a
9	Ckap4
9	Ckap5
9	Clasp1
9	Cldn12
9	Clk2
9	Clk3
9	Clptm1
9	Cmtm3
9	Cnbp
9	Cnih
9	Cnnm3
9	Cnot8
9	Commd6
9	Cox4nb
9	Cox6a1
9	Cpox
9	Cpsf4
9	Creb1
9	Crlf2
9	Cryz
9	Csde1

9	Csnk1g3
9	Csnk2a1
9	Cspp1
9	Cstb
9	Cstf2t
9	Cttnbp2nl
9	Cul2
9	Cwf19l2
9	Cxxc5
9	Cyr61
9	Cyth2
9	D10Wsu102e
9	D17Wsu104e
9	D19Ertd737e
9	D1Ertd622e
9	D2Ertd750e
9	D330045A20Rik
9	Dars2
9	Dbn1
9	Dcp2
9	Dcun1d2
9	Ddost
9	Ddx11
9	Ddx6
9	Dennd1a
9	Dhodh
9	Diexf
9	Dimt1
9	Dld
9	Dlg1
9	Dll1
9	Dmap1
9	Dnajc13
9	Dnajc15
9	Dnajc24
9	Dnajc25
9	Dnajc3
9	Dnajc30
9	Dnajc8
9	Dnttip1
9	Dot1l
9	Dph2
9	Dpysl2
9	Dtymk
9	Dusp10
9	Dut
9	Dzip1l
9	E2f1

9	E2f2
9	E2f4
9	E2f7
9	E430025E21Rik
9	Ebf1
9	Ece1
9	Ect2
9	Eef1a1
9	Ehmt1
9	Eif1ad
9	Eif2c2
9	Eif2c3
9	Eif3d
9	Eif4b
9	Eif4ebp2
9	Eif4enif1
9	Eif4g2
9	Elk3
9	Elof1
9	Eme2
9	Enho
9	Enpp1
9	Entpd5
9	Epb4.1l4a
9	Ephb4
9	Eps8
9	Erbb2
9	Ercc8
9	Eri1
9	Exo1
9	Exoc7
9	Exosc1
9	Exosc3
9	Extl2
9	Ezr
9	F12
9	Fadd
9	Fam105b
9	Fam161b
9	Fam171b
9	Fam173b
9	Fam188b
9	Fam193a
9	Fam203a
9	Fam3a
9	Fam40a
9	Fam54a
9	Fam63a

9	Fam69a
9	Fam86
9	Fancf
9	Farp1
9	Fblim1
9	Fbxl14
9	Fbxl17
9	Fbxo32
9	Fbxo38
9	Fbxo5
9	Fbxo6
9	Fbxw7
9	Fchsd1
9	Fgd1
9	Fhl2
9	Fkbp4
9	Fmnl3
9	Foxj2
9	Foxk1
9	Foxl1
9	Foxm1
9	Frmd8
9	Fzr1
9	G2e3
9	Gabarapl2
9	Galc
9	Galm
9	Gap43
9	Gas2l1
9	Gdf11
9	Gemin5
9	Gemin7
9	Ggta1
9	Gin1
9	Gins4
9	Gipc2
9	Gle1
9	Glipr2
9	Glo1
9	Glud1
9	Gm10762
9	Gm14378
9	Gm505
9	Gmppb
9	Gna12
9	Gnai3
9	Gnaq
9	Gng5

9	Golga7
9	Gorasp1
9	Gpatch4
9	Gpatch8
9	Gpi1
9	Gpr116
9	Gpr180
9	Gsta4
9	Gtf2a1
9	Gtf2f2
9	Gtf3c4
9	Gtf3c6
9	Gtpbp8
9	Gyk
9	Gys1
9	Gzf1
9	Hadh
9	Hadhb
9	Haus8
9	Hdac3
9	Heatr3
9	Heatr5b
9	Hecw2
9	Hemk1
9	Hexb
9	Hiatl1
9	Hilpda
9	Hmg20a
9	Hmgb1
9	Hnrnpf
9	Hnrnpr
9	Hpse
9	Hras1
9	Hrh1
9	Hs3st1
9	Hsd17b4
9	Hsf1
9	Hsp90b1
9	Hspbap1
9	Huwe1
9	Icmt
9	Id3
9	Idh1
9	Idi1
9	Ift46
9	Ift52
9	Il12rb1
9	Il17ra

9	Il18bp
9	Il4ra
9	Il7r
9	Ilf2
9	Ino80d
9	Inpp5a
9	Inpp5k
9	Insr
9	Ints1
9	Ipo11
9	Itga3
9	Itm2b
9	Itpripl2
9	Jph1
9	Kctd20
9	Kdelr3
9	Klhl20
9	Klhl28
9	Klhl30
9	Klhl5
9	Kri1
9	Krr1
9	Lars2
9	Lat2
9	Lbh
9	Leo1
9	Lgmn
9	Limk1
9	Lin37
9	Lmnb2
9	Loh12cr1
9	Lpar2
9	Lrch3
9	Lrch4
9	Lrp10
9	Lrrc16a
9	Lsm14b
9	Lss
9	Lztr1
9	Maml1
9	Maml3
9	Maoa
9	Map2k4
9	Mapk1ip1l
9	Mau2
9	Max
9	Mbnl3
9	Mccc1

9	Mcfd2
9	Mcm4
9	Mcts1
9	Med10
9	Med19
9	Med30
9	Mest
9	Metrnl
9	Mettl1
9	Mettl11a
9	Mga
9	Mina
9	Mis18bp1
9	Mki67ip
9	Mknk1
9	Mknk2
9	Mlec
9	Mllt3
9	Mllt4
9	Mlst8
9	Mospd2
9	Mphosph10
9	Mpp6
9	Mpv17
9	Mrpl19
9	Mrpl35
9	Mrpl45
9	Mrpl55
9	Mrps18c
9	Mrps30
9	Msto1
9	Mtf2
9	Mtg1
9	Mthfd2
9	Mtmr12
9	Mtmr9
9	Mum1
9	Mum1l1
9	Mylip
9	Myst1
9	Naa20
9	Naa25
9	Napa
9	Nbr1
9	Nedd9
9	Nek1
9	Nek2
9	Neu1

9	Nfe2l2
9	Nfkbiz
9	Nicn1
9	Nip7
9	Nlgn2
9	Nme2
9	Nmnat2
9	Nnt
9	Npat
9	Npr3
9	Nqo1
9	Nrbp2
9	Nsmaf
9	Ntn1
9	Nucb2
9	Nudcd3
9	Nudt1
9	Nup50
9	Nupl2
9	Ogfod2
9	Olfm1
9	Optn
9	Oraov1
9	Orc2
9	Ormdl2
9	Os9
9	Osbp
9	Osbpl10
9	Osbpl11
9	Osr1
9	P2ry2
9	Pacrgl
9	Pafah1b2
9	Paip2b
9	Pask
9	Paxip1
9	Pcbp2
9	Pcgf5
9	Pcif1
9	Pcyt1a
9	Pde10a
9	Pde3b
9	Pdhb
9	Pdk3
9	Pdlim3
9	Pex3
9	Pfdn1
9	Pfdn5

9	Pgp
9	Pgpep1
9	Phf23
9	Pigv
9	Pigy
9	Pip4k2a
9	Pja1
9	Plekha3
9	Plekhh1
9	Plk2
9	Plxna1
9	Pnpt1
9	Pofut1
9	Polg
9	Polh
9	Polr1c
9	Polr2f
9	Polr3c
9	Pom121
9	Pon3
9	Pou2f1
9	Ppfibp1
9	Ppm1b
9	Ppp1r11
9	Ppp1r9b
9	Prdm2
9	Prkrir
9	Pros1
9	Prps1l3
9	Psip1
9	Psma1
9	Psmc5
9	Psme4
9	Ptp4a3
9	Ptpn21
9	Ptpn3
9	Ptprd
9	Ptprg
9	Ptprn
9	Pts
9	Pum1
9	Pxmp3
9	RP23-3M10.7
9	Rab2b
9	Rabl5
9	Rad1
9	Rad23b
9	Rad9

9	Raet1e
9	Raf1
9	Ranbp10
9	Rap1b
9	Rbbp7
9	Rbm17
9	Rbm26
9	Rbm5
9	Rbms1
9	Rcn1
9	Rdbp
9	Rdx
9	Refbp2
9	Rel
9	Rem1
9	Rfx3
9	Rgs19
9	Rhbdd1
9	Rhbdd2
9	Rhobtb2
9	Rhot1
9	Rhot2
9	Rin3
9	Ripk2
9	Rnf34
9	Rnf6
9	Rnf8
9	Rpl28
9	Rpl37
9	Rpl7l1
9	Rplp0
9	Rpp40
9	Rprd1b
9	Rps12
9	Rps3a
9	Rps6kb1
9	Rpsa
9	Rrbp1
9	Ruvbl2
9	Saal1
9	Samd4b
9	Samm50
9	Sap18
9	Sardh
9	Sash1
9	Sc5d
9	Sccpdh
9	Sdc1

9	Sec23a
9	Sec61b
9	Sema3b
9	Serpinb9b
9	Sertad1
9	Setdb1
9	Sgk1
9	Sgpl1
9	Sh2d1b1
9	Sh3bp2
9	Sh3bp4
9	Sh3rf3
9	Shc1
9	Shf
9	Shisa5
9	Shroom3
9	Sik1
9	Sik3
9	Sin3a
9	Sipa1l1
9	Sirt5
9	Sirt6
9	Ska1
9	Skp2
9	Slc19a1
9	Slc19a2
9	Slc20a2
9	Slc25a3
9	Slc30a5
9	Slc31a2
9	Slc33a1
9	Slc35a5
9	Slc35e1
9	Slc35f5
9	Slc37a2
9	Slc44a2
9	Slc7a6
9	Slx4
9	Smarcal1
9	Smcr7l
9	Smu1
9	Smyd5
9	Snn
9	Snx13
9	Socs5
9	Socs6
9	Sorbs3
9	Spnb2

9	Spry4
9	Srd5a3
9	Srp14
9	Srsf7
9	Stk17b
9	Stk39
9	Stom
9	Stx11
9	Sucla2
9	Sumf2
9	Syde1
9	Sync
9	Synj2bp
9	Taf12
9	Taf1d
9	Taf5l
9	Tax1bp3
9	Tbc1d10b
9	Tbc1d22b
9	Tbc1d23
9	Tbca
9	Tdp2
9	Tead3
9	Tfb1m
9	Tfg
9	Them4
9	Timm10
9	Timm22
9	Timm9
9	Tipin
9	Tlr3
9	Tmbim6
9	Tmem106b
9	Tmem123
9	Tmem126b
9	Tmem17
9	Tmem18
9	Tmem2
9	Tmem231
9	Tmem39a
9	Tmem68
9	Tmem9
9	Tmem98
9	Tnfaip1
9	Tnfaip2
9	Tnfrsf22
9	Tnfsf13
9	Tnnt2

9	Tnpo3
9	Toe1
9	Tollip
9	Tomm22
9	Tomm40l
9	Tox4
9	Tpm3
9	Tram1l1
9	Trim25
9	Trim32
9	Trim35
9	Trim41
9	Trim65
9	Trip13
9	Trpc4ap
9	Trub1
9	Tsta3
9	Ttc1
9	Ttc39a
9	Ttc4
9	Ttll1
9	Tusc1
9	Twistnb
9	Txndc17
9	Txndc9
9	Txnl4a
9	Uaca
9	Uap1
9	Uap1l1
9	Ube2a
9	Ube2d3
9	Ube2n
9	Ube2o
9	Ube2q1
9	Ubn2
9	Ubr4
9	Ubr5
9	Ubxn7
9	Uchl5
9	Uck2
9	Usp1
9	Usp11
9	Usp22
9	Usp38
9	Usp53
9	Vash1
9	Vps11
9	Vps33a

9	Vps37b
9	Wac
9	Wdr43
9	Wdr44
9	Wdr45l
9	Wdr89
9	Whsc1l1
9	Ybx1
9	Zbtb10
9	Zbtb37
9	Zbtb39
9	Zc3h7b
9	Zcchc10
9	Zdhhc6
9	Zeb1
9	Zfand6
9	Zfp119a
9	Zfp2
9	Zfp26
9	Zfp276
9	Zfp280c
9	Zfp30
9	Zfp319
9	Zfp322a
9	Zfp330
9	Zfp397
9	Zfp398
9	Zfp513
9	Zfp516
9	Zfp532
9	Zfp593
9	Zfp629
9	Zfp639
9	Zfp64
9	Zfp644
9	Zfp719
9	Zfp72
9	Zfp740
9	Zfp84
9	Zfp869
9	Zfp9
9	Zfyve20
9	Zfyve9
9	Zkscan17
9	Zmiz2
10	0610009D07Rik
10	0610031J06Rik
10	1110002E22Rik

10	1110008F13Rik
10	1110014N23Rik
10	1110020G09Rik
10	1110037F02Rik
10	1110038F14Rik
10	1110051M20Rik
10	1110057K04Rik
10	1110059G10Rik
10	1200014J11Rik
10	1300010F03Rik
10	1300018I17Rik
10	1500015A07Rik
10	1500032L24Rik
10	1520402A15Rik
10	1600002H07Rik
10	1600012F09Rik
10	1700029F09Rik
10	1700030J22Rik
10	1700034H14Rik
10	1700049G17Rik
10	1700094D03Rik
10	1700096K18Rik
10	1700123O20Rik
10	1810011O10Rik
10	1810020D17Rik
10	1810026J23Rik
10	1810027O10Rik
10	1810029B16Rik
10	1810043H04Rik
10	2010011I20Rik
10	2010106G01Rik
10	2010107E04Rik
10	2010317E24Rik
10	2010320M18Rik
10	2010321M09Rik
10	2210009G21Rik
10	2210018M11Rik
10	2210411K11Rik
10	2300009A05Rik
10	2310003C23Rik
10	2310003H01Rik
10	2310003L22Rik
10	2310004I24Rik
10	2310008H09Rik
10	2310009B15Rik
10	2310030N02Rik
10	2310044H10Rik
10	2310079F23Rik

10	2410001C21Rik
10	2410015M20Rik
10	2410017P09Rik
10	2500003M10Rik
10	2510006D16Rik
10	2610019F03Rik
10	2610021K21Rik
10	2610027L16Rik
10	2610029G23Rik
10	2610039C10Rik
10	2610301G19Rik
10	2610524H06Rik
10	2700007P21Rik
10	2700081O15Rik
10	2810001G20Rik
10	2810002N01Rik
10	2810046L04Rik
10	2810453I06Rik
10	3110001I22Rik
10	3110002H16Rik
10	3110040N11Rik
10	3110062M04Rik
10	4632411B12Rik
10	4632419I22Rik
10	4833403I15Rik
10	4921513D23Rik
10	4930503L19Rik
10	4930547N16Rik
10	4930579C15Rik
10	4931408A02Rik
10	4932438A13Rik
10	4933407C03Rik
10	4933411K20Rik
10	4933421E11Rik
10	4933424B01Rik
10	4933426M11Rik
10	4933433P14Rik
10	5133401N09Rik
10	5730455P16Rik
10	5730508B09Rik
10	5730590G19Rik
10	5830418K08Rik
10	5830433M19Rik
10	6030419C18Rik
10	6030445D17Rik
10	6330409N04Rik
10	6530401N04Rik
10	8430406I07Rik

10	9030612E09Rik
10	9030617O03Rik
10	9030624J02Rik
10	9130011E15Rik
10	9130011J15Rik
10	9130023H24Rik
10	9330159F19Rik
10	9330161L09Rik
10	9430015G10Rik
10	9430038I01Rik
10	A130010J15Rik
10	A230046K03Rik
10	A530054K11Rik
10	A630033H20Rik
10	A630038E17Rik
10	A630072M18Rik
10	A830007P12Rik
10	A830010M20Rik
10	A830080D01Rik
10	AA987161
10	AI314180
10	AI462493
10	AI464131
10	AI846148
10	AU018091
10	AU022252
10	AW549877
10	Aagab
10	Aarsd1
10	Abca8b
10	Abcb1b
10	Abcb8
10	Abcc1
10	Abcc5
10	Abcd3
10	Abcf3
10	Abcg2
10	Abi1
10	Acaa1a
10	Acaa2
10	Acad12
10	Acadm
10	Acadsb
10	Acadvl
10	Acat1
10	Acbd3
10	Acbd5
10	Aco2

10	Acot8
10	Acpl2
10	Acsbg1
10	Actr10
10	Actr3b
10	Actr5
10	Adam10
10	Adamts4
10	Adamtsl5
10	Adat2
10	Adc
10	Adcy9
10	Add1
10	Adh7
10	Adipor2
10	Adk
10	Adora2b
10	Adpgk
10	Adssl1
10	Aebp1
10	Agap1
10	Agpat5
10	Agpat9
10	Ahcy
10	Ahi1
10	Ahr
10	Ahrr
10	Ahsa2
10	Aig1
10	Ak1
10	Akap6
10	Akirin1
10	Akr1b10
10	Aldh1a3
10	Aldh7a1
10	Aldh9a1
10	Alg13
10	Alg2
10	Alg3
10	Alg9
10	Alkbh8
10	Alox5
10	Alpk1
10	Ambra1
10	Amigo2
10	Amn1
10	Amz2
10	Anapc13

10	Anapc16
10	Anapc7
10	Angpt2
10	Ank3
10	Ankra2
10	Ankrd12
10	Ankrd17
10	Ankrd39
10	Ankrd52
10	Anks1
10	Anks6
10	Ano8
10	Anubl1
10	Anxa6
10	Anxa9
10	Ap2b1
10	Ap3b1
10	Ap3m2
10	Ap3s1
10	Ap4m1
10	Apaf1
10	Apitd1
10	Aplf
10	Apobec1
10	Apobec3
10	Apol10b
10	Apol8
10	Apon
10	Aqr
10	Areg
10	Arfgap1
10	Arfgap3
10	Arfgef1
10	Arfrp1
10	Arhgap23
10	Arhgap31
10	Arhgap39
10	Arhgap42
10	Arhgap44
10	Arhgdib
10	Arhgef10
10	Arhgef11
10	Arhgef12
10	Arhgef18
10	Arhgef40
10	Arid1b
10	Arih1
10	Arl1

10	Armc7
10	Arpc1b
10	Arpc5
10	Arrb1
10	Arrdc3
10	Arsb
10	Arsk
10	Arvcf
10	Asah2
10	Asap2
10	Asb1
10	Asb7
10	Ascc3
10	Asns
10	Asnsd1
10	Asrgl1
10	Ass1
10	Asxl2
10	Atf6
10	Atg10
10	Atg16l2
10	Atg2b
10	Atg4a
10	Atg4c
10	Atp11b
10	Atp2b4
10	Atp5g1
10	Atp5l
10	Atp5s
10	Atp6v0d2
10	Atp9b
10	Atpaf1
10	Atxn1
10	Atxn7
10	Atxn7l3b
10	Aurka
10	Aurkb
10	Aven
10	Avpr1a
10	B230208H17Rik
10	B230312A22Rik
10	B2m
10	B330016D10Rik
10	B3galnt2
10	B3gntl1
10	B4galt2
10	B4galt5
10	B930059L03Rik

10	B9d2
10	BC003331
10	BC016423
10	BC016495
10	BC017647
10	BC030336
10	BC031353
10	BC046331
10	BC048355
10	BC052040
10	BC096441
10	Baat1
10	Bach1
10	Bag1
10	Bag2
10	Bag5
10	Baz1b
10	Bcap29
10	Bckdk
10	Bcl9
10	Bfar
10	Bicd1
10	Birc6
10	Blvrb
10	Bmp2k
10	Bnc2
10	Bpgm
10	Bptf
10	Brd1
10	Brd9
10	Brf2
10	Brpf3
10	Brsk1
10	Bsdc1
10	Bsn
10	Btbd9
10	Btf3l4
10	Bub1
10	Bud13
10	Bzw2
10	C130022K22Rik
10	C130039O16Rik
10	C1qtnf1
10	C1qtnf3
10	C2cd2
10	C2cd2l
10	C330018D20Rik
10	C530008M17Rik

10	C920009B18Rik
10	Cab39l
10	Cacnb2
10	Cad
10	Cadm4
10	Calcrl
10	Calu
10	Camk1d
10	Camk2a
10	Camkk2
10	Camsap1
10	Cap1
10	Capns1
10	Capza1
10	Car2
10	Casc4
10	Casd1
10	Casp12
10	Casp3
10	Casp6
10	Casp7
10	Casp8ap2
10	Casp9
10	Cbfa2t2
10	Cbl
10	Cbln1
10	Cbwd1
10	Cbx7
10	Ccdc102a
10	Ccdc104
10	Ccdc112
10	Ccdc117
10	Ccdc122
10	Ccdc130
10	Ccdc132
10	Ccdc14
10	Ccdc21
10	Ccdc34
10	Ccdc47
10	Ccdc51
10	Ccdc53
10	Ccdc68
10	Ccdc72
10	Ccdc74a
10	Ccdc76
10	Ccdc99
10	Ccnc
10	Ccnd1

10	Ccnd2
10	Cd200
10	Cd28
10	Cd320
10	Cd3eap
10	Cd59a
10	Cd63
10	Cd81
10	Cd82
10	Cd97
10	Cdan1
10	Cdc16
10	Cdc25a
10	Cdc42ep3
10	Cdc6
10	Cdc7
10	Cdc73
10	Cdh13
10	Cdk16
10	Cdk18
10	Cdk5rap3
10	Cdk8
10	Cebpg
10	Cenpc1
10	Cenpo
10	Cep110
10	Cep164
10	Cep290
10	Cep55
10	Cep63
10	Cep97
10	Ces1g
10	Cfl1
10	Cflar
10	Cfp
10	Cgnl1
10	Chd6
10	Cherp
10	Chic1
10	Chic2
10	Chid1
10	Chmp2a
10	Chmp4c
10	Chpt1
10	Chrnb1
10	Chrnd
10	Chst11
10	Chst12

10	Chst14
10	Chst7
10	Cisd1
10	Ckap2
10	Clasp2
10	Clcn6
10	Clec16a
10	Clk1
10	Clmn
10	Cln3
10	Cln6
10	Clpx
10	Cltc
10	Cmas
10	Cmklr1
10	Cmpk1
10	Cmtm4
10	Cnn2
10	Cnot1
10	Cnot6
10	Cnot6l
10	Cntfr
10	Cntln
10	Cntnap1
10	Col18a1
10	Col4a3bp
10	Colec12
10	Commd2
10	Commd3
10	Commd5
10	Comt
10	Comtd1
10	Copb1
10	Copg2
10	Cops7a
10	Cops7b
10	Cops8
10	Coq5
10	Coq6
10	Coro1c
10	Coro7
10	Cort
10	Cox11
10	Cox19
10	Cr1l
10	Crbn
10	Creb3l2
10	Creg1

10	Crocc
10	Cry1
10	Cryab
10	Cryzl1
10	Cs
10	Csad
10	Cse1l
10	Csk
10	Csnk1g2
10	Cspg4
10	Ctbs
10	Ctdnep1
10	Ctdspl2
10	Ctgf
10	Cth
10	Ctnnal1
10	Ctnnbl1
10	Ctns
10	Ctr9
10	Cul3
10	Cul5
10	Cwc22
10	Cwc25
10	Cwc27
10	Cwf19l1
10	Cxadr
10	Cyb5
10	Cyb561d2
10	Cyc1
10	Cycs
10	Cyp20a1
10	Cyp4v3
10	D11Wsu99e
10	D14Abb1e
10	D17H6S56E-5
10	D18Ertd653e
10	D2Wsu81e
10	D330012F22Rik
10	D330028D13Rik
10	D330050I16Rik
10	D630037F22Rik
10	D6Wsu163e
10	D930014E17Rik
10	Dag1
10	Dars
10	Daxx
10	Dbf4
10	Dbndd2

10	Dbp
10	Dbr1
10	Dcaf17
10	Dcaf6
10	Dclk1
10	Dclre1b
10	Dclre1c
10	Dcp1a
10	Dctn4
10	Dctn6
10	Dcun1d3
10	Ddhd2
10	Ddrgk1
10	Ddx19a
10	Ddx19b
10	Ddx39
10	Ddx42
10	Ddx50
10	Ddx52
10	Ddx54
10	Ddx55
10	Ddx56
10	Dedd
10	Def6
10	Depdc1b
10	Depdc5
10	Dgat2
10	Dgcr14
10	Dgcr6
10	Dgke
10	Dhdds
10	Dhrs7
10	Dhrs9
10	Dhx15
10	Dhx16
10	Dhx32
10	Dhx33
10	Dhx34
10	Dhx35
10	Dhx37
10	Dhx57
10	Diap2
10	Diap3
10	Dip2a
10	Dip2b
10	Dip2c
10	Dis3
10	Dlgap5

10	Dmrt2
10	Dmwd
10	Dnaja1
10	Dnaja4
10	Dnajb12
10	Dnajb14
10	Dnajb2
10	Dnajb4
10	Dnajb6
10	Dnajb9
10	Dnajc1
10	Dnajc11
10	Dnajc12
10	Dnajc7
10	Dock7
10	Dock8
10	Dock9
10	Donson
10	Dpagt1
10	Drg2
10	Dse
10	Dsn1
10	Dstn
10	Dtl
10	Dtna
10	Dtnb
10	Dtwd2
10	Dtx4
10	Dusp3
10	Dusp8
10	Dyrk1b
10	E2f5
10	E2f8
10	E330016A19Rik
10	Ears2
10	Echdc3
10	Edf1
10	Edil3
10	Eea1
10	Eef1e1
10	Eef1g
10	Eef2k
10	Efcab2
10	Efemp2
10	Efhd1
10	Efna4
10	Efnb1
10	Efr3a

10	Egfr
10	Egln1
10	Egr1
10	Egr3
10	Ehd3
10	Ei24
10	Eid2b
10	Eid3
10	Eif1ax
10	Eif1b
10	Eif2a
10	Eif2ak1
10	Eif2ak2
10	Eif2ak4
10	Eif2b1
10	Eif3j
10	Eif4a1
10	Eif4ebp1
10	Eif5b
10	Ell2
10	Elmo2
10	Eml1
10	Eml5
10	Endod1
10	Eno1
10	Ep300
10	Ep400
10	Epb4.1l5
10	Epc2
10	Epdr1
10	Epn2
10	Erc1
10	Ereg
10	Eri2
10	Errfi1
10	Esco1
10	Etfa
10	Evi2a
10	Evi5
10	Ewsr1
10	Exoc1
10	Exoc6b
10	Exog
10	Exosc2
10	Exosc6
10	Extl3
10	Ezh2
10	F2r

10	F3
10	F630043A04Rik
10	Fabp5
10	Fahd1
10	Fam100b
10	Fam102b
10	Fam110a
10	Fam113a
10	Fam114a1
10	Fam116a
10	Fam116b
10	Fam118b
10	Fam122a
10	Fam125a
10	Fam129a
10	Fam134c
10	Fam136a
10	Fam13c
10	Fam151b
10	Fam160a1
10	Fam164c
10	Fam168a
10	Fam168b
10	Fam172a
10	Fam174a
10	Fam176b
10	Fam178a
10	Fam192a
10	Fam19a5
10	Fam32a
10	Fam36a
10	Fam45a
10	Fam49b
10	Fam5c
10	Fam82a1
10	Fam84b
10	Fancl
10	Fank1
10	Farp2
10	Fastkd2
10	Fastkd3
10	Fau
10	Fbln1
10	Fbln7
10	Fbrsl1
10	Fbxl2
10	Fbxl8
10	Fbxo11

10	Fbxo18
10	Fbxo34
10	Fbxo4
10	Fbxo46
10	Fcho2
10	Fdft1
10	Fgd6
10	Fgf10
10	Fhdc1
10	Ficd
10	Fignl1
10	Filip1l
10	Fip1l1
10	Fkbp14
10	Fkbp15
10	Fkbp2
10	Fkrp
10	Fktn
10	Flad1
10	Flcn
10	Fnbp1l
10	Fndc4
10	Foxc1
10	Foxn3
10	Foxp4
10	Foxs1
10	Frat2
10	Fryl
10	Fstl1
10	Ftsjd1
10	Fuca1
10	Fuca2
10	Fut10
10	Fut8
10	Fxc1
10	Fxn
10	Fyn
10	Fzd8
10	Gadd45a
10	Gadd45b
10	Gale
10	Galns
10	Galnt10
10	Galnt4
10	Gamt
10	Gan
10	Ganab
10	Gata3

10	Gba2
10	Gcat
10	Gcdh
10	Gdnf
10	Gem
10	Gfod1
10	Gfod2
10	Gfra1
10	Gga3
10	Ggps1
10	Gigyf2
10	Gins2
10	Gjb4
10	Gli3
10	Glis2
10	Glis3
10	Glmn
10	Glod4
10	Glt25d1
10	Glt8d2
10	Gltpd1
10	Gltscr1
10	Glul
10	Gm14443
10	Gm15427
10	Gm19589
10	Gm3219
10	Gm3258
10	Gm4737
10	Gm4944
10	Gm5801
10	Gm608
10	Gm889
10	Gm9776
10	Gm9846
10	Gmip
10	Gmpr
10	Gnai1
10	Gnao1
10	Gnb1l
10	Gnb5
10	Gng12
10	Gng2
10	Gnpnat1
10	Golga1
10	Gp1bb
10	Gpam
10	Gpbp1l1

10	Gpc6
10	Gpnmb
10	Gpr107
10	Gpr108
10	Gpr124
10	Gpr125
10	Gpr19
10	Gpr39
10	Gpr89
10	Gpr97
10	Gprasp1
10	Gprc5b
10	Gprin3
10	Gps1
10	Gpt
10	Gramd1b
10	Gramd3
10	Gramd4
10	Grhpr
10	Grinl1a
10	Grinl1a
10	Grpel2
10	Grsf1
10	Grtp1
10	Gse1
10	Gspt1
10	Gspt2
10	Gsto1
10	Gstz1
10	Gtf2e2
10	Gtf2h1
10	Gtf2h2
10	Gtf2h3
10	Gtf2i
10	Gtpbp1
10	Gyg
10	H2afv
10	H2afx
10	H2afz
10	H3f3a
10	H6pd
10	Hace1
10	Hao1
10	Harbi1
10	Has2
10	Hat1
10	Hbp1
10	Hbs1l

10	Hcfc1r1
10	Hdac4
10	Hdhd2
10	Hdhd3
10	Hebp2
10	Hectd3
10	Heg1
10	Helb
10	Helq
10	Hes1
10	Hexim2
10	Hfe
10	Hibch
10	Hif1an
10	Hint3
10	Hip1
10	Hipk1
10	Hirip3
10	Hist1h1c
10	Hist1h2ae
10	Hist1h2ag
10	Hist1h2ah
10	Hist1h2ai
10	Hist1h2be
10	Hist1h3g
10	Hist1h4h
10	Hist1h4i
10	Hist1h4k
10	Hist2h3c1
10	Hist2h3c2-ps
10	Hivep2
10	Hk1
10	Hlx
10	Hmx2
10	Hn1l
10	Hnrnpc
10	Hnrnph3
10	Hnrpll
10	Hoga1
10	Homez
10	Hook2
10	Hoxc4
10	Hprt
10	Hps5
10	Hps6
10	Hs2st1
10	Hs6st1
10	Hsd17b7

10	Hsdl2
10	Hsp90ab1
10	Hspa12a
10	Hspb6
10	Hspd1
10	Hsph1
10	Htatip2
10	Htr1d
10	Ica1l
10	Iffo1
10	Ifi202b
10	Ifi35
10	Ifih1
10	Ifit1
10	Ifitm2
10	Ifnar2
10	Ift57
10	Ift81
10	Igbp1
10	Igfbp2
10	Ighmbp2
10	Igsf3
10	Ikbip
10	Ikzf5
10	Il10rb
10	Il17d
10	Il18rap
10	Il1rn
10	Ildr2
10	Ilk
10	Impa1
10	Inf2
10	Ing1
10	Ino80b
10	Inpp1
10	Inpp5d
10	Inpp5f
10	Inppl1
10	Ints12
10	Ints3
10	Ints8
10	Invs
10	Ipmk
10	Ipo4
10	Ipo8
10	Ippk
10	Iqcc
10	Iqcg

10	Iqcj-schip1
10	Iqgap3
10	Iqsec1
10	Irak1
10	Irak2
10	Irak4
10	Irf2
10	Irs2
10	Isg20l2
10	Isl2
10	Isoc2a
10	Itch
10	Itfg1
10	Itfg2
10	Itga7
10	Itgb2
10	Itgb8
10	Itpr2
10	Ivd
10	Jagn1
10	Jkamp
10	Jmjd4
10	Jmjd8
10	Junb
10	Kank2
10	Kat2b
10	Katnb1
10	Kbtbd10
10	Kbtbd4
10	Kcnab1
10	Kcnc2
10	Kcnh2
10	Kctd18
10	Kctd3
10	Kdelc1
10	Kdelc2
10	Kdm2a
10	Kdm3a
10	Kdm4a
10	Kdm4b
10	Kdm5a
10	Kdsr
10	Keap1
10	Khk
10	Khnyn
10	Khsrp
10	Kidins220
10	Kif13b

10	Kif1a
10	Kif1c
10	Kif23
10	Kif24
10	Kif3b
10	Kif3c
10	Kif5b
10	Kif7
10	Kifc1
10	Kifc3
10	Kifc5b
10	Klf16
10	Klf9
10	Klhdc1
10	Klhdc8a
10	Klhl12
10	Klhl17
10	Klhl2
10	Klhl23
10	Klhl26
10	Klhl36
10	Klhl7
10	Klrg2
10	Krt10
10	Krt80
10	Ksr1
10	L2hgdh
10	L3mbtl2
10	L3mbtl3
10	Lamtor3
10	Lap3
10	Larp1
10	Larp1b
10	Larp4b
10	Layn
10	Lbr
10	Lcorl
10	Ldb1
10	Ldha
10	Ldlrad3
10	Lenep
10	Leng1
10	Leprel4
10	Letmd1
10	Lgr4
10	Lifr
10	Lima1
10	Lin54

10	Lin9
10	Lins
10	Lipt2
10	Llgl1
10	Lman1
10	Lman2
10	Lmf1
10	Lmo4
10	Lmtk2
10	Lnpep
10	Lnx2
10	Lonp1
10	Lonrf1
10	Loxl2
10	Lpar1
10	Lpar6
10	Lpin1
10	Lpl
10	Lrch2
10	Lrch4-sap25
10	Lrig2
10	Lrig3
10	Lrrc39
10	Lrrc41
10	Lrrc4c
10	Lrrc8c
10	Lsm14a
10	Lsm6
10	Lsmd1
10	Lsp1
10	Lta4h
10	Ltv1
10	Luc7l2
10	Ly75
10	Lynx1
10	Lyplal1
10	Lyrm2
10	Lysmd3
10	Lysmd4
10	Lzic
10	Lzts2
10	Macf1
10	Mad2l1
10	Madd
10	Maf1
10	Mafg
10	Mafk
10	Magi1

10	Magi3
10	Magohb
10	Malt1
10	Man1b1
10	Man2b2
10	Map2k1
10	Map3k12
10	Map3k9
10	Mapk4
10	Mapkapk2
10	Mapkbp1
10	March7
10	March9
10	Mark2
10	Mastl
10	Mbd5
10	Mboat1
10	Mbtd1
10	Mbtps2
10	Mcart1
10	Mcm5
10	Mcm8
10	Mcmbp
10	Mctp2
10	Meaf6
10	Med14
10	Med15
10	Med18
10	Med20
10	Med7
10	Med8
10	Mef2a
10	Mef2d
10	Megf9
10	Meis2
10	Mep1a
10	Mertk
10	Metrn
10	Mettl10
10	Mettl16
10	Mettl21a
10	Mfap1a
10	Mfap1b
10	Mfap3
10	Mfn2
10	Mfsd11
10	Mfsd3
10	Mfsd8

10	Mgat3
10	Mib1
10	Midn
10	Minpp1
10	Mkks
10	Mlf1ip
10	Mlh1
10	Mlh3
10	Mlkl
10	Mll2
10	Mlx
10	Mmaa
10	Mmp24
10	Mms19
10	Mns1
10	Mob3a
10	Mocs2
10	Mocs3
10	Mon1b
10	Morc4
10	Morf4l1
10	Mpg
10	Mphosph6
10	Mphosph8
10	Mphosph9
10	Mrfap1
10	Mrgprf
10	Mrpl1
10	Mrpl10
10	Mrpl11
10	Mrpl30
10	Mrpl32
10	Mrpl36
10	Mrpl43
10	Mrpl46
10	Mrpl49
10	Mrpl51
10	Mrps2
10	Mrps25
10	Mrps31
10	Mrps36
10	Mrps7
10	Mrps9
10	Mtap1a
10	Mtap4
10	Mthfsd
10	Mtmr1
10	Mtmr10

10	Mtmr14
10	Mtmr2
10	Mtrf1l
10	Mtss1
10	Mtss1l
10	Mtx3
10	Mxd3
10	Mxd4
10	Myl12b
10	Mylk4
10	Myo18a
10	Myo6
10	Myt1
10	N4bp3
10	Naa35
10	Naaa
10	Nab2
10	Nacc1
10	Naif1
10	Nap1l4
10	Napb
10	Naprt1
10	Narfl
10	Narg2
10	Nat10
10	Nav1
10	Nav3
10	Ncapg
10	Ncbp2
10	Nckap1l
10	Ncln
10	Ncoa4
10	Ncoa5
10	Nde1
10	Ndnl2
10	Ndst4
10	Ndufa11
10	Ndufa3
10	Ndufa9
10	Ndufab1
10	Nek4
10	Nek7
10	Nek9
10	Nemf
10	Nfatc1
10	Nfatc2ip
10	Nfic
10	Nfix

10	Nfkbie
10	Nfs1
10	Nfu1
10	Nfxl1
10	Nfyc
10	Ngfrap1
10	Ngly1
10	Nhlrc2
10	Nhp2l1
10	Nipa1
10	Nipsnap1
10	Nme3
10	Nmnat1
10	Nmral1
10	Nod2
10	Nog
10	Nol11
10	Nol3
10	Nol7
10	Nol8
10	Nosip
10	Npc1
10	Nr1d2
10	Nr2f1
10	Nr2f6
10	Nr3c1
10	Nrg1
10	Nrip1
10	Nrp1
10	Nsa2
10	Nsd1
10	Nsdhl
10	Nsf
10	Nsg1
10	Nsmce1
10	Nsmce4a
10	Nsun4
10	Nsun5
10	Nsun6
10	Nt5dc1
10	Ntpcr
10	Nuak1
10	Nubpl
10	Nucb1
10	Nudt14
10	Nudt17
10	Nudt18
10	Nudt2

10	Nudt5
10	Numb
10	Nup37
10	Nup98
10	Nusap1
10	Nvl
10	ORF19
10	Oaf
10	Obfc2a
10	Odf2l
10	Odz3
10	Ogdh
10	Ogn
10	Olfml2b
10	Opa3
10	Orai1
10	Orai2
10	Orai3
10	Orc6
10	Osbpl2
10	Oscp1
10	Osr1
10	Ostc
10	Otud6b
10	P2rx4
10	P2rx5
10	P4ha1
10	Pabpc1
10	Pacrg
10	Paf1
10	Pag1
10	Paics
10	Pak1ip1
10	Palm2
10	Pan3
10	Paqr4
10	Paqr7
10	Parg
10	Parp3
10	Parp8
10	Patl1
10	Pbk
10	Pbx1
10	Pbx2
10	Pbx3
10	Pbx4
10	Pcbd2
10	Pcdh19

10	Pcdhb16
10	Pcdhb18
10	Pcdhb21
10	Pck2
10	Pcm1
10	Pcnt
10	Pcsk9
10	Pcx
10	Pcyt1b
10	Pdcd10
10	Pdcd2l
10	Pddc1
10	Pde4a
10	Pdgfra
10	Pdha1
10	Pdhx
10	Pdia6
10	Pdik1l
10	Pdk1
10	Pdk4
10	Pdp2
10	Pds5a
10	Pdxdc1
10	Pdzd8
10	Peg3
10	Peli1
10	Peli2
10	Per1
10	Pet112l
10	Pex1
10	Pex10
10	Pex13
10	Pex19
10	Pex5
10	Pfkfb3
10	Pfkl
10	Pgap1
10	Pgap1
10	Pgcp
10	Pggt1b
10	Pgm2l1
10	Pgm3
10	Phc1
10	Phf19
10	Phf3
10	Phip
10	Phlda3
10	Phrf1

10	Phtf2
10	Pi15
10	Pi4k2a
10	Pi4kb
10	Piga
10	Pigg
10	Pigl
10	Pigm
10	Pigo
10	Pik3c2a
10	Pik3r3
10	Pink1
10	Pip4k2b
10	Pitpnb
10	Pitpnc1
10	Pitpnm2
10	Pitrm1
10	Pitx3
10	Pkia
10	Pkmyt1
10	Pla2g4b
10	Plaa
10	Plbd2
10	Plcb4
10	Plcl2
10	Plekha8
10	Plekhf1
10	Plekhg1
10	Plekhg3
10	Plekho1
10	Plk1
10	Plp1
10	Plscr1
10	Plscr3
10	Plxnb1
10	Pml
10	Pmpca
10	Pms2
10	Pnpla8
10	Poc5
10	Pogk
10	Pogz
10	Polr2b
10	Polr2c
10	Polr2d
10	Polr2g
10	Polr2l
10	Polr3d

10	Polr3g
10	Polrmt
10	Pomgnt1
10	Postn
10	Ppap2a
10	Ppapdc1b
10	Ppargc1a
10	Ppfia3
10	Ppil2
10	Ppil4
10	Ppm1l
10	Ppme1
10	Ppox
10	Ppp1r14b
10	Ppp1r3f
10	Ppp1r8
10	Ppp2r3a
10	Ppp2r3c
10	Ppp3ca
10	Ppp3cb
10	Ppp4r1
10	Ppp6c
10	Ppt1
10	Ppwd1
10	Prcp
10	Prdm10
10	Prdm4
10	Prdm5
10	Prdm8
10	Preb
10	Prim1
10	Prkag3
10	Prkg1
10	Procr
10	Prosapip1
10	Prpf31
10	Prr14
10	Prr5l
10	Prr7
10	Prrg4
10	Prrx1
10	Prune2
10	Psma2
10	Psmc3ip
10	Psmd5
10	Pstk
10	Pstpip2
10	Ptar1

10	Ptdss1
10	Ptgr2
10	Ptpla
10	Ptplad2
10	Ptpn11
10	Ptpn2
10	Ptpn23
10	Ptpn4
10	Ptpn9
10	Pttg1
10	Puf60
10	Purg
10	Pus7
10	Pvrl3
10	Pwwp2a
10	Pxk
10	Pxmp4
10	Pycr2
10	Qpctl
10	Qtrtd1
10	R3hdm2
10	Rab11fip1
10	Rab11fip3
10	Rab14
10	Rab20
10	Rab22a
10	Rab24
10	Rab30
10	Rab32
10	Rab34
10	Rab38
10	Rab39b
10	Rab3a
10	Rab3d
10	Rab3gap1
10	Rab3gap2
10	Rab43
10	Rab6a
10	Rab6b
10	Rabgap1
10	Rabggta
10	Rabl3
10	Rad17
10	Rad23a
10	Rad51l3
10	Rad54l
10	Rad54l2
10	Radil

10	Rae1
10	Raet1a
10	Raet1b
10	Raet1c
10	Raet1d
10	Ranbp1
10	Rap1a
10	Rasa3
10	Rasal2
10	Rassf4
10	Raver1
10	Rb1cc1
10	Rbbp8
10	Rbm12
10	Rbm15
10	Rbm15b
10	Rbm27
10	Rbm4b
10	Rbmx
10	Rbmx2
10	Rbmxl1
10	Rcan3
10	Rcl1
10	Rcn2
10	Rcor3
10	Reep4
10	Rell1
10	Rest
10	Ret
10	Rev1
10	Rev3l
10	Rfc1
10	Rfwd2
10	Rfxank
10	Rgma
10	Rgs17
10	Rhoj
10	Ric8
10	Ric8b
10	Rif1
10	Rilpl2
10	Rint1
10	Rit1
10	Rlf
10	Rmi1
10	Rmnd5a
10	Rnaseh1
10	Rnaseh2c

10	Rnasel
10	Rnf103
10	Rnf111
10	Rnf121
10	Rnf125
10	Rnf146
10	Rnf151
10	Rnf19a
10	Rnf19b
10	Rnf2
10	Rnf215
10	Rnf216
10	Rnf220
10	Rnf24
10	Rnf7
10	Rnft1
10	Rnft2
10	Rnpc3
10	Rnps1
10	Robo2
10	Rock2
10	Ror2
10	Rpain
10	Rpe
10	Rpgrip1l
10	Rpl12
10	Rpl13a
10	Rpl15
10	Rpl18a
10	Rpl30
10	Rpl36
10	Rpl39
10	Rpl7
10	Rpp14
10	Rps15
10	Rps16
10	Rps25
10	Rps27
10	Rps4x
10	Rps6
10	Rps6ka4
10	Rps6ka5
10	Rps7
10	Rpusd2
10	Rqcd1
10	Rragd
10	Rrm2b
10	Rrnad1

10	Rrp36
10	Rsad1
10	Rsbn1l
10	Rsf1
10	Rsl24d1
10	Rtkn2
10	Rufy2
10	Rxrb
10	Rybp
10	S100a11
10	S100a7a
10	S1pr1
10	Sac3d1
10	Sacm1l
10	Sap25
10	Sar1b
10	Sars2
10	Sart1
10	Sass6
10	Satb2
10	Sbno1
10	Scaf4
10	Scarf2
10	Schip1
10	Scn5a
10	Scx
10	Scyl1
10	Scyl3
10	Sdc3
10	Sdccag8
10	Sdf2l1
10	Sdpr
10	Sdr39u1
10	Sec13
10	Sec22a
10	Sec61g
10	Sec62
10	Secisbp2
10	Seh1l
10	Selk
10	Sema3a
10	Sema3c
10	Sema5a
10	Sema6c
10	Sema6d
10	Senp6
10	Sept5
10	Serac1

10	Serf1
10	Serinc1
10	Serpinb1a
10	Serpinb8
10	Serpinb9d
10	Serpine1
10	Serpine2
10	Sertad4
10	Sestd1
10	Setx
10	Sft2d2
10	Sft2d3
10	Sgms1
10	Sgol2
10	Sgsh
10	Sh3bgrl2
10	Sh3bp5l
10	Shc2
10	Shisa4
10	Sigirr
10	Siglec15
10	Sip1
10	Sipa1
10	Sirpa
10	Sirt1
10	Slc12a2
10	Slc12a7
10	Slc15a4
10	Slc16a13
10	Slc17a5
10	Slc1a5
10	Slc22a23
10	Slc25a10
10	Slc25a12
10	Slc25a44
10	Slc25a5
10	Slc2a9
10	Slc30a6
10	Slc30a9
10	Slc35a3
10	Slc35b3
10	Slc35d1
10	Slc36a4
10	Slc37a3
10	Slc38a9
10	Slc40a1
10	Slc43a2
10	Slc4a8

10	Slc7a1
10	Slc7a2
10	Slc7a6os
10	Slc9a2
10	Slc9a3r1
10	Slit2
10	Slmo1
10	Sltm
10	Smad3
10	Smad7
10	Smarcc1
10	Smc2
10	Smc3
10	Smc6
10	Smchd1
10	Smek1
10	Smg1
10	Smg7
10	Smo
10	Smpd4
10	Snai2
10	Snap23
10	Snap29
10	Snap47
10	Snapc5
10	Snapin
10	Snrnp200
10	Snrnp35
10	Snrpb2
10	Snw1
10	Snx10
10	Snx11
10	Snx17
10	Snx19
10	Snx2
10	Snx25
10	Snx29
10	Snx33
10	Soat1
10	Sobp
10	Socs7
10	Sorbs1
10	Sox4
10	Sp3
10	Sp8
10	Spata7
10	Spats2
10	Specc1l

10	Spen
10	Spg21
10	Spg7
10	Sphk2
10	Spice1
10	Spin4
10	Spink10
10	Spna2
10	Spock2
10	Spopl
10	Spred3
10	Spty2d1
10	Srfbp1
10	Srgap1
10	Srgn
10	Srl
10	Srrm1
10	Srsf10
10	Ss18l1
10	Ssbp1
10	Ssbp2
10	Ssfa2
10	Ssh3
10	Ssr4
10	St3gal2
10	Stag1
10	Stard10
10	Stard13
10	Stard5
10	Stat1
10	Stil
10	Stim2
10	Stk11ip
10	Stk16
10	Stk25
10	Stk36
10	Stk38l
10	Stk4
10	Stmn4
10	Stoml1
10	Stoml2
10	Ston1
10	Ston2
10	Stra13
10	Stradb
10	Strbp
10	Stub1
10	Stx17

10	Stx6
10	Stx8
10	Stxbp2
10	Stxbp3a
10	Styx
10	Sub1
10	Suclg2
10	Sugp1
10	Sulf1
10	Sumf1
10	Sumo3
10	Sun1
10	Suox
10	Supt4h1
10	Supt7l
10	Suz12
10	Synpo
10	Syt12
10	Sytl5
10	Tab3
10	Tacc1
10	Tacc2
10	Tacc3
10	Taco1
10	Taf10
10	Taf11
10	Taf2
10	Taf4b
10	Taf7
10	Tanc2
10	Taok3
10	Tardbp
10	Tax1bp1
10	Tbc1d17
10	Tbc1d24
10	Tbc1d25
10	Tbc1d9
10	Tbck
10	Tbl3
10	Tbp
10	Tbx18
10	Tceb3
10	Tcerg1
10	Tcf20
10	Tcf25
10	Tcf3
10	Tcf7l2
10	Tchp

10	Tcp11l1
10	Tctn1
10	Tctn3
10	Tdrkh
10	Tead4
10	Terf1
10	Terf2
10	Tesk2
10	Tet2
10	Tex2
10	Tex9
10	Tfpi
10	Tgfb3
10	Tgfbr1
10	Tgfbrap1
10	Tgoln1
10	Thada
10	Thbs2
10	Thg1l
10	Thnsl1
10	Thoc1
10	Thoc2
10	Thop1
10	Thumpd2
10	Tia1
10	Tiam1
10	Tigd2
10	Tigd5
10	Timeless
10	Timm17a
10	Timp3
10	Tirap
10	Tlcd1
10	Tle3
10	Tln2
10	Tmed4
10	Tmem104
10	Tmem108
10	Tmem109
10	Tmem11
10	Tmem111
10	Tmem129
10	Tmem160
10	Tmem170
10	Tmem181a
10	Tmem182
10	Tmem185b
10	Tmem186

10	Tmem194b
10	Tmem201
10	Tmem202
10	Tmem203
10	Tmem218
10	Tmem38a
10	Tmem41a
10	Tmem41b
10	Tmem43
10	Tmem47
10	Tmem66
10	Tmem67
10	Tmem70
10	Tmem86b
10	Tmem87a
10	Tmem93
10	Tmod1
10	Tmod3
10	Tmx4
10	Tnfrsf10b
10	Tnfrsf1b
10	Tnfrsf21
10	Tnfrsf22
10	Tnfrsf23
10	Tnfrsf4
10	Tnik
10	Tnks
10	Tnrc6b
10	Tob1
10	Tob2
10	Top2b
10	Top3a
10	Topors
10	Tor1a
10	Tpk1
10	Tpp1
10	Tpst1
10	Tradd
10	Traf4
10	Traip
10	Trak2
10	Trap1
10	Trappc8
10	Trerf1
10	Trim13
10	Trim24
10	Trim26
10	Trit1

10	Trmt11
10	Trmt112
10	Trmt12
10	Trmt5
10	Trmt61a
10	Trnau1ap
10	Trove2
10	Trp53rk
10	Trpc1
10	Trps1
10	Trrap
10	Tsc22d2
10	Tsg101
10	Tslp
10	Tspan14
10	Tspyl1
10	Tssc1
10	Tstd2
10	Ttc15
10	Ttc27
10	Ttc39c
10	Ttc9c
10	Ttl
10	Ttll12
10	Ttll7
10	Tuba1b
10	Tubb2b
10	Tube1
10	Tubgcp3
10	Tubgcp6
10	Tusc2
10	Txndc12
10	Txndc15
10	Txnl4b
10	Tyms
10	U2af1
10	Uba3
10	Uba52
10	Ubap1
10	Ubap2
10	Ubap2l
10	Ubash3b
10	Ube2cbp
10	Ube2i
10	Ube2j2
10	Ube2v2
10	Ube2z
10	Ube3a

10	Ube4a
10	Ubl3
10	Ubp1
10	Ubqln2
10	Ubxn2b
10	Ubxn6
10	Ubxn8
10	Uckl1
10	Ufsp2
10	Ugdh
10	Uggt2
10	Uhmk1
10	Uhrf1
10	Unc5b
10	Upf1
10	Uprt
10	Uqcr11
10	Urgcp
10	Uros
10	Usp13
10	Usp16
10	Usp24
10	Usp25
10	Usp28
10	Usp3
10	Usp31
10	Usp32
10	Usp33
10	Usp34
10	Usp36
10	Usp40
10	Usp45
10	Usp46
10	Usp49
10	Ust
10	Utp11l
10	Utp18
10	Utp20
10	Utp6
10	Utrn
10	Uvrag
10	Uxs1
10	Uxt
10	Vamp4
10	Vangl1
10	Vangl2
10	Vasn
10	Vav2

10	Vcp
10	Vdac3
10	Vprbp
10	Vps29
10	Vps37a
10	Vps39
10	Vps41
10	Vps52
10	Vti1a
10	Vti1b
10	Vwa1
10	Vwa5a
10	Wars2
10	Wbp2
10	Wbscr16
10	Wbscr27
10	Wdfy3
10	Wdr12
10	Wdr33
10	Wdr35
10	Wdr36
10	Wdr41
10	Wdr45
10	Wdr59
10	Wdr60
10	Wdr7
10	Wipf2
10	Wipi2
10	Wiz
10	Wnt10b
10	Wnt4
10	Wnt5a
10	Wnt7b
10	Wsb2
10	Wtip
10	Wwp1
10	X99384
10	Xbp1
10	Xdh
10	Xiap
10	Xpa
10	Xrn2
10	Yes1
10	Yif1b
10	Yipf2
10	Ythdc2
10	Ythdf2
10	Ywhaq

10	Zadh2
10	Zbed3
10	Zbtb1
10	Zbtb11
10	Zbtb24
10	Zbtb33
10	Zbtb38
10	Zbtb43
10	Zbtb7a
10	Zbtb7c
10	Zbtb8os
10	Zbtb9
10	Zc3h10
10	Zc3h12a
10	Zc3h12c
10	Zc3hav1l
10	Zcchc11
10	Zcchc24
10	Zcchc6
10	Zdhhc16
10	Zfand1
10	Zfand2a
10	Zfat
10	Zfc3h1
10	Zfhx3
10	Zfp1
10	Zfp101
10	Zfp110
10	Zfp111
10	Zfp113
10	Zfp12
10	Zfp120
10	Zfp143
10	Zfp157
10	Zfp239
10	Zfp251
10	Zfp259
10	Zfp27
10	Zfp28
10	Zfp280b
10	Zfp282
10	Zfp287
10	Zfp295
10	Zfp3
10	Zfp318
10	Zfp324
10	Zfp35
10	Zfp354a

10	Zfp362
10	Zfp384
10	Zfp410
10	Zfp420
10	Zfp422
10	Zfp426
10	Zfp445
10	Zfp512
10	Zfp526
10	Zfp53
10	Zfp553
10	Zfp560
10	Zfp563
10	Zfp574
10	Zfp58
10	Zfp595
10	Zfp60
10	Zfp651
10	Zfp652
10	Zfp653
10	Zfp654
10	Zfp661
10	Zfp664
10	Zfp668
10	Zfp672
10	Zfp687
10	Zfp69
10	Zfp692
10	Zfp707
10	Zfp709
10	Zfp71-rs1
10	Zfp748
10	Zfp758
10	Zfp759
10	Zfp772
10	Zfp777
10	Zfp799
10	Zfp800
10	Zfp81
10	Zfp827
10	Zfp839
10	Zfp846
10	Zfp862
10	Zfp867
10	Zfp870
10	Zfp882
10	Zfp90
10	Zfp930

10	Zfp938
10	Zfp945
10	Zfp952
10	Zfp955b
10	Zfp964
10	Zfpm1
10	Zfx
10	Zfyve16
10	Zfyve21
10	Zfyve27
10	Zhx2
10	Zic1
10	Zkscan1
10	Zmat5
10	Zmiz1
10	Zmym5
10	Znf512b
10	Znrf3
10	Zranb3
10	Zrsr2
10	Zscan12
10	Zscan29
10	Zswim6
10	Zwint
10	Zxdb
10	Zxdc
10	I7Rn6
